# Supplementary material for: Changing language input following market integration in a Yucatec Mayan community
Source: PLoS One. 2021 Jun 21;16(6):e0252926. doi: 10.1371/journal.pone.0252926 (PMC8216532; doi:10.1371/journal.pone.0252926)
Supplement: S1 Fig — (DOCX) [file pone.0252926.s001.docx]

**
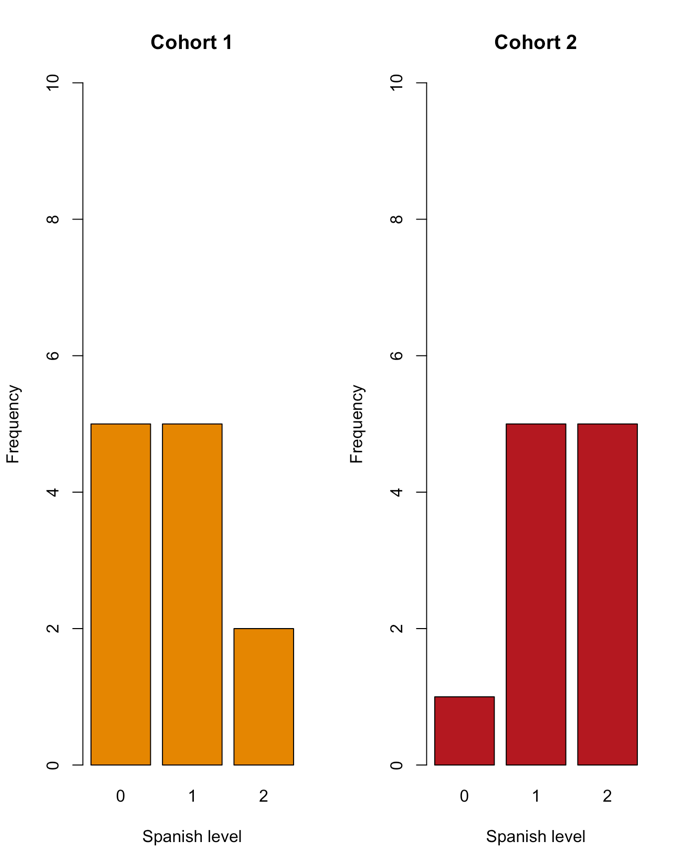
S1 Fig.**

Spanish level of the primary caregivers of the 14 infants recorded in Cohort 1 (left) and the 15 infants recorded in Cohort 2 (right).
