# Supplementary material for: Changing language input following market integration in a Yucatec Mayan community
Source: PLoS One. 2021 Jun 21;16(6):e0252926. doi: 10.1371/journal.pone.0252926 (PMC8216532; doi:10.1371/journal.pone.0252926)
Supplement: S2 Fig — The solid lines are posterior means and the shaded regions are 80% HPDIs. (DOCX) [file pone.0252926.s002.docx]

**
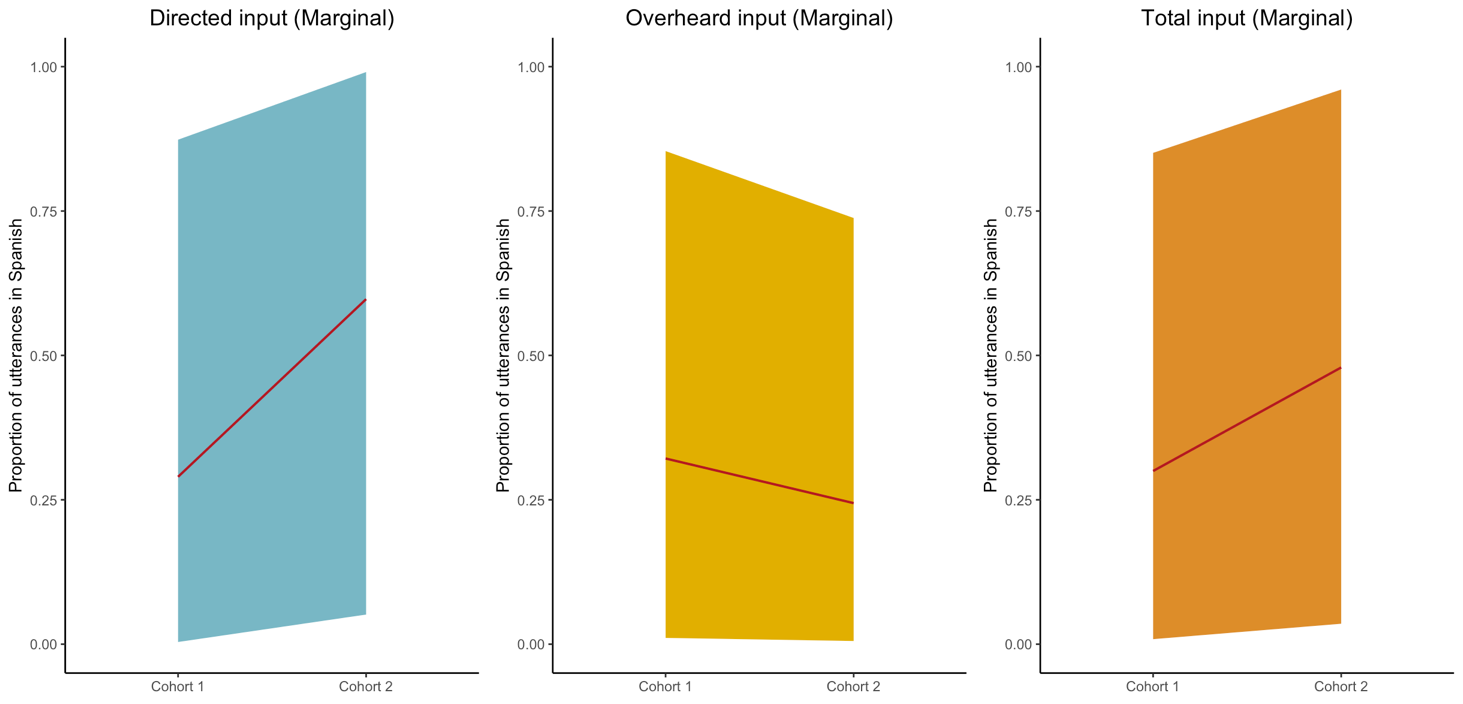
S2 Fig.**

Marginal posterior distribution of changes in the proportion of utterances in Spanish directed to infants (left), overheard by infants (middle) and received by infants overall (right) taking into consideration variation across the different villages. The solid lines are posterior means and the shaded regions are 80% HPDIs
