# Supplementary material for: Changing language input following market integration in a Yucatec Mayan community
Source: PLoS One. 2021 Jun 21;16(6):e0252926. doi: 10.1371/journal.pone.0252926 (PMC8216532; doi:10.1371/journal.pone.0252926)
Supplement: S3 Fig — These were obtained by averaging from 12000 samples from the posterior distribution (setting the standard deviations for the varying intercepts to 0). From top-left to bottom-right: Directed input from primary caregiver, directed input from adults, directed input from children. (DOCX) [file pone.0252926.s003.docx]

**
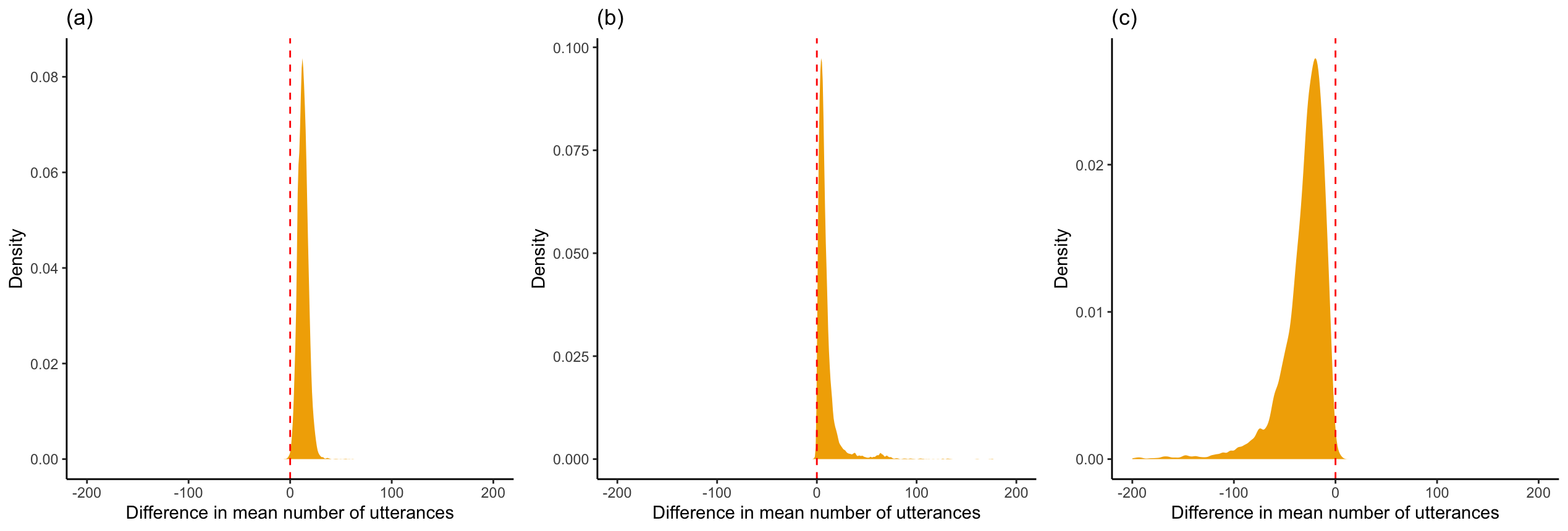
S3. Fig**

Posterior predictive distribution of the mean difference in number of utterances of each type received by the average child from 2007 to 2013, as obtained from the Zero-Inflated Poisson model including “Cohort” as predictor variable and the number of utterances of each type as response variable but excluding those infants from cohort 1 with ages of 23 or 24 months. These were obtained by averaging from 12000 samples from the posterior distribution (setting the standard deviations for the varying intercepts to 0). From top-left to bottom-right: Directed input from primary caregiver, directed input from adults, directed input from children
