# Supplementary material for: Changing language input following market integration in a Yucatec Mayan community
Source: PLoS One. 2021 Jun 21;16(6):e0252926. doi: 10.1371/journal.pone.0252926 (PMC8216532; doi:10.1371/journal.pone.0252926)
Supplement: S1 Table — (DOCX) [file pone.0252926.s004.docx]

**S1 Table**. Descriptive statistics of the primary caregivers of the 14 infants recorded in Cohort 1 (left) and the 15 infants recorded in Cohort 2 (right).

|  | **Cohort 1** | | **Cohort 2** | |
| --- | --- | --- | --- | --- |
|  | **Age at first birth** | **Years in education** | **Age at first birth** | **Years in education** |
| **Min.** | 14 | 1 | 15 | 1 |
| **Max.** | 25 | 5 | 27 | 8 |
| **Median** | 20 | 1 | 18 | 5 |
| **Mean** | 20.08 | 2.17 | 19.60 | 4.09 |
| **SE mean** | 0.87 | 0.47 | 1.34 | 0.76 |
| **Variance** | 9.17 | 2.70 | 17.82 | 6.29 |
| **Std dev.** | 3.03 | 1.64 | 4.22 | 2.51 |
