# Supplementary material for: Changing language input following market integration in a Yucatec Mayan community
Source: PLoS One. 2021 Jun 21;16(6):e0252926. doi: 10.1371/journal.pone.0252926 (PMC8216532; doi:10.1371/journal.pone.0252926)
Supplement: S2 Table — (DOCX) [file pone.0252926.s005.docx]

**S2 Table.** Descriptive statistics of infants recorded in Cohort 1 (left) and Cohort 2 (right).

|  | **Cohort 1** |  | **Cohort 2** |  |
| --- | --- | --- | --- | --- |
|  | **Age (months)** | **Older siblings** | **Age (months)** | **Older siblings** |
| **Min.** | 16.1 | 0 | 16.1 | 0 |
| **Max.** | 24.44 | 9 | 21.15 | 7 |
| **Median** | 22.37 | 4 | 18.02 | 2 |
| **Mean** | 20.88 | 3.89 | 18.17 | 3.18 |
| **SE mean** | 0.35 | 0.56 | 0.36 | 0.76 |
| **95% CI mean** | 0.67 | 1.17 | 0.68 | 1.69 |
| **Variance** | 7.58 | 5.88 | 1.83 | 6.36 |
| **Std dev.** | 2.75 | 2.42 | 1.35 | 2.52 |
