# Supplementary material for: Changing language input following market integration in a Yucatec Mayan community
Source: PLoS One. 2021 Jun 21;16(6):e0252926. doi: 10.1371/journal.pone.0252926 (PMC8216532; doi:10.1371/journal.pone.0252926)
Supplement: S3 Table — (DOCX) [file pone.0252926.s006.docx]

**S3 Table.** Descriptive statistics of all female adults interviewed in 2019 (n=83) .

|  | **Age** | **Years in education** | **No. children** | **Age at birth of first child** | |
| --- | --- | --- | --- | --- | --- |
| **Min.** | 14 | 1 | 0 | | 10 |
| **Max.** | 79 | 12 | 11 | | 33 |
| **Range** | 65 | 11 | 11 | | 23 |
| **Median** | 37 | 8 | 3 | | 20.5 |
| **Mean** | 37.4 | 6.14 | 4.01 | | 20.87 |
| **SE mean** | 1.52 | 0.41 | 0.35 | | 0.5 |
| **95% CI mean** | 3.02 | 0.82 | 0.69 | | 0.99 |
| **Variance** | 189.03 | 13.98 | 10.06 | | 17.39 |
| **Std dev.** | 13.75 | 3.74 | 3.17 | | 4.17 |
