# Supplementary material for: Changing language input following market integration in a Yucatec Mayan community
Source: PLoS One. 2021 Jun 21;16(6):e0252926. doi: 10.1371/journal.pone.0252926 (PMC8216532; doi:10.1371/journal.pone.0252926)
Supplement: S4 Table — (DOCX) [file pone.0252926.s007.docx]

**S4 Table.** Descriptive statistics of all male adults interviewed in 2019 (n=46).

|  | **Age** | **Years in education** |
| --- | --- | --- |
| **Min.** | 15 | 0 |
| **Max.** | 76 | 18 |
| **Range** | 61 | 18 |
| **Median** | 27 | 9 |
| **Mean** | 35.98 | 8.51 |
| **SE mean** | 2.54 | 0.60 |
| **95% CI mean** | 5.13 | 1.21 |
| **Variance** | 298.47 | 16.12 |
| **Std dev.** | 17.27 | 4.01 |
