# Supplementary material for: Changing language input following market integration in a Yucatec Mayan community
Source: PLoS One. 2021 Jun 21;16(6):e0252926. doi: 10.1371/journal.pone.0252926 (PMC8216532; doi:10.1371/journal.pone.0252926)
Supplement: S5 Table — (DOCX) [file pone.0252926.s008.docx]

**S5 Table.** Nature of wage work of the adult males (n=28) that participated in wage labour

| **Wage work done by husbands** | **Count** |
| --- | --- |
| Construction work (palapas, builder) | 17 |
| Tourism (Cenote, waiter...) | 5 |
| Extractive industry (coal) | 1 |
| Taxi driver | 1 |
| Other | 5 |
| TOTAL | 28 |
