# Supplementary material for: Changing language input following market integration in a Yucatec Mayan community
Source: PLoS One. 2021 Jun 21;16(6):e0252926. doi: 10.1371/journal.pone.0252926 (PMC8216532; doi:10.1371/journal.pone.0252926)
Supplement: S6 Table — (DOCX) [file pone.0252926.s009.docx]

**S6 Table.** Comparison of Zero-Inflated Poisson models predicting whether the number of utterances heard by infants in one hour had changed across Cohorts with equivalent Poisson models.

|  | **Model type** | **WAIC** | **pWAIC** | **dWAIC** | **Weight** | **SE** | **dSE** |
| --- | --- | --- | --- | --- | --- | --- | --- |
| **Directed input from primary caregiver** | ZIP | 945.6 | 98.7 | 0.0 | 1 | 127.68 | NA |
|  | Poisson | 1349.8 | 135.1 | 404.2 | 0 | 160.49 | 124.84 |
| **Directed input from adults** | ZIP | 1748.7 | 140.6 | 0.0 | 1 | 355.61 | NA |
|  | Poisson | 3376.7 | 334.0 | 1628.1 | 0 | 476.07 | 442.31 |
| **Directed input from children** | ZIP | 2192.1 | 267.6 | 0.0 | 1 | 371.57 | NA |
|  | Poisson | 2282.0 | 298.1 | 89.9 | 0 | 375.22 | 66.82 |
| **Overheard input from primary caregiver** | ZIP | 1036.4 | 104.5 | 0.0 | 1 | 117.46 | NA |
|  | Poisson | 1099.9 | 112.6 | 63.5 | 0 | 116.75 | 54.29 |
| **Overheard input from adults** | ZIP | 1893.7 | 255.6 | 0.0 | 1 | 374.79 | NA |
|  | Poisson | 3135.1 | 375.2 | 1241.4 | 0 | 438.76 | 321.12 |
| **Overheard input from children** | ZIP | 1664.9 | 175.0 | 0.0 | 1 | 133.73 | NA |
|  | Poisson | 2242.4 | 302.4 | 577.6 | 0 | 515.00 | 496.22 |
