# Supplementary material for: Changing language input following market integration in a Yucatec Mayan community
Source: PLoS One. 2021 Jun 21;16(6):e0252926. doi: 10.1371/journal.pone.0252926 (PMC8216532; doi:10.1371/journal.pone.0252926)
Supplement: S7 Table — These were obtained by averaging from 12000 samples from the posterior distribution (setting the standard deviations for the varying intercepts to 0). (DOCX) [file pone.0252926.s010.docx]

**S7 Table:** Posterior predictive mean number of utterances of each type received by the average child from the Zero-Inflated Poisson model including “Cohort” as predictor variable and the number of utterances of each type as response variable. These were obtained by averaging from 12000 samples from the posterior distribution (setting the standard deviations for the varying intercepts to 0).

|  | | **Number of utterances** | | |
| --- | --- | --- | --- | --- |
|  |  | **Cohort1** | **Cohort2** | **Mean difference between cohorts** |
| **Directed input** | Primary caregiver | 99.55 | 76.81 | -22.74 |
|  | Adults | 36.86 | 37.79 | 0.93 |
|  | Children | 346.69 | 165.72 | -180.97 |
| **Overheard input** | Primary caregiver | 29.81 | 51.06 | 21.25 |
|  | Adults | 38.89 | 66.52 | 27.63 |
|  | Children | 104.66 | 93.59 | -11.06 |
