# Supplementary material for: Changing language input following market integration in a Yucatec Mayan community
Source: PLoS One. 2021 Jun 21;16(6):e0252926. doi: 10.1371/journal.pone.0252926 (PMC8216532; doi:10.1371/journal.pone.0252926)
Supplement: S8 Table — “ll” refers to “lower limit” and “ul” to upper limit. (DOCX) [file pone.0252926.s011.docx]

**S8 Table:** Posterior predictive distributions for the changes in proportion of input in Spanish across cohorts for a child from an average village (in this context, average refers to setting the estimates of the standard deviations for the varying intercepts to zero). “ll” refers to “lower limit” and “ul” to upper limit.

|  | | | **Proportion of utterances in Spanish** | | |
| --- | --- | --- | --- | --- | --- |
|  |  |  | Mean | 80% HPDI ll | 80% HPDI ul |
| **Directed** | Primary caregiver | Cohort 1 | 0.10 | 0.01 | 0.23 |
|  |  | Cohort 2 | 0.29 | 0.04 | 0.63 |
|  | Adults | Cohort 1 | 0.01 | 0.00 | 0.02 |
|  |  | Cohort 2 | 0.66 | 0.47 | 0.82 |
|  | Children | Cohort 1 | 0.27 | 0.03 | 0.65 |
|  |  | Cohort 2 | 0.61 | 0.20 | 0.93 |
|  | **Total** | Cohort 1 | 0.21 | 0.03 | 0.47 |
|  |  | Cohort 2 | 0.67 | 0.32 | 0.93 |
| **Overheard** | Primary caregiver | Cohort 1 | 0.17 | 0.01 | 0.46 |
|  |  | Cohort 2 | 0.10 | 0.00 | 0.25 |
|  | Adults | Cohort 1 | 0.06 | 0.00 | 0.14 |
|  |  | Cohort 2 | 0.07 | 0.01 | 0.15 |
|  | Children | Cohort 1 | 0.39 | 0.08 | 0.78 |
|  |  | Cohort 2 | 0.29 | 0.04 | 0.64 |
|  | **Total** | Cohort 1 | 0.27 | 0.07 | 0.55 |
|  |  | Cohort 2 | 0.17 | 0.03 | 0.37 |
| **Total** | **Total** | Cohort 1 | 0.23 | 0.04 | 0.50 |
|  |  | Cohort 2 | 0.48 | 0.16 | 0.81 |
