# Supplementary material for: Changing language input following market integration in a Yucatec Mayan community
Source: PLoS One. 2021 Jun 21;16(6):e0252926. doi: 10.1371/journal.pone.0252926 (PMC8216532; doi:10.1371/journal.pone.0252926)
Supplement: S9 Table — (DOCX) [file pone.0252926.s012.docx]

**S9 Table**: Comparison of Poisson models predicting the number of directed utterances received by infants in one hour.

| **Poisson models (Number of directed utterances)** | **WAIC** | **pWAIC** | **dWAIC** | **weight** | **SE** | **dSE** |
| --- | --- | --- | --- | --- | --- | --- |
| Proportion of input in Spanish x Cohort | 3188.2 | 349.1 | 0 | 1 | 586.83 | NA |
| Proportion of input in Spanish + Cohort | 3404.4 | 351.7 | 216.2 | 0 | 581.69 | 177.7 |
| Cohort | 3422.5 | 327.7 | 234.3 | 0 | 592.24 | 220.78 |
| Intercept - only | 3739.9 | 305.7 | 551.7 | 0 | 722.44 | 398.6 |
