# Supplementary material for: Changing language input following market integration in a Yucatec Mayan community
Source: PLoS One. 2021 Jun 21;16(6):e0252926. doi: 10.1371/journal.pone.0252926 (PMC8216532; doi:10.1371/journal.pone.0252926)
Supplement: S10 Table — The fact that the total is greater than 66 is because some women gave more than one answer to the question. (DOCX) [file pone.0252926.s013.docx]

**S10 Table**: Self-reported beliefs about Spanish acquisition by the 94 adults that were fluent Spanish speakers. The fact that the total is greater than 66 is because some women gave more than one answer to the question.

| **Question** | **Answer** | | **Count** |
| --- | --- | --- | --- |
| **How did you learn Spanish?** | | School | 65 |
|  |  | Worked in city (Valladolid/Cancun) | 9 |
|  |  | Were born in a city (Valladolid/Cancun) | 3 |
|  |  | From their children | 4 |
|  |  | From visiting cities | 3 |
|  |  | Parents talked to them in Spanish | 7 |
|  |  | From listening to others | 3 |
|  |  | **TOTAL** | 94 |
