# Supplementary material for: Changing language input following market integration in a Yucatec Mayan community
Source: PLoS One. 2021 Jun 21;16(6):e0252926. doi: 10.1371/journal.pone.0252926 (PMC8216532; doi:10.1371/journal.pone.0252926)
Supplement: S11 Table — Counts are the number of females that gave a specific answer to the question of whether they thought infants could acquire competences in Yucatec Maya in the same way as those in Spanish. (DOCX) [file pone.0252926.s014.docx]

**S11 Table**: Beliefs by primary caregivers concerning the ways in which infants learnt the different languages. Counts are the number of females that gave a specific answer to the question of whether they thought infants could acquire competences in Yucatec Maya in the same way as those in Spanish.

| **Are Maya and Spanish learnt differently?** | **Count** | **Percentage** |
| --- | --- | --- |
| Not different^a^ | 65 | 51.6 |
| Maya faster/easier^b^ | 30 | 23.8 |
| Maya from overhearing, Spanish you have to teach them/ they need to practice^b^ | 12 | 9.5 |
| Maya from teaching in the home, Spanish overhearing^c^ | 6 | 4.7 |
| Spanish faster/ easier^c^ | 6 | 4.7 |
| If you only speak to them in Spanish, they take longer to speak^d^ | 1 | 0.8 |
| If they know Spanish, they struggle learning Maya (Not vice versa) ^d^ | 1 | 0.8 |
| Maya from teaching, Spanish from practice^d^ | 1 | 0.8 |
| Maya words have more meanings^d^ | 1 | 0.8 |
| Spanish better when they are older^d^ | 1 | 0.8 |
| Not specified^d^ | 2 | 1.6 |
| **Total** | 126 | 100 |

**^a^** Responses coded as suggesting that interviewee believed Maya and Spanish were learnt in the same way

^b^ Responses coded as suggesting that interviewee believed Maya was easier to learn than Spanish

^c^ Responses coded as suggesting that interviewee believed Spanish was easier to learn than Maya

^d^ Responses not informative in indicating whether interviewees believed the relative difficulty of learning each of the languages differed
