# Supplementary material for: Changing language input following market integration in a Yucatec Mayan community
Source: PLoS One. 2021 Jun 21;16(6):e0252926. doi: 10.1371/journal.pone.0252926 (PMC8216532; doi:10.1371/journal.pone.0252926)
Supplement: S12 Table — (DOCX) [file pone.0252926.s015.docx]

**S12 Table.** Raw averages across cohorts of the number of utterances per input type, both overall (rows 1-6) and in Spanish (rows 7-12).

| **Input type** | **Cohort 1** | **Cohort 2** |
| --- | --- | --- |
| Overheard speech from PC | 37.71 | 43.53 |
| Overheard speech from children | 164.95 | 134.13 |
| Overheard speech from adults | 38.71 | 66.80 |
| Directed speech from PC | 64.00 | 67.67 |
| Directed speech from children | 137.29 | 103.13 |
| Directed speech from adults | 38.86 | 23.40 |
| Overheard speech from PC in Spanish | 0.62 | 2.87 |
| Overheard speech from children in Spanish | 8.43 | 9.73 |
| Overheard speech from adults in Spanish | 0.43 | 7.73 |
| Directed speech from PC in Spanish | 3.00 | 19.00 |
| Directed speech from children in Spanish | 4.43 | 57.60 |
| Directed speech from adults in Spanish | 0.33 | 18.53 |
