# Supplementary material for: Changing language input following market integration in a Yucatec Mayan community
Source: PLoS One. 2021 Jun 21;16(6):e0252926. doi: 10.1371/journal.pone.0252926 (PMC8216532; doi:10.1371/journal.pone.0252926)
Supplement: S13 Table — “ll” refers to “lower limit” and “ul” to upper limit. (DOCX) [file pone.0252926.s016.docx]

**Table S13.** Posterior predictive distributions from the Zero Inflated Poisson models assessing changes in the number of utterances received by infants from their primary caregiver across cohorts and as a function of the number of utterances produced for a child from an average village (in this context, average refers to setting the estimates of the standard deviations for the varying intercepts to zero). “ll” refers to “lower limit” and “ul” to upper limit.

| **Predictor** | **Estimate** | **90% HPDI ll** | **90% HPDI ul** |
| --- | --- | --- | --- |
| Intercept | 4.84 | 3.79 | 5.9 |
| Cohort 2 | -0.43 | -0.53 | -0.33 |
| Utterances produced by focal child | -0.01 | -0.01 | 0 |
