# Supplementary material for: Changing language input following market integration in a Yucatec Mayan community
Source: PLoS One. 2021 Jun 21;16(6):e0252926. doi: 10.1371/journal.pone.0252926 (PMC8216532; doi:10.1371/journal.pone.0252926)
Supplement: S1 Text — (DOCX) [file pone.0252926.s018.docx]

**Supplementary information for**

**Changing language input following market integration in a Yucatec Mayan community**

Cecilia Padilla-Iglesias^1,2^*, Amanda L. Woodward^3^, Susan Goldin-Meadow^3^, Laura A. Shneidman^4^*

* Correspondence to: Cecilia Padilla-Iglesias, Laura A. Shneidman

**Email:**  [cecilia.padillaiglesias@uzh.ch](mailto:cecilia.padillaiglesias@uzh.ch); [las@plu.edu](mailto:las@plu.edu)

**Supplementary Information Text**

***Additional description of the villages and how they have changed over the years***

The villages where data for were collected all have a population of 400-600 inhabitants and are located approximately 50km from the urban center of Valladolid. They are all located along the same road within a 15km radius. Hence, the Spanish-speaking urban centers in the area (namely Valladolid, Tulum, Playa del Carmen or Cancún) are virtually equidistant from all of them.

Families from the villages typically live in single room structures that are situated on plots of land that contain one or more nuclear families. The extended family plays a large role in the life of families and it is common for extended family to share in household duties including childcare. In the recordings collected in both 2007/08 and 2013/14 it was common for infants to have interlocutors other than nuclear family members (e.g., cousins, shopkeepers, etc.)

In all those from which infants’ recordings were obtained, primary school instruction is exclusively in Spanish (although Mayan may be employed for managing the classroom, depending on teacher competencies and particularly with younger children ). Similarly, villagers report that at the time of the first cohort of recordings (2007/08) infants used to have books in Mayan at school but that this was no longer true in 2013/14. During fieldwork in 2017, it was noted that all the books used in the local schools were also in Spanish. Due to the remoteness and transportation costs associated with travel to the villages, there is a high turnover of teachers in the village schools (with many teachers only staying the one or two years to obtain the necessary seniority for a more convenient urban placement). Schoolteachers were and are sometimes speakers of Yucatec Maya but more often wer/are not.

In 2007, most children could only complete kindergarten and primary school in their natal village, except in one of the villages, where there was also a secondary school. None of the villages had a highs-chool (bachiller). By 2013 every village had a secondary school. Currently, the “Telebachillerato comunitario” program has also been introduced in all villages, that allows students to complete education within their communities until they are ready to attend university (see Weiss, 2017 for a more detailed description of this program). In addition, whilst in 2007 most homes did not have televisions, by 2013 virtually every house in the villages had one. Television broadcasts are also exclusively in Spanish.

Last, whilst 66% of fathers from infants in Cohort 1 engaged in some form of wage labor (regardless of whether they also worked in agriculture), whilst 82% of those from infants in Cohort 2 did so. Moreover, although 89% of fathers of infants in Cohort 1 had a milpa (i.e. worked in agriculture), only 63% of fathers of infants in Cohort 2 did so, indicating that more adult males are transitioning to a complete reliance on market jobs. The proportion of male heads of household in the second cohort that participated in wage labour did not significantly vary across villages (69.2%, 81.2%, 54.5% and 75.0%; χ²=2.27, df=3, P=0.52).

Most of the adult men that participated in wage labour (73.2%) went away to urban centers during the week and returned to their local villages every weekend. 14.3% of those men who participated in wage labour returned to their village homes every day, compared to 10.7% that did so every two weeks or 1.8% that went away on monthly trips to engage in wage labour. Locals commented that Locals commented that the latter option used to be the most common but that now men can “afford” to come back weekly mainly due to road improvements.

Last, out of the 126 adults interviewed in 2019 regarding their language attitudes, 42.2% (N=35) of the women were fluent Spanish speakers whilst in the case of the men this proportion was 65.2%

(N=30). All 126 were native Yucatec Maya speakers (and hence fluent in the language) except for one female, who had just moved to one of the villages after getting married and reported that the was learning Maya, as it was necessary to integrate in the village.

***Questionnaire used***

The questionnaire attached below is a translated version of the one that was administered to the participants, who could choose whether to answer each of the questions either in Spanish or Yucatec Maya. It was common for them to switch between the languages throughout. The questions were asked either by the first author or a local research assistant (bilingual in Spanish and Yucatec Maya), but both of us were present during all of the interviews.

*Introduction:*

Town and Municipality:

Date and Time:

Name:

Sex:

Age:

Place of birth:

*Language use:*

1. What’s the first language you learnt? (Maya or Spanish)
   1. How well do you speak it? [ ] I don’t speak it [ ] I only understand it [ ] I speak a little [ ] well [ ] very well [ ] fluently/like native
   2. Where did you learn it?
2. What’s the second language you learnt? (Maya, Spanish or none)
   1. How well do you speak it? [ ] I don’t speak it [ ] I only understand it [ ] I speak a little [ ] well [ ] very well [ ] fluently/like native
   2. Where did you learn it?
3. With respect to Spanish:
   1. If you watch a telenovela in Spanish, do you understand everything? How much do you understand?
   2. If the doctor speaks to you in Spanish, do you understand everything? How much do you understand?
   3. If at the market they speak to you in Spanish, do you understand everything? How much do you understand?
4. Do you ever ask someone for help in order to translate from Spanish to Maya?
   1. Who do you ask?
   2. What for?
5. With respect to when do you use Maya and Spanish
   1. What language do you speak most with your parents?
   2. What language do you speak most with your children
      - During the first year?
      - When they are between 1 and 3 years old?
      - From when they are 6 onwards?
   3. What language do you speak most with your husband/wife? Do they speak Mayan/Spanish?
   4. What language do you speak most with your friends?
   5. What language do you speak most with your neighbours?
6. Do you think that it is more important to learn Maya, Spanish or that both languages are equally important?
   1. Why?
   2. What do people need
      - Spanish for?
      - Maya for?
   3. Do you think it is important to speak to children in
      - Spanish?
      - Maya?
      - When? Why? [expand]

*Social variables:*

1. Do you have siblings?
   1. Do they leave here in this village?
2. Are you married? [write name of spouse]
3. How many children do you have
   1. [list names, sex and age of all of them]
4. Was your partner born in this village?
   1. Where?
5. What do you work as? [allow for >1 response]
   1. Where do you work?
6. Does your partner work in the village/outside?
   1. What do they work as?
   2. (If outside) - How often do they leave the village?
   3. (If outside) – When they leave, how long do they leave for each time?
7. Did you go to school?
   1. For how many years?
8. How long have you lived in this village?
9. Have you ever lived somewhere else?
   1. Where?
   2. For how long?
10. How often do you live this town to visit other towns around the area? (never, sometimes, many times…)
    1. How often do you visit Chemax?
    2. Playa del Carmen?
    3. Cancun?
    4. Valladolid?
11. Do you have any friends or family members that live in other towns?
12. Where?
13. Do they ever come to visit?
14. If they do, do they stay at your home?

*Questions regarding whether language behavior affects integration in local community:*

1. Do you receive any money from remittances? From who?
2. How much?
3. How often? [ask for details]
4. If they work in the field (milpa): Does your field produce enough food for the whole family?
5. Do you worry that in the next month your household will have a time when it is not able to buy or produce enough food to eat?
6. Do your children [daughter/son] help at home?
   1. And In the field?
   2. How much?
   3. Do they all help equally? [expand]
   4. At what age do they start helping out?
7. If you have a lot of work in the farm/ at home, could you ask someone for help?
   1. Who?
8. If you need to leave to another town for a day, could you leave your children with someone?
   1. With Whom?
9. Who is the last person that you visited?
   1. When?
10. In the past week, who did you help with
    1. the field?
    2. childcare?
    3. preparing food?
11. In the past week, who
    1. Visited you?
    2. Helped you with the field?
    3. Helped you with childcare?
    4. Helped you preparing food?
12. In the past week, how many times did you buy Pepsi/ fizzy drinks?

*Beliefs about language development*

1. Some people think that you have to teach children language and some people think that children's language just comes out on its own. What do you think?
2. Is it the same for Maya and for Spanish?
3. How do you believe children learn to talk?

*Yucatec Maya version of the questionnaire*

1. Baax yax t’aani ta kana’? [ ] Maya [ ] Español [ ] Tu kap’elil
   1. Buka ka t’aanik? [ ]Min t’aanik, [ ] Chen ka naajtik, [ ] Chen u chan p’iit, [ ]P’iit, [ ]Maloob, [ ] Jach maloob beye mayao’
   2. ¿Tuux ta kana’?
2. Baax le 2do t’aan ta kana’? [ ] Maya [ ] Español [ ] Miix un p’eel
   1. Buka’ u málobil? [ ]Min t’aanik, [ ] Chen ka naajtik, [ ] Chen u chan p’iit, [ ]P’iit, [ ]Maloob, [ ] Jach maloob beye mayao’
   2. ¿Tuux ta kana’?
3. (With regards to Spanish):
   1. Wa a cha’ant u p’eel Telenovela ich español, ¿Ka najtika tu lakal? ¿Buka’ ka najtikí?
   2. Wa juntul médico ku t’anikech ich español, ¿Ka najtika tu lakal? ¿Buka’ ka najtikí?
   3. Wa ti le mercado ka t’ana ich español, ¿Ka najtika tu lakal?¿Buka’ ka najtikí?
4. ¿Tsook wa a k’aatik antaj ti wa max/ wa max ka xiik ta pach ti al u yantech a na’ajtej?
   1. ¿Max tun (preguntar específicamente sobre sus hijos y su pareja)?
   2. ¿Baax yosal?
5. ¿Chen jok a t’aan ich Maya/ Español?
   1. ¿Baax idioma ka mas t’aanik yetel a yumó?
   2. ¿Baax idioma ka mas t’aanik yetel a palalo’?
      1. Tu yaax año
      2. Tak 1 yetel 3 años
      3. Tak yosal 6 años
   3. ¿Baax idioma ka mas t’aanik yetel a wicham/watam? ¿Ku t’ankoba Maya/Español?
   4. ¿ Baax idioma ka mas t’aanik yetel a amigos?
   5. ¿ Baax idioma ka mas t’aanik yetel a vecinos?
6. ¿Bix a wik k’abeta le Maya t’aan/Español?
   1. ¿Baeni?
   2. ¿Baxu bilal ti le mak’oob le
      - Maya
      - Español?
   3. ¿Bix a tuklik k’abeta u t’aanal le mejen palal ich
      - Maya
      - Español?
      - ¿Baeni? [profundizar]
7. ¿Jay tul a palal?
   1. Escribir el nombre, género y edad de todos los hijos e hijas.
8. ¿Wayila’ a watne’?
   1. ¿Tuxil?
9. ¿Baax ku meyajtik? [permitir más de una respuesta]
10. ¿Tu’ux ku meyaj?
11. ¿Waya ku meyaj a nup ich kaje’ wa yana tuux?
    1. ¿Baax ku meyajtik?
    2. (Si trabaja fuera) ¿Cada baax k’in ku bin?
    3. (Si trabaja fuera) ¿Jayp’el kin ku bin chen xiik meyaj?
12. ¿Xook najá?
    1. ¿Jay p’eel años?
13. ¿Jay p’el años tsook a kajtal way te chan kajá?
14. ¿Tsok a kajtal tu jel tuux?
    1. ¿Tuux?
    2. ¿Jay p’eel años?
15. ¿Cada baax k’in ka bin a ximbalte u jeel kajo’ob? (Ma tech, Chen ku tocar, Yaab u tené, etc)
    1. Wa k’abet a bine: ¿Cada baax k’in ka bin
       1. Chemax,
       2. Playa del Carmen,
       3. Cancún,
       4. Valladolid?
16. ¿Yana tech wa max ku kajtal tu jeel mejen kajoob?
    1. ¿Tu’ux?
    2. ¿Cada baax kin ku taloob waye?
    3. Wa ku taloob, ¿Ku jelo’ob ta najil wené?
17. ¿Ku tuxtála tech tak’in/remesas?
    1. ¿Max tuxtik?
    2. ¿Bajux? [preguntar por detalles]
18. Si trabajan el campo: ¿Ku yantal u jantal a palale é ka pak’ó?
19. ¿Ka tukliká chen a wil u k’uchul un p’eel mes ma yanchaj a pk’alí ti a jantí?
20. ¿Bix yanik a jach ojeltik wa yan u yantal u yich a pak’aló tu lak mes?
21. ¿A palaloob ku yantajobá tu meyajil ich naj?
    1. ¿Ich kool?
    2. ¿Buká?
    3. ¿Tu lakal a palal ku yant’kecho parejo? [Profundizar]
    4. ¿Jay p’eel año chen chunuk u yantajoó?
22. Wa jach yaab a meyaj ich a najilé / ich kool ¿ jeel u beytal a t’anik wa max antikeché?
    1. ¿Max?
23. Wa k’aabet a bin yanal kaj de k’iine ¿Je’el u beytal a p’atik a palal iknal wa maxe?
    1. ¿Max ik naal?
24. ¿Max binech a ximbalté jach u tsokak?
    1. ¿Baax k’inak?
25. Ich lé última semana, ¿Max tsook a wantik yetel
    1. Le kool
    2. U palal
    3. U mak’aan un p’eel janal?
26. Ich lé última semana, ¿Max
    1. Tal ximbatkech?
    2. Antech ich kool
    3. Antech yetel a palaloob
    4. Antech a bet un p’eel janal ?
27. Ich lé última semana, ¿Jay teen tsook a manik pepsi?
28. Yan maké ku yaliké k’abet a ka’ansik a mejen palal t’an, yan xam mak aliké tu junoob ku kanikoob. ¿Bix a tuklik?
29. ¿Beyu t’anal le Maya yetel u t’anal le Espanol a waliko’?
30. ¿Bix u kanikoob t’aan le mejen palalob a walikó?

***Coding of the variables from the questionnaire***

1. Spanish level: 0 indicates not being able to understand it or speak it at all; 1 indicates an ability to understand it and speak a little bit; 2 indicates ability to speak the language fluently.
   1. For female heads of household, Spanish level was assigned after asking them a) whether they spoke Spanish, b) how well, c) whether they understood the doctor when he/she spoke in Spanish, d) whether they understood telenovelas that were in Spanish, e) whether they required help in order to translate what the doctor was saying (as health clinics tend to be located in Spanish-speaking urban centres).
   2. For fathers (husbands) and infants their wives reported their level.
      1. In the case of infants, mothers were asked one by one whether each of their infants spoke Spanish/Maya and the level at which they did so.
   3. For the Bayesian multilevel models, I binary coded Spanish level, with 0-1 in the original scale being coded as 0 and 2 being coded as 1 (hence only individuals that are sufficiently fluent in Spanish are coded as Spanish speakers).
2. Maternal age at birth: Age of the child’s mother when she gave birth to them.
3. Whether parents worked outside: Refers to whether the individual in question is engaged in wage labour outside their current village of residence, regardless of whether they also engaged in agricultural or other kinds of subsistence-related work.
4. Care variables: Refer to answers to the question: if you are very busy/ have to leave the village one day, can you leave your infants with anyone? Triangulated with the questions about whether the caregiver had *actually* relied on anyone for childcare in the previous week

***Bayesian inference and Hamiltonian Monte Carlo***

Bayesian inference is computationally intensive and therefore slow but permits greater modelling flexibility. Bayesian inference also allows a better interpretation of differences between parameter estimates relative to a specific value by obtaining the entire posterior distribution for each predictor and showing the highest density intervals (HPDIs), that reveal the narrowest interval containing the specified probability mass. However, the possibilities it allows of adding regularizing prior distributions to prevent extreme observations, of obtaining more informative results (a distribution of every possible parameter as opposed to a single value), and a precise quantification of uncertainty are often worth the increased computational cost (1).

Utilizing Markov Chain Monte Carlo (MCMC) to obtain posterior distributions bypasses the need of assuming that the posterior distribution has a particular shape by sampling directly from it (ref.2, Chapter 9). Our choice of Hamiltonian MCMC stems from the fact that it performs much better than the most common MCMC algorithms, such as Gibbs sampling, which tend to fail in high dimensions (such as when including random intercepts), as they get stuck in local neighborhoods and poorly explore the posterior distribution (3,4).

***Model specifications***

*Bayesian mixed models for changes in total input across cohorts by input type*

The Zero-Inflated Poisson (ZIP) models used for predicting whether the total number of utterances of each kind received by infants had changed across the years took the following form:

y_i_ ∼ ZIPoisson(p_i_, λ_i_)

logit(p_i_) = α_pVILLAGE[i]_ + β_p_C_i_

log(λ_i_) = α_λVILLAGE[i]_ + β_λ_C_i_

α_VILLAGE_ ∼ Normal(α, σ)

α ∼ Normal(0, 10)

β_λ_ ∼ Normal(0, 2)

β_p_ ∼ Beta(2, 2)

σ ∼ HalfCauchy(0, 1)

Where *y*_i_ is the number of utterances received by the child in an hour (the response variable), *p* is the probability of 0, λ is the average rate at which utterances are produced and C is the ‘Cohort’ (factor indicating to which of the two time periods the video belongs to).

We fitted 6 different models where the response variable was the number of utterances of each type: directed input (DI) by primary caregiver, DI by adults other than the primary caregiver, DI by children, overheard input (OI) by primary caregiver, OI by other adults and OI by children. The predictor variable was always ‘Cohort’ as a factor. Random intercepts for each village (α_VILLAGE_) were included to account for the nested structure of the data and associated clustering. In addition, given that the maximum age in cohort 1 was of 24 months whilst in cohort 2 was of 22.4 months, to rule out the possibility that the differences across cohorts were driven by non-overlapping age ranges, we fitted a second set of models of the same type excluding those infants from cohort 1 that were 23 months old or older.

To assess whether the ZIP models used for the analysis were more justified than the equivalent models with Poisson link function, we fitted identical Poisson models to the ZIP ones. Dyadic comparisons are shown in Table S6. In all cases, the ZIP models outperformed the equivalent Poisson ones with a WAIC weight of 1. This is because many infants did not receive particular input types.

*Bayesian mixed models for changes in directed input from primary caregivers as a function of children’s productive vocabulary*

The Zero-Inflated Poisson (ZIP) models used for predicting whether changes across cohorts in the number of directed utterances received by infants could be attributed to differences in children’s productive vocabulary took the exact same form as those in the above section but including as additional predictor the “Number of utterances produced by the focal child in one hour”.

*Bayesian logistic mixed models for proportion of input in Spanish per input type*

We fitted 6 different models, with the same predictors as the above ones. Here, the response variable was the proportion of Spanish utterances out of the total amount of utterances of each type that children received in one hour (DI by primary caregiver, DI by adults, DI by children, OI by primary caregiver, OI by adults, OI by children), as well as 3 additional models where the response variable was the proportion the total input (TI) in Spanish, the proportion of all directed input (TDI) in Spanish and the proportion of all overheard input (TOI) in Spanish.

The models took the form:

Ai ∼ Binomial(n_i_, p_i_) [likelihood]

logit(p_i_) = α_VILLAGE[i]_ + βC_i_ [linear model]

α_VILLAGE_ ∼ Normal(α, σ) [prior for varying intercepts]

α ∼ Normal(0, 1) [prior for α]

β ∼ Normal(0, 2) [prior for β]

σ ∼ HalfCauchy(0, 1) [prior for σ]

Where *A*_i_ is the log-odds of a child receiving an utterance in Spanish (the response variable), *p* is the probability of hearing an utterance in Spanish, *n* is the total number of utterances of that type received by the child, and C is the ‘Cohort’ (factor indicating to which of the two cohorts the video belonged to). Random intercepts for each village (α_VILLAGE_) were also included.

Table S7 shows the posterior predictive distributions for the changes in proportion of input in Spanish across cohorts for a child from an average village (in this context, average refers to setting the estimates of the standard deviations for the varying intercepts to zero).

*Bayesian mixed models for assessing whether an adoption of the language of the surrounding culture has been accompanied by that the majority-culture interaction style*

We used a set of Bayesian mixed models with Poisson link functions where the response variable was the number of directed utterances received by a particular infant in one hour. All models included village-specific intercepts.

We built a null model comprising only the intercept, and three other models, one with only “Cohort” as predictor variable, one with both cohort and proportion of total input in Spanish as predictor, and one comprising the interaction between the two. The model with the interaction outperformed all others (WAIC weight=1).

*Bayesian logistic mixed models predicting whether caregivers’ beliefs about linguistic acquisition affect the linguistic distribution of child-directed input*

We fitted Bayesian logistic mixed models to assess whether mothers’ beliefs about the relative ease of infants to acquire Maya when compared to Spanish affected their decisions over the proportion of input in Spanish they directed at their toddlers. We fitted one model for each cohort which included the proportion of directed input in Spanish that infants received from their primary caregivers in one hour as response variable (out of the total number of directed utterances they received from their primary caregivers in one hour), and whether or not mothers believed that Maya was learnt easier or faster than Spanish as predictor. This variable reflected the results from Table S10 as a binary-coded factor, where “1” was assigned to those that reported Maya as being learnt “easier”, “faster” or that Maya could be learnt from overhearing whilst Spanish needed to be taught.

Model structure was similar as that of models predicting changes in the proportion of input in Spanish received by infants across cohorts.
