# Supplementary material for: Changing language input following market integration in a Yucatec Mayan community
Source: PLoS One. 2021 Jun 21;16(6):e0252926. doi: 10.1371/journal.pone.0252926 (PMC8216532; doi:10.1371/journal.pone.0252926)
Supplement: S1 References — (DOCX) [file pone.0252926.s019.docx]

**SI References**

1. A. Gelman, *et al.*, *Bayesian data analysis* (Chapman and Hall/CRC, 2013).

2. R. McElreath, *Statistical rethinking: texts in statistical science* (Boca Raton, FL: CRC Press, 2015).

3. R. M. Neal, *MCMC using Hamiltonian dynamics, Handbook of Markov Chain Monte Carlo (S. Brooks, A. Gelman, G. Jones, and X.-L. Meng, eds.)* (Chapman & Hall/CRC Press, 2010).

4. M. Betancourt, A conceptual introduction to Hamiltonian Monte Carlo. *ArXiv Prepr. ArXiv170102434* (2017).
