# Supplementary material for: Changing language input following market integration in a Yucatec Mayan community
Source: PLoS One. 2021 Jun 21;16(6):e0252926. doi: 10.1371/journal.pone.0252926 (PMC8216532; doi:10.1371/journal.pone.0252926)
Supplement: Attachment — Submitted filename: PadillaetalReview.pdf [file pone.0252926.s020.pdf]

Padilla et all, “Why local languages disappear.”

Because I was already late, and because I had considerable difficulty entering the page to start to submit my review today (my old PLOS credentials must have been out of date), I will give you a very brief summary opinion.

The paper presents one very significant finding, based on a comparison of two different cohorts of Maya Yucatec speakers in two different periods of language-acquisition studies separated by 6 years. I found this main empirical finding striking and important (although not without some questions). I think the work should be published if only to disseminate this well founded and potentially useful result. It is, in brief, that over the very short 6 year span, there was significant change in the sort of direct language input infant Yucatec Maya language learners received. It went from being largely in Maya to almost entirely in Spanish (with some empirical caveats I will shortly mention). The result is based on two corpora of longitudinal video recordings of natural child-rearing activities with young Yucatec children in the same Yucatec village settings, one with 21 children from 2007/08 (a study already partly described in other publications dating from 2012), and the other from six years later with a slightly smaller number of children. These corpora were transcribed and coded for the amount and language choices observed in both explicit C(hild) D(irected) S(peech), calculated separately for adult vs. child speakers, and other “overheard” speech within the target children’s earshot. The authors quantified the results statistically to show that over the two cohorts there was a marked shift in CDS (although, notably, NOT in “overheard speech) from almost all in Maya to almost all in Spanish. (There was also overall reduction in CDS from all speakers.) This is striking evidence for rapid language shift, indeed, and these are the results that merit publication, in my opinion.

(I have a few questions about even this result, however, not resolved by the supplementary materials, which provide almost no information about these video corpora and their “transcriptions.” A number of at least potentially problematic “coding” issues can thus not be resolved or even addressed. One is the nature of the distinction between child-directed and “overheard” language, or about determining who is saying what to whom in the video recordings, especially if working just from the transcripts rather than the original videos. More problematic, although more or less explicitly addressed, is the definition of what is “Mayan” and what is “Spanish.” What is counted is, apparently, “utterances,” and the authors say they count as Spanish only what is “all in Spanish,” despite the ubiquitous presence in modern Yucatec of lots of Spanish loans and stock phrases, at various levels of historical assimilation to Maya phonology. It is, thus, a bit unclear what counts as one language or another, as there is considerable bivalency. At least some examples of how these counts were conducted would be reassuring, although the rather stark implied filter [Spanish-only, vs. the rest] suggests that the results must be largely as claimed.)

I find the analysis, methodology, and conclusions of the main argument of the paper, and its associated interview data, considerably less convincing. The paper frames itself within a tradition that places high importance on CDS for language acquisition outcomes, with some caveats about where this CDS comes from. (Is it addressed to infants by adults, or does it originate with other children, who may often be primary caregivers?) It then summarizes several extant hypotheses about what might account for a language shift in such talk, discounting major demographic and economic shifts in the community in question. Is childcare changing so as to change the language input infants receive? Does schooling in a

“majority” language increase over the whole community? Are there changes in parents’ ideas about the importance of native languages vs. “non-local” ones, or about the relative difficulty of acquiring them?

It then goes on to offer results of what I guess can be characterized as an attitude survey (although more is involved than an assessment of language attitudes), using a different methods to address these various proposals about what might reasonably account for rapid language shift in the language of CDS in these communities. As any anthropologist worth her salt will attest, attitude surveys actually often tell you very little about what people actually DO. This is perhaps especially true of surveys asking people about what they did and thought 5 or even 10 years after they did whatever it was. Moreover, a question like “is it ‘more important to learn Maya or Spanish’” is far from neutral, depending one who’s asking, who’s listening, and what you had for breakfast that morning. (Results are even more dubious if, as the table says, “both are important” answers are eliminated as “offering no explanatory power.” The latter clause is certainly true, but such results are not irrelevant to evaluating the eliciting instrument!) Since all of the presented results (but none on the data) are in English, it becomes even more difficult for readers toe valuate the success and reliability of the collected statistics here. (Here is one example: bottom, p. 7, and interviewee is quoted as mentioning “a nephew who only learned how to speak” at age 7—but we have no idea what this means? Mute until 7? Spanish speaking only until 7 [which is what this reader assumed, by contextual inference]. There is not way to evaluate these interviews without something more substantial to go on, and no transcripts are offered of the interviews, to pick out the Mayan from the Spanish, etc.—if any of this matters [which, to the authors, it does not seem to].)

Another conclusion, left very dubious, involves the 4th hypothesis about whether one language ascends because it is thought to be ”harder to learn.” “We confirmed that children whose primary caregivers thought Spanish was harder to learn were more likely to speak to their children in Spanish.” (p. 8). There is first ambiguity about whether “primary caregives” and “mothers” (mentioned in the next line) are the same people. But I am at a loss to know exactly what the questions asked were, or how the answers were evaluated, in the contexts in which they were answered. The claim is repeated in the discussion (last para. P. 9), but no statistics or charts (that I can see) are offered to show this.

The discussion the authors try to address here is important, and I myself have little doub that one of the main changes in the period the authors consider has to do with access to education in rural Mexico, and more specifically to access to education for girls, who otherwise would have been primary caregivers (in the normal sense—i.e., not just mothers) for many infants. But access to education is not the same as getting educated, and one needs to look in more detail at the facts on the ground, not just people tell you about them, before any reasonable conclusions can be drawn.

I would accordingly suggest that the authors reframe the paper so as to foreground the strength and importance of their main empirical result, and temper their enthusiasm for their much more speculative claims about what might account for the change in “direct input,” and how it might relate to various factors they suggest might, in general, account for rapid language shift. This latter question is itself of great importance, both to understanding and to issues about language shift and the survival of minority languages more generally, but the arguments the authors make in this regard are considerably more tenuous and less generalizable than the main empirical finding.
