# Supplementary material for: Changing language input following market integration in a Yucatec Mayan community
Source: PLoS One. 2021 Jun 21;16(6):e0252926. doi: 10.1371/journal.pone.0252926 (PMC8216532; doi:10.1371/journal.pone.0252926)
Supplement: Attachment — Submitted filename: Padilla-Iglesias_PLOS_SM.pdf [file pone.0252926.s021.pdf]

## Supplementary information for

# Why local languages disappear: a study of changing language input following market integration in a Yucatec Mayan community

Cecilia Padilla-Iglesias[1,2]*, Amanda L. Woodward[3], Susan Goldin-Meadow[3], Laura A. Shneidman[4]*

* Correspondence to: Cecilia Padilla-Iglesias, Laura A. Shneidman

**Email:** cecilia.padillaiglesias@uzh.ch; las@plu.edu

**Supplementary Information Text**

***Additional description of the villages and how they have changed over the years***

The villages where data for were collected all have a population of 400-600 inhabitants. In all those from which infants' recordings were obtained, school instruction is exclusively in Spanish (although Mayan may be employed for managing the classroom, particularly with younger infants). Similarly, villagers report that at the time of the first cohort of recordings (2007/08) infants used to have books in Mayan at school but that this was no longer true in 2013/14. During fieldwork in 2017, it was noted that all the books used in the local schools were also in Spanish.
In 2007, children could only complete primary school in their natal village, whilst by 2013 there was a secondary school as well. Currently, the "Telebachillerato comunitario" program has also been introduced, that allows students to complete education within their communities until they are ready to attend university (see Weiss, 2017 for a more detailed description of this program).

Last, whilst 66% of fathers from infants in Cohort 1 engaged in some form of wage labor (regardless of whether they also worked in agriculture), whilst 82% of those from infants in Cohort 2 did so. Moreover, although 89% of fathers of infants in Cohort 1 had a milpa (i.e. worked in agriculture), only 63% of fathers of infants in Cohort 2 did so, indicating that more adult males are transitioning to a complete reliance on market jobs.

***Questionnaire used***

The questionnaire attached below is a translated version of the one that was administered to the participants, who could choose whether to answer each of the questions either in Spanish or Yucatec Mayan. It was common for them to switch between the languages throughout. The questions were asked either by the first author or a local research assistant (bilingual in Spanish and Yucatec Mayan), but both of us were present during all of the interviews.

*Introduction:*

Town and Municipality:
Date and Time:
Name:
Sex:

Age:
Place of birth:

*Language use:*

1. What's the first language you learnt? (Mayan or Spanish)
   a) How well do you speak it? [ ] I don't speak it [ ] I only understand it [ ] I speak a little [ ] well [ ] very well [ ] fluently/like native
   b) Where did you learn it?
2. What's the second language you learnt? (Mayan, Spanish or none)
   a) How well do you speak it? [ ] I don't speak it [ ] I only understand it [ ] I speak a little [ ] well [ ] very well [ ] fluently/like native
   b) Where did you learn it?
3. With respect to Spanish:
   a) If you watch a telenovela in Spanish, do you understand everything? How much do you understand?
   b) If the doctor speaks to you in Spanish, do you understand everything? How much do you understand?
   c) If at the market they speak to you in Spanish, do you understand everything? How much do you understand?
4. Do you ever ask someone for help in order to translate from Spanish to Mayan?
   a) Who do you ask?
   b) What for?
5. With respect to when do you use Mayan and Spanish

   a) What language do you speak most with your parents?
   b) What language do you speak most with your children
      - During the first year?
      - When they are between 1 and 3 years old?
      - From when they are 6 onwards?

      all self report!

   c) What language do you speak most with your husband/wife? Do they speak Mayan/Spanish?
   d) What language do you speak most with your friends?
   e) What language do you speak most with your neighbours?

6. Do you think that it is more important to learn Mayan, Spanish or that both languages are equally important?

a) Why?
b) What do people need
   - Spanish for?
   - Mayan for?
c) Do you think it is important to speak to children in
   - Spanish?
   - Mayan?
   - When? Why? [expand]

*Social variables:*

7. Do you have siblings?
   a) Do they leave here in this village?
8. Are you married? [write name of spouse]
9. How many children do you have
   a) [list names, sex and age of all of them]
10. Was your partner born in this village?
   a) Where?
11. What do you work as? [allow for >1 response]
   a) Where do you work?
12. Does your partner work in the village/outside?
   a) What do they work as?
   b) (If outside) - How often do they leave the village?
   c) (If outside) – When they leave, how long do they leave for each time?
13. Did you go to school?
   a) For how many years?
14. How long have you lived in this village?
15. Have you ever lived somewhere else?
   a) Where?
   b) For how long?
16. How often do you live this town to visit other towns around the area? (never, sometimes, many times…)
   a) How often do you visit Chemax?
   b) Playa del Carmen?
   c) Cancun?
   d) Valladolid?
17. Do you have any friends or family members that live in other towns?

a) Where?

b) Do they ever come to visit?

c) If they do, do they stay at your home?

*Questions regarding whether language behavior affects integration in local community:*

18. Do you receive any money from remittances? From who?

    d) How much?

    e) How often? [ask for details]

19. If they work in the field (milpa): Does your field produce enough food for the whole family?
20. Do you worry that in the next month your household will have a time when it is not able to buy or produce enough food to eat?
21. Do your children [daughter/son] help at home?

    Is there a gender difference here?

    a) And In the field?

    b) How much?

    c) Do they all help equally? [expand]

    d) At what age do they start helping out?

22. If you have a lot of work in the farm/ at home, could you ask someone for help?

    a) Who?

23. If you need to leave to another town for a day, could you leave your children with someone?

    a) With Whom?

24. Who is the last person that you visited?

    a) When?

25. In the past week, who did you help with

    a) the field?

    b) childcare?

    c) preparing food?

26. In the past week, who

    a) Visited you?

    b) Helped you with the field?

    c) Helped you with childcare?

    d) Helped you preparing food?

27. In the past week, how many times did you buy Pepsi/ fizzy drinks?

*Beliefs about language development*

28. Some people think that you have to teach children language and some people think that children's language just comes out on its own. What do you think?
29. Is it the same for Mayan and for Spanish?
30. How do you believe children learn to talk?

***Coding of the variables from the questionnaire***

A) Spanish level: 0 indicates not being able to understand it or speak it at all; 1 indicates an ability to understand it and speak a little bit; 2 indicates ability to speak the language fluently.
    a. For female heads of household, Spanish level was assigned after asking them a) whether they spoke Spanish, b) how well, c) whether they understood the doctor when he/she spoke in Spanish, d) whether they understood telenovelas that were in Spanish, e) whether they required help in order to translate what the doctor was saying (as health clinics tend to be located in Spanish-speaking urban centres).
    b. For fathers (husbands) and infants their wives reported their level.
        i. In the case of infants, mothers were asked one by one whether each of their infants spoke Spanish/Mayan and the level at which they did so.
    c. For the Bayesian multilevel models, I binary coded Spanish level, with 0-1 in the original scale being coded as 0 and 2 being coded as 1 (hence only individuals that are sufficiently fluent in Spanish are coded as Spanish speakers).

B) Maternal age at birth: Age of the child's mother when she gave birth to them.

C) Whether parents worked outside: Refers to whether the individual in question is engaged in wage labour outside their current village of residence, regardless of whether they also engaged in agricultural or other kinds of subsistence-related work.

D) Care variables: Refer to answers to the question: if you are very busy/ have to leave the village one day, can you leave your infants with anyone? Triangulated with the questions about whether the caregiver had *actually* relied on anyone for childcare in the previous week

***Bayesian inference and Hamiltonian Monte Carlo***

Bayesian inference is computationally intensive and therefore slow but permits greater modelling flexibility. Bayesian inference also allows a better interpretation of differences between parameter estimates relative to a specific value by obtaining the entire posterior distribution for each predictor and showing the highest density intervals (HPDIs), that reveal the narrowest interval containing the specified probability mass. However, the possibilities it allows of adding regularizing prior distributions to prevent extreme observations,

of obtaining more informative results (a distribution of every possible parameter as opposed to a single value), and a precise quantification of uncertainty are often worth the increased computational cost (1).

Utilizing Markov Chain Monte Carlo (MCMC) to obtain posterior distributions bypasses the need of assuming that the posterior distribution has a particular shape by sampling directly from it (ref.2, Chapter 9). Our choice of Hamiltonian MCMC stems from the fact that it performs much better than the most common MCMC algorithms, such as Gibbs sampling, which tend to fail in high dimensions (such as when including random intercepts), as they get stuck in local neighborhoods and poorly explore the posterior distribution (3,4).

***Model specifications***

*Bayesian mixed models for changes in total input across cohorts by input type*

The Zero-Inflated Poisson (ZIP) models used for predicting whether the total number of utterances of each kind received by infants had changed across the years took the following form:

$$y_i \sim \text{ZIPoisson}(p_i, \lambda_i)$$
$$\text{logit}(p_i) = \alpha_{p\text{VILLAGE}[i]} + \beta_p C_i$$
$$\log(\lambda_i) = \alpha_{\lambda\text{VILLAGE}[i]} + \beta_\lambda C_i$$
$$\alpha_{\text{VILLAGE}} \sim \text{Normal}(\alpha, \sigma)$$
$$\alpha \sim \text{Normal}(0, 10)$$
$$\beta_\lambda \sim \text{Normal}(0, 2)$$
$$\beta_p \sim \text{Beta}(2, 2)$$
$$\sigma \sim \text{HalfCauchy}(0, 1)$$

Where $y_i$ is the number of utterances received by the child in an hour (the response variable), $p$ is the probability of 0, λ is the average rate at which utterances are produced and C is the 'Cohort' (factor indicating to which of the two time periods the video belongs to).

We fitted 6 different models where the response variable was the number of utterances of each type: directed input (DI) by primary caregiver, DI by adults other than the primary caregiver, DI by children, overheard input (OI) by primary caregiver, OI by other adults and OI by children. The predictor variable was always 'Cohort' as a factor. Random intercepts for each village ($\alpha_{\text{VILLAGE}}$) were included to account for the nested structure of the data and associated clustering.

To assess whether the ZIP models used for the analysis were more justified than the equivalent models with Poisson link function, we fitted identical Poisson models to the ZIP ones. Dyadic comparisons are shown in Table S6. In all cases, the ZIP models outperformed the equivalent Poisson ones with a WAIC weight of 1. This is because many infants did not receive particular input types.

*Bayesian logistic mixed models for proportion of input in Spanish per input type*

We fitted 6 different models, with the same predictors as the above ones. Here, the response variable was the proportion of Spanish utterances out of the total amount of utterances of each type that children received in one hour (DI by primary caregiver, DI by adults, DI by children, OI by primary caregiver, OI by adults, OI by children), as well as 3 additional models where the response variable was the proportion the total input (TI) in Spanish, the proportion of all directed input (TDI) in Spanish and the proportion of all overheard input (TOI) in Spanish.

The models took the form:

| | |
|---|---|
| $Ai \sim \text{Binomial}(n_i, p_i)$ | [likelihood] |
| $\text{logit}(p_i) = \alpha_{\text{VILLAGE}[i]} + \beta C_i$ | [linear model] |
| $\alpha_{\text{VILLAGE}} \sim \text{Normal}(\alpha, \sigma)$ | [prior for varying intercepts] |
| $\alpha \sim \text{Normal}(0, 1)$ | [prior for α] |
| $\beta \sim \text{Normal}(0, 2)$ | [prior for β] |
| $\sigma \sim \text{HalfCauchy}(0, 1)$ | [prior for σ] |

Where $A_i$ is the log-odds of a child receiving an utterance in Spanish (the response variable), $p$ is the probability of hearing an utterance in Spanish, $n$ is the total number of utterances of that type received by the child, and C is the 'Cohort' (factor indicating to which of the two cohorts the video belonged to). Random intercepts for each village ($\alpha_{\text{VILLAGE}}$) were also included.

Table S7 shows the posterior predictive distributions for the changes in proportion of input in Spanish across cohorts for a child from an average village (in this context, average refers to setting the estimates of the standard deviations for the varying intercepts to zero).

*Bayesian mixed models for assessing whether an adoption of the language of the surrounding culture has been accompanied by that the majority-culture interaction style*

We used a set of Bayesian mixed models with Poisson link functions where the response variable was the number of directed utterances received by a particular infant in one hour. All models included village-specific intercepts.

We built a null model comprising only the intercept, and three other models, one with only “Cohort” as predictor variable, one with both cohort and proportion of total input in Spanish as predictor, and one comprising the interaction between the two. The model with the interaction outperformed all others (WAIC weight=1).

*Bayesian logistic mixed models predicting whether caregivers’ beliefs about linguistic acquisition affect the linguistic distribution of child-directed input*

We fitted Bayesian logistic mixed models to assess whether mothers’ beliefs about the relative ease of infants to acquire Mayan when compared to Spanish affected their decisions over the proportion of input in Spanish they directed at their toddlers. We fitted one model for each cohort which included the proportion of directed input in Spanish that infants received from their primary caregivers in one hour as response variable (out of the total number of directed utterances they received from their primary caregivers in one hour), and whether or not mothers believed that Mayan was learnt easier or faster than Spanish as predictor. This variable reflected the results from Table S10 as a binary-coded factor, where “1” was assigned to those that reported Mayan as being learnt “easier”, “faster” or that Mayan could be learnt from overhearing whilst Spanish needed to be taught.

Model structure was similar as that of models predicting changes in the proportion of input in Spanish received by infants across cohorts.

**S1 Fig.**

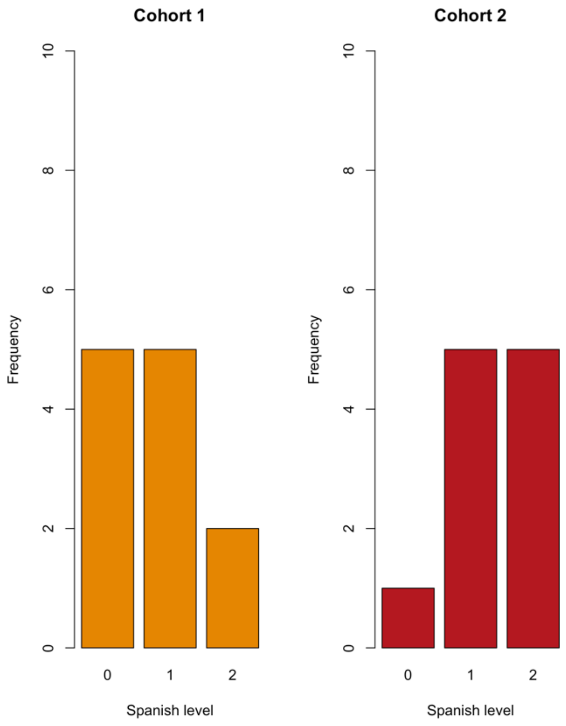


Spanish level of the primary caregivers of the 14 infants recorded in Cohort 1 (left) and the 15 infants recorded in Cohort 2 (right).

**S2 Fig.**

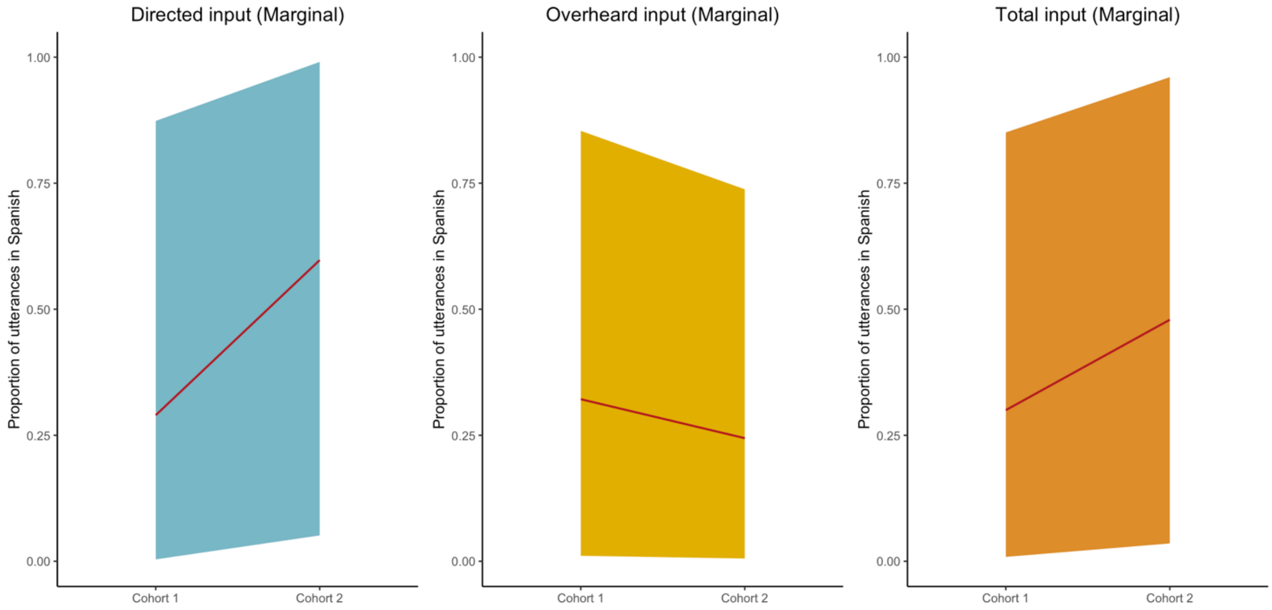


Marginal posterior distribution of changes in the proportion of utterances in Spanish directed to infants (left), overheard by infants (middle) and received by infants overall (right) taking into consideration

variation across the different villages. The solid lines are posterior means and the shaded regions are 80% HPDIs

**S1 Table**. Descriptive statistics of the primary caregivers of the 14 infants recorded in Cohort 1 (left) and the 15 infants recorded in Cohort 2 (right).

| | **Cohort 1** | | **Cohort 2** | |
|---|---|---|---|---|
| | **Age at first birth** | **Years in education** | **Age at first birth** | **Years in education** |
| **Min.** | 14 | 1 | 15 | 1 |
| **Max.** | 25 | 5 | 27 | 8 |
| **Median** | 20 | 1 | 18 | 5 |
| **Mean** | 20.08 | 2.17 | 19.60 | 4.09 |
| **SE mean** | 0.87 | 0.47 | 1.34 | 0.76 |
| **Variance** | 9.17 | 2.70 | 17.82 | 6.29 |
| **Std dev.** | 3.03 | 1.64 | 4.22 | 2.51 |

**S2 Table.** Descriptive statistics of infants recorded in Cohort 1 (left) and Cohort 2 (right).

| | **Cohort 1** | | **Cohort 2** | |
|---|---|---|---|---|
| | **Age (months)** | **Older siblings** | **Age (months)** | **Older siblings** |
| **Min.** | 16.1 | 0 | 16.1 | 0 |
| **Max.** | 24.44 | 9 | 21.15 | 7 |
| **Median** | 22.37 | 4 | 18.02 | 2 |
| **Mean** | 20.88 | 3.89 | 18.17 | 3.18 |
| **SE mean** | 0.35 | 0.56 | 0.36 | 0.76 |
| **95% CI mean** | 0.67 | 1.17 | 0.68 | 1.69 |
| **Variance** | 7.58 | 5.88 | 1.83 | 6.36 |
| **Std dev.** | 2.75 | 2.42 | 1.35 | 2.52 |

**S3 Table.** Descriptive statistics of all female adults interviewed in 2019 (n=83) .

| | **Age** | **Years in education** | **No. children** | **Age at birth of first child** |
|---|---|---|---|---|
| **Min.** | 14 | 1 | 0 | 10 |
| **Max.** | 79 | 12 | 11 | 33 |
| **Range** | 65 | 11 | 11 | 23 |
| **Median** | 37 | 8 | 3 | 20.5 |
| **Mean** | 37.4 | 6.14 | 4.01 | 20.87 |
| **SE mean** | 1.52 | 0.41 | 0.35 | 0.5 |
| **95% CI mean** | 3.02 | 0.82 | 0.69 | 0.99 |
| **Variance** | 189.03 | 13.98 | 10.06 | 17.39 |
| **Std dev.** | 13.75 | 3.74 | 3.17 | 4.17 |

**S4 Table.** Descriptive statistics of all male adults interviewed in 2019 (n=46).

| | **Age** | **Years in education** |
|---|---|---|
| **Min.** | 15 | 0 |
| **Max.** | 76 | 18 |
| **Range** | 61 | 18 |
| **Median** | 27 | 9 |
| **Mean** | 35.98 | 8.51 |
| **SE mean** | 2.54 | 0.60 |
| **95% CI mean** | 5.13 | 1.21 |
| **Variance** | 298.47 | 16.12 |
| **Std dev.** | 17.27 | 4.01 |

**S5 Table.** Nature of wage work of the adult males (n=28) that participated in wage labour

| **Wage work done by husbands** | **Count** |
|---|---|
| Construction work (palapas, builder) | 17 |
| Tourism (Cenote, waiter...) | 5 |
| Extractive industry (coal) | 1 |
| Taxi driver | 1 |
| Other | 4 |
| TOTAL | 28 |

**S6 Table.** Comparison of Zero-Inflated Poisson models predicting whether the number of utterances heard by infants in one hour had changed across Cohorts with equivalent Poisson models.

| | Model type | WAIC | pWAIC | dWAIC | Weight | SE | dSE |
|---|---|---|---|---|---|---|---|
| **Directed input from primary caregiver** | ZIP | 945.6 | 98.7 | 0.0 | 1 | 127.68 | NA |
| | Poisson | 1349.8 | 135.1 | 404.2 | 0 | 160.49 | 124.84 |
| **Directed input from adults** | ZIP | 1748.7 | 140.6 | 0.0 | 1 | 355.61 | NA |
| | Poisson | 3376.7 | 334.0 | 1628.1 | 0 | 476.07 | 442.31 |
| **Directed input from children** | ZIP | 2192.1 | 267.6 | 0.0 | 1 | 371.57 | NA |
| | Poisson | 2282.0 | 298.1 | 89.9 | 0 | 375.22 | 66.82 |
| **Overheard input from primary caregiver** | ZIP | 1036.4 | 104.5 | 0.0 | 1 | 117.46 | NA |
| | Poisson | 1099.9 | 112.6 | 63.5 | 0 | 116.75 | 54.29 |
| **Overheard input from adults** | ZIP | 1893.7 | 255.6 | 0.0 | 1 | 374.79 | NA |
| | Poisson | 3135.1 | 375.2 | 1241.4 | 0 | 438.76 | 321.12 |
| **Overheard input from children** | ZIP | 1664.9 | 175.0 | 0.0 | 1 | 133.73 | NA |
| | Poisson | 2242.4 | 302.4 | 577.6 | 0 | 515.00 | 496.22 |

**S7 Table:** Posterior predictive distributions for the changes in proportion of input in Spanish across cohorts for a child from an average village (in this context, average refers to setting the estimates of the standard deviations for the varying intercepts to zero). "ll" refers to "lower limit" and "ul" to upper limit.

| | | | **Proportion of utterances in Spanish** | | |
|---|---|---|---|---|---|
| | | | Mean | 80% HPDI ll | 80% HPDI ul |
| **Directed** | Primary caregiver | Cohort 1 | 0.10 | 0.01 | 0.23 |
| | | Cohort 2 | 0.29 | 0.04 | 0.63 |
| | Adults | Cohort 1 | 0.01 | 0.00 | 0.02 |
| | | Cohort 2 | 0.66 | 0.47 | 0.82 |
| | Children | Cohort 1 | 0.27 | 0.03 | 0.65 |
| | | Cohort 2 | 0.61 | 0.20 | 0.93 |
| | **Total** | Cohort 1 | 0.21 | 0.03 | 0.47 |
| | | Cohort 2 | 0.67 | 0.32 | 0.93 |
| **Overheard** | Primary caregiver | Cohort 1 | 0.17 | 0.01 | 0.46 |
| | | Cohort 2 | 0.10 | 0.00 | 0.25 |
| | Adults | Cohort 1 | 0.06 | 0.00 | 0.14 |
| | | Cohort 2 | 0.07 | 0.01 | 0.15 |
| | Children | Cohort 1 | 0.39 | 0.08 | 0.78 |
| | | Cohort 2 | 0.29 | 0.04 | 0.64 |
| | **Total** | Cohort 1 | 0.27 | 0.07 | 0.55 |
| | | Cohort 2 | 0.17 | 0.03 | 0.37 |
| **Total** | **Total** | Cohort 1 | 0.23 | 0.04 | 0.50 |
| | | Cohort 2 | 0.48 | 0.16 | 0.81 |

**S8 Table**: Comparison of Poisson models predicting the number of directed utterances received by infants in one hour.

| Poisson models (Number of directed utterances) | WAIC | pWAIC | dWAIC | weight | SE | dSE |
|---|---|---|---|---|---|---|
| Proportion of input in Spanish x Cohort | 3188.2 | 349.1 | 0 | 1 | 586.83 | NA |
| Proportion of input in Spanish + Cohort | 3404.4 | 351.7 | 216.2 | 0 | 581.69 | 177.7 |
| Cohort | 3422.5 | 327.7 | 234.3 | 0 | 592.24 | 220.78 |
| Intercept - only | 3739.9 | 305.7 | 551.7 | 0 | 722.44 | 398.6 |

**S9 Table**: Self-reported beliefs about Spanish acquisition by the 94 adults that were fluent Spanish speakers. The fact that the total is greater than 66 is because some women gave more than one answer to the question.

| Question | Answer | Count |
|---|---|---|
| **How did you learn Spanish?** | School | 65 |
| | Worked in city (Valladolid/Cancun) | 9 |
| | Were born in a city (Valladolid/Cancun) | 3 |
| | From their children | 4 |
| | From visiting cities | 3 |
| | Parents talked to them in Spanish | 7 |
| | From listening to others | 3 |
| | **TOTAL** | 94 |

**S10 Table**: Beliefs by primary caregivers concerning the ways in which infants learnt the different languages. Counts are the number of females that gave a specific answer to the question of whether they thought infants could acquire competences in Yucatec Mayan in the same way as those in Spanish.

| **Are Mayan and Spanish learnt differently?** | **Count** | **Percentage** |
|---|---|---|
| Not different | 65 | 51.6 |
| Mayan faster/easier | 30 | 23.8 |
| Mayan from overhearing, Spanish you have to teach them/ they need to practice | 12 | 9.5 |
| Mayan from teaching in the home, Spanish overhearing | 6 | 4.7 |
| Spanish faster/ easier | 6 | 4.7 |
| If you only speak to them in Spanish, they take longer to speak | 1 | 0.8 |
| If they know Spanish, they struggle learning Maya (Not vice versa) | 1 | 0.8 |
| Mayan from teaching, Spanish from practice | 1 | 0.8 |
| Mayan words have more meanings | 1 | 0.8 |
| Spanish better when they are older | 1 | 0.8 |
| Not specified | 2 | 1.6 |
| **Total** | 126 | 100 |

**SI References**


1. A. Gelman, *et al.*, *Bayesian data analysis* (Chapman and Hall/CRC, 2013).
2. R. McElreath, *Statistical rethinking: texts in statistical science* (Boca Raton, FL: CRC Press, 2015).
3. R. M. Neal, *MCMC using Hamiltonian dynamics, Handbook of Markov Chain Monte Carlo (S. Brooks, A. Gelman, G. Jones, and X.-L. Meng, eds.)* (Chapman & Hall/CRC Press, 2010).
4. M. Betancourt, A conceptual introduction to Hamiltonian Monte Carlo. *ArXiv Prepr. ArXiv170102434* (2017).
