# Supplementary material for: Changing language input following market integration in a Yucatec Mayan community
Source: PLoS One. 2021 Jun 21;16(6):e0252926. doi: 10.1371/journal.pone.0252926 (PMC8216532; doi:10.1371/journal.pone.0252926)
Supplement: Attachment — Submitted filename: PLoS One Review.pdf [file pone.0252926.s022.pdf]

Report: *Why local languages disappear: a study of changing language input following market integration in a Yucatec Mayan community*

This paper examines linguistic input in a bilingual setting in Yucatán over time and language attitudes towards the two languages in question. This data is used to test the hypothesis that language shift away from local languages is driven primarily by parents "devaluing" the local language, with the authors arguing that a mix of other factors better explains the shifts witnessed in this community.

This is an interesting and worthy topic and the core finding of a shift over time in the input children receive is quite interesting and potentially important. However, I'm pretty unclear what the "devaluation" hypothesis actually is, to what extent it's a straw man, and whether there's any substantial positive conclusion to be made about the various alternative factors discussed (e.g. changes in childcare, schooling, parental beliefs about language learning). For these reasons, I cannot recommend this paper for publication. I summarize my basis for this assessment below in A and B.

A. What is "devaluation"?

The paper is structured primarily as an argument against "devaluation" of Maya (or of indigenous and other local languages generally) as the causal factor responsible for shifts in language input. However, I don't understand what the authors conception of "devaluation" is and therefore find it hard to assess this hypothesis and how it might be linked to the data presented.

First off, the extent and ways in which people value or don't value different languages doesn't happen in a vacuum. It is itself consequence of any number of complex social, economic, political, and other factors that all interact with other factors, including the ones discussed as competing hypotheses. For example, parents choice of caregivers could be partially driven by how much they value different languages. Second, the valuing of different languages mostly matters relative to other languages. People's esteem for Maya could be unchanged but if their belief in the value of Spanish increases, this would potentially have similar impact, even though it doesn't seem intuitive to describe this as "devaluing".

Given how I interpret "devaluing", I'm not clear how the data on the input have any clear bearing on it. The aspect of the input data that the authors appear to take as related to this hypothesis seems to be the percentage of CDS by parents that is in the two languages. There might be other conceptions of "devaluing" that make direct predictions about this or there might be ways to motivate a connection between my conception and this data. As someone expert in understudied

languages, I don't find there to be a specific well-known "devaluation" explanation assumed in the literature in the way that the authors seem to imply. As such, **I think it's incumbent upon the authors to explain 3 things: 1) what their conception of "devaluation" is, 2) what interactions is does or does not have with other causal factors discussed here, and 3) why it makes particular predictions about the kinds of data considered here.**

B. Positive proposals are weak

Beyond arguing against the "devaluation" hypothesis, the authors discuss a number of different affirmative proposals for the causes of shifts in language input. Many of theses are quite plausible, but I don't find that the study really makes a very strong empirical case regarding these different alternatives. I think what would be most convincing is if they could have shown individual-level correlations between language attitudes/use and other social variables and pattern of input. Failing this, showing population-level shifts in these attitudes over the time period would be helpful. However, the data in this paper don't seem well-positioned to do either of these things.

Instead, we have data from a 2019 attesting to language attitudes and very cursory ethnographic information about economic, educational, and other changes between 2007/2008 and 2013/2014. The time period being studied here is one with immense changes economically, socially, and technologically both for the world in general and Yucatán in particular. This paper presents very little ethnographic detail of the sort that would be crucial to understanding which of these various factors play more or less important roles. Another demographic factor not asked about and worth considering is whether there are any truly monolingual Maya speakers in regualr social contact with the child and the household. Not knowing the specific towns in question of course, I feel that in 2007 it was likely that many households would have had a grandparent/neighbor/etc. who really didn't speak Spanish well, whereas in 2019 this may be much less likely.

C. Other questions and more minor points

Mayan vs. Mayan

I recognize that the choice between terms "Maya" and "Mayan" is quite fraught and that there are often no hard and fast rules in which terms to use when in many cases. However, I encourage the authors to rethink their choice of terms here. In several cases, "Mayan" is used in places where I would expect

“Maya”. Basically, I think of ‘Mayan’ as meaning something mor like “of or pertaining to the Maya” rather than being Maya. Terms like “Mayan caregivers” don’t really make sense to me given this. This blog post by Laura Martin give a nice summary of these tricky issues: `https://www.mayatraditions.org/maya-vs-mayan-by-laura-martin-ph-d/`

I think the one place where I would more stridently push for a change is in the name of the language itself. The language is called “Maya”, not “Mayan”, whether or not the geographic modifier “Yucatec” is added. Yucatec Mayan simply sounds incorrect to me.

Info on villages

It’d be helpful to have more information on the villages in question. We are told the population and a bit about the economic activities of fathers, but it would be helpful to have a richer picture of the demographics of the towns, how many monolingual Spanish and Maya speaker there are if any, the extent to which “wage labor” here means working outside for extended period of time vs. other things, whether families have members who live more or less full time in Spanish speaking environments such as Cancún.

Also, I assume that the individual villages can’t be named for privacy reasons, but I’m confused by the descriptions here. The villages are described as “in the state of Yucatán about 80 miles to the southwest of Cancun (the closest Spanish-speaking urban centre)”. While it’s fair to say that Cancún is an urban center in a way Playa del Carmen, Valladolid, and Tulum are not, it does seem quite relevant that these are all places that offer ample wage labor opportunities in the tourist industry, many of which (especially the higher paying ones) would be predominantly Spanish or English-based. It’d be helpful to clarify this and also to say something about how far the villages are from such places. A village just outside Valladolid is apt to be very different than one just outside Playa del Carmen or one relatively far from all of these.
