# Supplementary material for: Changing language input following market integration in a Yucatec Mayan community
Source: PLoS One. 2021 Jun 21;16(6):e0252926. doi: 10.1371/journal.pone.0252926 (PMC8216532; doi:10.1371/journal.pone.0252926)
Supplement: Attachment — Submitted filename: PONE-D-20-39416_reviewer.pdf [file pone.0252926.s023.pdf]

PLOS ONE

Why local languages disappear: a study of changing language input following market integration in a Yucatec Mayan community

--Manuscript Draft--

**Manuscript Number:**	PONE-D-20-39416
**Article Type:**	Research Article
**Full Title:**	Why local languages disappear: a study of changing language input following market integration in a Yucatec Mayan community
**Short Title:**	Changing language input following market integration in a Yucatec Mayan community
**Corresponding Author:**	Cecilia Padilla-Iglesias<br>Universitat Zurich<br>Zurich, SWITZERLAND
**Keywords:**	Market integration, Language change, Yucatan, Mexico, Language input, Ethnotheories, Language socialization
**Abstract:**	By the end of the current century, over half of the world's languages are expected to disappear. The replacement of minority languages with surrounding majority tongues is likely due to changes in speech input that is directed to young children. However, there is little work quantifying shifts in input or exploring potential causal factors that may trigger them. Here, we provide the first systematic assessment of the quantity, type, source, and language of the input received by 29 Yucatec Mayan infants born six years apart in communities where increased contact with urban centres has resulted in a greater exposure to the dominant surrounding language, Spanish. Results show that infants from the second cohort received less directed input than infants in the first and, when directly addressed, most of their input was in Spanish. To investigate the mechanisms driving the observed patterns, we interviewed 126 adults from the communities. Against common assumptions, we showed that reductions in Mayan input did not simply result from speakers devaluing the Maya language. Instead, changes in input could be attributed to changes in childcare practices as well as a belief that the acquisition difficulty of each of the languages differed. Our study highlights the need for understanding the drivers of individual behaviour in order to predict the fate of linguistic diversity in the face of socio-demographic and economic changes.
**Order of Authors:**	Cecilia Padilla-Iglesias
	Amanda L. Woodward
	Susan Goldin-Meadow
	Laura A. Shneidman
**Additional Information:**	
**Question**	**Response**
**Financial Disclosure**<br><br>Enter a financial disclosure statement that describes the sources of funding for the work included in this submission. Review the submission guidelines for detailed requirements. View published research articles from *PLOS ONE* for specific examples.<br><br>This statement is required for submission and **will appear in the published article** if	This study was funded by the Department of Archaeology at the University of Cambridge and by a NSF award (BCS-1226113) to Amanda Woodward.

Powered by Editorial Manager® and ProduXion Manager® from Aries Systems Corporation

the submission is accepted. Please make sure it is accurate.<br><br>**Unfunded studies**<br>Enter: *The author(s) received no specific funding for this work.*<br><br>**Funded studies**<br>Enter a statement with the following details:<br>• Initials of the authors who received each award<br>• Grant numbers awarded to each author<br>• The full name of each funder<br>• URL of each funder website<br>• Did the sponsors or funders play any role in the study design, data collection and analysis, decision to publish, or preparation of the manuscript?<br>• **NO** - Include this sentence at the end of your statement: *The funders had no role in study design, data collection and analysis, decision to publish, or preparation of the manuscript.*<br>• **YES** - Specify the role(s) played.<br><br>* typeset	
**Competing Interests**<br><br>Use the instructions below to enter a competing interest statement for this submission. On behalf of all authors, disclose any competing interests that could be perceived to bias this work—acknowledging all financial support and any other relevant financial or non-financial competing interests.<br><br>This statement **will appear in the published article** if the submission is accepted. Please make sure it is accurate. View published research articles from *PLOS ONE* for specific examples.	The authors have declared that no competing interests exist.

**NO authors have competing interests**<br><br>Enter: *The authors have declared that no competing interests exist.*<br><br>**Authors with competing interests**<br><br>Enter competing interest details beginning with this statement:<br><br>*I have read the journal's policy and the authors of this manuscript have the following competing interests: [insert competing interests here]*<br><br>* typeset	
**Ethics Statement**<br><br>Enter an ethics statement for this submission. This statement is required if the study involved:<br><br>• Human participants<br>• Human specimens or tissue<br>• Vertebrate animals or cephalopods<br>• Vertebrate embryos or tissues<br>• Field research<br><br>Write "N/A" if the submission does not require an ethics statement.<br><br>General guidance is provided below. Consult the submission guidelines for detailed instructions. **Make sure that all information entered here is included in the Methods section of the manuscript.**	Informed, written consent was obtained from all adult participants included in the study and from all parents of the recorded children on their behalf. In addition All procedures involved in this study were reviewed and approved by the Ethics Commission of the University of Cambridge as well as the Ethics Committee of the University of Chicago. They are also in accordance with the 1964 Helsinki declaration and its later amendments or comparable ethical standards.

Powered by Editorial Manager® and ProduXion Manager® from Aries Systems Corporation

**Format for specific study types**<br><br>**Human Subject Research (involving human participants and/or tissue)**<br>• Give the name of the institutional review board or ethics committee that approved the study<br>• Include the approval number and/or a statement indicating approval of this research<br>• Indicate the form of consent obtained (written/oral) or the reason that consent was not obtained (e.g. the data were analyzed anonymously)<br><br>**Animal Research (involving vertebrate animals, embryos or tissues)**<br>• Provide the name of the Institutional Animal Care and Use Committee (IACUC) or other relevant ethics board that reviewed the study protocol, and indicate whether they approved this research or granted a formal waiver of ethical approval<br>• Include an approval number if one was obtained<br>• If the study involved *non-human primates*, add *additional details* about animal welfare and steps taken to ameliorate suffering<br>• If anesthesia, euthanasia, or any kind of animal sacrifice is part of the study, include briefly which substances and/or methods were applied<br><br>**Field Research**<br><br>Include the following details if this study involves the collection of plant, animal, or other materials from a natural setting:<br>• Field permit number<br>• Name of the institution or relevant body that granted permission	
**Data Availability**<br><br>Authors are required to make all data underlying the findings described fully available, without restriction, and from the time of publication. PLOS allows rare exceptions to address legal and ethical concerns. See the PLOS Data Policy and FAQ for detailed information.	Yes - all data are fully available without restriction

A Data Availability Statement describing where the data can be found is required at submission. Your answers to this question constitute the Data Availability Statement and **will be published in the article**, if accepted.<br><br>**Important:** Stating 'data available on request from the author' is not sufficient. If your data are only available upon request, select 'No' for the first question and explain your exceptional situation in the text box.<br><br>Do the authors confirm that all data underlying the findings described in their manuscript are fully available without restriction?	
**Describe where the data may be found in full sentences. If you are copying our sample text, replace any instances of XXX with the appropriate details.**<br><br>• If the data are **held or will be held in a public repository**, include URLs, accession numbers or DOIs. If this information will only be available after acceptance, indicate this by ticking the box below. For example: *All XXX files are available from the XXX database (accession number(s) XXX, XXX.*).<br>• If the data are all contained **within the manuscript and/or Supporting Information files**, enter the following: *All relevant data are within the manuscript and its Supporting Information files.*<br>• If neither of these applies but you are able to provide **details of access elsewhere**, with or without limitations, please do so. For example:<br><br>*Data cannot be shared publicly because of [XXX]. Data are available from the XXX Institutional Data Access / Ethics Committee (contact via XXX) for researchers who meet the criteria for access to confidential data.*<br><br>*The data underlying the results presented in the study are available from (include the name of the third party*	All the data required to reproduce the analyses have been attached as supplementary files.

and contact information or URL).<br>• This text is appropriate if the data are owned by a third party and authors do not have permission to share the data.<br><br>* typeset	
Additional data availability information:	

Powered by Editorial Manager® and ProduXion Manager® from Aries Systems Corporation

Manuscript
Click here to access/download;Manuscript;Padilla-Iglesias_plos.docx

Why local languages disappear: a study of changing language input following market integration in a Yucatec Mayan community

Cecilia Padilla-Iglesias[1,2]*, Amanda L. Woodward[3], Susan Goldin-Meadow[3], Laura A. Shneidman[4]*

[1]Leverhulme Centre for Human Evolutionary Studies, Department of Archaeology, University of Cambridge, Cambridge CB2 1QH, United Kingdom

[2]Institute of Anthropology, University of Zurich, Zurich, 8057, Switzerland.

[3]Department of Psychology, University of Chicago, Chicago, IL 60637

[4] Department of Psychology, Pacific Lutheran University, Tacoma, WA 98447

* Correspondence to: Cecilia Padilla-Iglesias, Laura A. Shneidman

Email: cecilia.padillaiglesias@uzh.ch; las@plu.edu

Cecilia Padilla-Iglesias: https://orcid.org/0000-0003-1302-5955

Laura A. Shneidman: https://orcid.org/0000-0001-5981-4015

Abstract

By the end of the current century, over half of the world's languages are expected to disappear. The replacement of minority languages with surrounding majority tongues is likely due to changes in speech input that is directed to young children. However, there is little work quantifying shifts in input or exploring potential causal factors that may trigger them. Here, we provide the first systematic assessment of the quantity, type, source, and language of the input received by 29 Yucatec Mayan infants born six years apart in communities where increased contact with urban centres has resulted in a greater exposure to the dominant surrounding language, Spanish. Results show that infants from the second cohort received less directed input than infants in the first and, when directly addressed, most of their input was in Spanish. To investigate the mechanisms driving the observed patterns, we interviewed 126 adults from the communities. Against common assumptions, we showed that reductions in Mayan input did not simply result from speakers devaluing the Maya language. Instead, changes in input could be attributed to changes in childcare practices as well as a belief that the acquisition difficulty of each of the languages differed. Our study highlights the need for understanding the drivers of individual behaviour in order to predict the fate of linguistic diversity in the face of socio-demographic and economic changes.

Main Text

Introduction

The worlds' linguistic diversity is eroding at an unprecedented rate, as speakers gradually replace lesser used languages with ones in more dominant use (1,2). Decisions over which language caregivers use to address their children has been almost universally considered to be the decisive element in determining this erosion of linguistic diversity (3-8). Parents are assumed to shift input away from minority local languages and towards majority surrounding languages because they believe that dominant languages are socially and economically advantageous for children to learn (8-10). For example, Hill and Hill (11) and Rolstad (12) attribute the decline of Nahuatl speakers in Mexico over the 20th century to parents using Spanish (rather than Nahuatl) with their children in order to prepare them for the newly introduced bilingual schooling policies. However, hypotheses that focus on parental devaluation of native languages as the primary catalyst for language loss fail to take into account several additional factors that could affect the input children receive in the small-scale communities at risk for language loss.

First, a decline in native language input following market integration could be due to changes in childcare practices that shift caregiving away from older children (13). In many cultural communities, it is *not* parents who have traditionally served as infant's primary interlocutors, but rather other children (14-17). If changing market economies result in infants spending less time with older siblings, this could result in less time that infants are engaged in directed conversations overall (including conversations in a local language). Changes in childcare in emerging integrated contexts could result from several factors. For example, the reduction in fertility that often accompanies transitions from subsistence to skill-based economies could mean that young children will have fewer older siblings to care for, and therefore talk to them (18). Moreover, if older children are expected to devote more time to their education, they may have less time to devote to household labour, including childcare (19,20). Consequently, speech directed to young children might face overall decline, and, when combined with the other factors described below, especially affect local language input, even if there is no change in parental beliefs about the value of one language over another. Given the pivotal role of child-directed speech for the subsequent development of linguistic competences (21), these changes in input patterns could gradually result in less and less members of the younger generations becoming proficient in the local language.

Second, during periods of market integration, both parents and older siblings of young children in small-scale communities are more likely than previous generations to have had access to formal schooling provided by the majority community. This access both exposes caregivers to non-local languages (providing them with the ability to use the language in interaction with young children) and also introduces caregivers to the interaction patterns of the dominant culture. Schools typically model a pattern of

interaction and socialization in which children are directly addressed in pedagogical interaction (e.g. 22,23). Caregivers who have been schooled might integrate these socialization patterns into their childcaring practices with respect to both their local and non-local languages (23). Alternatively, they might adopt pedagogical practices more narrowly, reserving school-like directed interactions for interactions in the non-local language (the language of school instruction). If so, directed speech to children would occur more often in the non-local language than the local language, again potentially affecting the relative ability of children to learn each of the languages.

Third, hypotheses arguing that parental devaluation of a native language accounts for language loss have not considered broader cultural ethnotheories about language learning, and how these ethnotheories may differ for native and non-native languages. Rather than undervaluing the local language, parents may use directed speech specifically in a non-local language because they have beliefs about the necessity of doing so. Previous research suggests that in many small-scale communities at greatest risk for language loss development is thought to come from within the child, requiring little outside intervention (8,24,25). In these contexts, directed linguistic input may be viewed as less important for supporting native language transmission than it is in cultural communities where directed teaching is considered critical (26,27). Indeed, Shneidman and Goldin-Meadow (15) found that caregivers from the United States were 7 times more likely to direct speech to their infants than caregivers from rural Yucatec Mayan communities. Other studies have confirmed this general pattern — infants from market-economies are between 2 and 11 times more likely to receive child-directed speech (CDS) than infants from small-scale, subsistence economies (16,28,29).

It is possible, however, that this view that directed speech is not critical for supporting language acquisition is specific to caregivers' own native language. In emerging bilingual contexts adults and older children are more likely to have learned the non-local language later in life and thus may have encountered difficulty in their own learning. This may lead them to the false conclusion that the non-local language is inherently more difficult to learn than the local language (30). Such a belief could result in caregivers using more directed speech to children in the non-local language than the local language, even when they desire that children acquire equal competences in each.

In summary, four factors have been proposed to explain the fact that children receive less directed speech in their native language than in the non-local language when small-scale societies are being integrated into majority cultures: 1) a general devaluing of the native language, 2) changes in child-care practices, 3) changing access to education and 4) specific parental beliefs regarding the importance of directed input for language learning. In order to assess the contributions of these factors, it is necessary to quantify changes in children's input during periods of market integration, and determine how these changes intersect with changing demographics and belief systems.

We address these questions by taking advantage of a "natural experiment" arising in the Yucatán peninsula as a result of economic development, new means of transportation, opportunities for wage labor, and access to education. Using video data collected from natural interactions, we compared the linguistic input received by 16-24-month-old infants growing up in the same Yucatec Mayan villages across two cohorts approximately 6 years apart (n=21 in 2007/08, see ref. 15, and n=15 in 2013/14). Previous work in this community showed that input heard by infants in 2007/2008 was primarily in the local language, Yucatec Maya, and provided by other children rather than adults (15). Here, we ask whether these characteristics shifted in this community after a period of increased market integration. In addition, we conducted structured interviews with 126 adults from the villages (including all of the infant's primary caregivers) in January 2019. These interviews provide demographic information related to the changing economy, educational practices, and care practices, and also shed light on caregiver beliefs and attitudes about language. In particular, they provide insight into how important caregivers thought Mayan vs. Spanish was for their infant's future success, and how difficult they thought the two languages were to learn. We examined whether language input changed over the period of market integration—in quantity, type (directed, overheard), and language choice (Mayan, Spanish) —and whether those changes in language input were related to childcare, educational, and belief practices.

Results

We first used interview data and family histories to assess socio-demographic changes across the six-year period in the focal villages. As expected, there were general differences in variables related to increased contact with the majority culture across cohorts—greater access to education, wage labor, and market goods (SI Text, Table S1-S2, Fig. S1). For example, in 2007, children could only complete primary school in their natal village; by 2013, they could also attend secondary school. Primary school attendance of caregivers had also doubled across cohorts (2007: Mean=2.17, SD=1.64; 2014: Mean=4.09, SD=2.51). However, in line with findings by Schacht et al. (31) and Gaskins (25), many aspects of the villages had not changed during this period: Age of mother at first birth (2007: Mean=20.08, SD=3.03; 2014: Mean=19.60, SD=4.22); Average number of older siblings of focus infants (2007: Mean= 3.89, SD=2.75; 2014: Mean=3.19, SD=2.52); no infant had younger siblings because the target infant was always the youngest of the family (weaning age ranged from 2-2.5 years).

In order to evaluate whether there had been changes in and patterns of linguistic socialization, and the prevalence of Spanish in the speech heard by infants following market integration, we used transcriptions from the natural recordings. First, we assessed the number and type (child-directed, overheard) of utterances received by infants, and who produced the utterance (primary caregiver, another adult, older child). We fitted Bayesian multilevel zero-inflated Poisson models to the data, and found changes across

cohorts in all three measures (see SI Text). Mean number of utterances per hour directed to children decreased from 2007 to 2014 in primary caregivers (99.6 to 76.8 utterances) and, particularly, other children (346.7 to 165.7); number of directed utterances from other adults remained relatively stable (36.86 to 37.79) (Fig. 1, top three graphs). In contrast, mean number of overheard utterances per hour increased slightly in primary caregivers (29.8 to 51.0) and other adults (38.9 to 66.5), and decreased slightly in other children (104.6 to 93.9; Fig. 1, bottom three graphs). For all input types, most utterances originated from other children (68.8% of utterances in cohort 1, 52.8% in cohort 2; Table S7). The results were consistent across all villages (i.e. village-specific intercepts were roughly symmetrical around 0; Fig. S2). Thus, the amount of directed speech children heard in either language, particularly from other children, decreased over the 6-year period; in contrast, the amount of overheard speech children heard changed very little.

Figure 1. Posterior predictive distribution of the mean difference in number of utterances of each type received by the average child from 2007 to 2013, as obtained from the Zero-Inflated Poisson model including "Cohort" as predictor variable and the number of utterances of each type as response variable. These were obtained by averaging from 12000 samples from the posterior distribution (setting the standard deviations for the varying intercepts to 0). From top-left to bottom-right: Directed input from primary caregiver, directed input from adults, directed input from children, overheard input from primary caregiver, overheard input from adults, overheard input from children.

To examine whether the language of input shifted from Yucatec Mayan to Spanish over the 6-year period, we fitted Bayesian logistic mixed models to the proportion of input in Spanish (vs. Mayan) that infants received in child directed and overheard speech. We found significant increases in the proportion of child-directed input that infants received in Spanish between 2007 (Mean=0.21, 90% HPDI: [0.03, 0.47]) and 2014 (Mean=0.67, 90% HPDI: [0.32, 0.93]) (Fig. 2, left graph). In contrast, there were no significant changes in the proportion of overheard speech infants received in Spanish between 2007 (Mean=0.27, 90% HPDI: [0.07,0.55]) and 2014 (Mean=0.17, 90% HDI: [0.03, 0.37]) (Fig. 2, right graph). Thus, caregivers altered the proportion of speech they directed to infants in favor of Spanish over the 6-year period, but the proportion of speech overheard by infants did not change (Table S7). By 2014, caregivers (whether they were primary caregivers, other adults, or children) used Spanish in the speech they actively directed to infants, but primarily used Mayan in the speech overheard by infants.

Figure 2. Posterior means (bars) and 90% HPDI (error bars) for the models predicting the proportion of utterances in Spanish (Number of utterances in Spanish out of the total number of utterances of that type) an average infant (infant with an intercept at 0 for village) is exposed to.

To summarize, infants in cohort 2 generally received less directed input than infants in cohort 1 but, when that input was addressed to them, it typically came in Spanish. Together, these findings indicate that cohort 2 infants were far less likely to hear Mayan in directed speech than cohort 1 infants. Indeed, while infants in cohort 1 heard an average of 397.3 Mayan utterances per hour in directed speech, infants in cohort 2 children heard only an average of 92.51 Mayan utterances.

Why did caregivers decrease the amount of Maya they directed to infants? We turn to interview data gathered in 2019 to address this question. 126 adults from these villages (including all of the primary caregivers from the two cohort samples) were asked if it was more important to learn Maya or Spanish and, as a follow up question, why each language was important to learn. All of them reported that learning Maya and learning Spanish were equally important. Responses to questions about why it is important to learn Maya and Spanish are summarized in Table 1. In general, Spanish was seen as a functional tool important for necessities, such as communicating with doctors or going on shopping trips to nearby Spanish speaking cities. Maya, in contrast, was regarded as important for maintaining social relationships and cultural ties within the village.

Table 1. Answers to questions regarding why each language is important from interviewees. Note that counts do not add up to 126 because responses that did not answer the question, such as ‘Because both are important’ were excluded as they offered no explanatory power.

Question	Answer	Count
**Why is Mayan important/ necessary?**	To communicate in the village, as everyone speaks Mayan	34
	To preserve the language/prevent disappearance	18
	Some people don't understand Spanish	13
	To know where one comes from, it is our cultural heritage/ tradition	9
	One needs it for everything	4
	Learning Mayan allows you to learn how to speak earlier in life	3
	It is pretty	3
	For work (requisite in hotels)	2
	Help old people translate	2
	To speak to one's family	1
	It is more similar to English	1
	To teach one's children	1
	If you don't, people here make fun of you	1
	To go shopping	1
	To be able to work in corn fields ("milpa")	1
	**TOTAL**	**96**
**Why is Spanish important/ necessary?**	For going to the doctor	31
	For visiting cities	30
	To speak/understand visitors	16
	To fully express yourself	7
	To be able to work outside	13
	Now one needs to learn it for everything	6
	One needs it to talk to schoolteachers	6
	It is very popular now	6
	For understanding telenovelas	1
	For bureaucracy	1
	In case you get lost	1
	**TOTAL**	**118**

In order to explore attitudes about the need for directed input in learning language, adults were asked: "Some people believe that language needs to be taught, while others believe that language will just come out on its own, what do you believe? And, as a follow-up, "Is that the same for Maya and Spanish learning?" In response to the first question, 81.9% (N=98) of adults believed that infants needed to be actively instructed in how to speak. This pattern was found in the primary caregivers of infants in both the first (72.3%; N=16) and second (60.9%; N=9) cohorts, with no significant differences between cohorts (χ^2=0.20, df=1, p=0.65). As an example, one mother stated: "Mothers need to speak to their babies for them to learn how to speak because alone they can't. I have a nephew who only learned how to speak

when he was seven because his mother didn't teach him." Interestingly, in response to the second question, 49.4% (N=61) of the interviewed adults believed that infants learnt Spanish and Yucatec Mayan in different ways (Table S10). Specifically, 34.1% (N=43) of interviewees thought Yucatec Mayan was easier to learn than Spanish, sometimes reporting that Spanish required infants to be actively taught, whereas Maya could be learned from overhearing. This pattern raises the possibility that an additional factor responsible for the increase in directed speech in Spanish arose, not from devaluing Mayan, but from the belief that child-directed speech is essential in order to learn Spanish.

To test this hypothesis, we used Bayesian logistic mixed models to assess whether the belief that Spanish was harder to learn than Maya predicted the proportion of input that caregivers directed to their infants in Spanish. We confirmed that children whose primary caregivers thought Spanish was harder to learn were more likely to speak to their children in Spanish both in 2007 (log-odds=1.59, 90% HPDI: [1.06, 2.1]) and 2014 (log-odds=1.1, 90% HPDI: [0.67, 1.56]) (Fig. 3). The proportion of mothers who held the belief that Mayan was easier to learn was not different across cohorts (58.3%, n=7 versus 33.3%, n=3; Fisher's exact text, p=0.21), nor was it related to how fluent they were in Spanish (χ^2=0.47, df=1, p=0.50).

Figure 3. Posterior means (bars) and 90% HPDI (error bars) for the models predicting the proportion of utterances in Spanish (Number of directed utterances in Spanish out of the total number of directed utterances) primary caregivers directed to their infants in Cohort 1 (left) and Cohort 2 (right) according to whether they believed Yucatec Mayan was learnt easier/faster than Spanish or not.

Discussion

Our results show, for the first time, that the quantity, type, source, and language given to Yucatec Mayan infants changed as the community became more connected with urban centers. Infants in cohort 2 received less total directed input than infants in cohort 1 and, when they were directly addressed, most of their input was in Spanish. The net result of these changes was that infants in cohort 2 received far less directed input in the Yucatec Mayan language than infants in cohort 1.

Theorists have proposed that shifts in input away from minority local languages and towards majority languages are due to due to a belief that dominant languages are more advantageous for children to learn than the minority language (8-10). However, in the Yucatec Mayan community considered in this study, reductions in Mayan input did not seem to be due to speakers devaluing the Maya language—our interview data suggest that caregivers saw value in infants learning *both* Maya and Spanish, but for different reasons (32). Spanish was seen as important for achieving pragmatic goals, such as going to town, finding work, or going to the doctor; Mayan was seen as important for social goals, such as

communicating in the village and maintaining the Mayan culture. One marker of the continued importance of Maya was the fact that, across cohorts, most overheard speech was in Mayan throughout the period of change. This pattern indicates that Spanish was not replacing Maya overall, but only in directed speech.

Our study suggests two factors, other than devaluing the local language, that may contribute to the decreasing amounts of input in Maya that infants received. First, in this community, both in the past and currently, most input to infants comes from other children, not adults. We found an overall reduction between cohorts in child-directed input from other children, which might result from infants spending less time with other children. These reductions are unlikely to be due to shifts in family composition following increased market integration (as has been suggested in other small-scale communities e.g. refs. 14,20,33) because we found no evidence that family size changed across cohorts. It is possible, however, that schooling has gained importance over time and that older children are focusing more on schoolwork and less on sibling care. Indeed, in a comparison of childcare patterns in 1992 and 2011 in a very similar Mayan community, Kramer and Veile (34) report that even in the absence of changes in fertility or residence arrangements, the amount of time 7-10 year-old children dedicated to childcare decreased from 12% to a little over 6% (primarily traded off by care from fathers). In future research, we plan to evaluate whether similar changes in childcare practices have occurred in the target communities and, if so, why.

The second factor that may contribute to the decreasing amounts of input in Maya that infants received is caregiver beliefs about the importance of directing speech to children in each of the languages. We found that many caregivers endorsed a belief that Mayan is more ‘naturally’ or ‘easily’ acquired than Spanish; parental effort is therefore better placed at teaching Spanish. This result mirrors findings from other indigenous communities in the Americas. For example, following the introduction of bilingual education programs in a K’iche Mayan community, Choi (30) found that many families did not support teaching K’iche at home or in school. The caregivers thought that the K’iche language was “naturally” learned without schooling, and therefore any attempts to teach it would be a waste of their and their children’s time. However, the same attitude did not apply to learning Spanish; the caregivers believed education to be necessary for Spanish to be learned. Several other researchers working in bilingual indigenous communities have reported similar beliefs (e.g. 8, 35).

Our results demonstrate that these beliefs matter for predicting caregiver behavior. Caregivers who thought that Maya was easier to learn than Spanish were more likely to use Spanish, and less likely to use Maya, when speaking directly to infants. This attitude could be one of the factors that explains local language disappearance. Previous research indicates that speech directed to infants, and not overheard speech, predicts children´s later language competences (36). This result holds even in Mayan communities where overheard speech is prevalent (15). Given this finding, one possibility is that

differential ethnotheries regarding language acquisition could cause majority languages to replace minority ones despite the fact that caregivers place equal value in both languages.

In conclusion, in the Mayan communities that were the focus of this study, increased market integration over the past decade has been associated with an overall decrease in speech addressed to infants, as well as a decrease in the proportion of total input that infants hear in Yucatec Mayan, as this input is replaced with Spanish. Our results suggest that Mayan caregivers are not purposely devaluing the Yucatec Mayan language in favor of Spanish. Rather, caregiver beliefs about the difficulty of learning Spanish vs. Maya, as well as changes in childcare practices, seem to be the primary factors associated with decreases in native-language input directed to children.

Materials and Methods

Ethnographic context

All the data come from four Mayan villages located in the state of Yucatán about 80 miles to the southwest of Cancun (the closest Spanish-speaking urban centre) (SI Text). Traditionally, Mayan families have made their living as subsistence maize farmers. Following the Mexican Revolution, the ejido land tenure system was established, whereby each Mayan village is conceded a plot of land on which to build a house, as well as surrounding country for crops, pasture and woodland (37). Ejido lands (as initially written) could not be owned, inherited, sold, or rented and their dominion resided within the village collective, which distributed them among married males (38).

Recently the Maya have experienced rapid socioeconomic changes due to the growth of lowland towns, creation of new roads, improved transport, greater availability of schools, and increased contact with Mexican and global cultures (38). These changes allowed many individuals, particularly unmarried males, to work for wages in nearby Spanish-speaking urban centers, such as Cancun or Playa del Carmen (39). However, wage jobs are seen as a supplement to agricultural work, needed to increase household productivity in times of need and not as a replacement for it. Nonetheless, in contrast to what many (e.g., 40,41) have regarded as an inevitable and gradual process of acculturation, social and residential structures seem to have remained strikingly stable (25).

Another important change in the region has been the increased access of children from rural communities to education. In most schools (including those from the villages of the present study), textbooks are provided only in Spanish and, although some teachers do use both languages, Mayan is principally employed for classroom management while Spanish is the language of instruction (42).

Naturalistic video recordings

Families that contained a target infant between 16-24 months were video recorded for 60 minutes in their homes in natural interaction. The first cohort of recordings was obtained in 2007-2008 (15) and the second cohort in 2013-2014. In total, 36 recordings of 29 infants were analyzed; 21 recordings in the first cohort (mean age=20.88 months; SD=2.75; 50% female) and 15 in the second (mean age=18.17 months; SD=1.35; 36% female). In both cohorts, an experimenter followed target infants wherever they went (see (43) for details). Family members were instructed to act as they would have had the experimenter not been present.

7 infants from the first cohort had more than one video. However, the recordings for those infants with multiple videos were never less than 6 months apart. Since we used hierarchical models (see Statistical Approach), we are confident that including more than one video per infant has not led to biases in inference.

Transcription and coding of naturalistic recordings

All audible speech from the video recordings was transcribed and divided into utterances, using the same criteria as Shneidman and Goldin-Meadow (15).

Each utterance was classified on the basis of:

a. Who was speaking, primary caregiver (mother), other adult (over 11 years), or other children.
b. Whether it was directed to the infant or overheard by the infant. Speech was considered directed if it was addressed to the infant alone or if it was addressed to a group of individuals that included the infant. All other speech was categorized as overheard. Several cues were used together in order to categorize utterances as directed or overheard: Gaze direction, grammatical marking, utterance content, and proximity to infant.
c. The language in which the utterance was delivered. Utterances were classified as Spanish only if the entire utterance was in Spanish. Utterances where code-switching between languages occurred were not included in this category and neither were utterances in which Spanish words were used as nicknames. Many object words (e.g., *coche*) are borrowed from Spanish even by monolingual Mayan speakers; these words were also not counted as Spanish. Only when there was a commonly used Mayan alternative, yet speakers chose to use the Spanish equivalent, were utterances counted as Spanish.

Interview data

126 adults (female=83; mean age= 35.81, SD=14.87), including all mothers of recorded infants, were interviewed in 2019. During the interviews, adults were asked questions regarding their social, economic and linguistic profiles, as well as those of their family members including all their infants. Interviewees

could choose whether the questionnaire was administered to them in Spanish or Yucatec Mayan. In the former case, the first author conducted the interviews; in the latter case, a local research assistant asked the questions from a previously verified translation. Both the first author and the research assistant were present during all interviews. See SI text for a copy of the questionnaire used and the coding of the responses.

All socioeconomic and demographic variables concerning the recorded infants and their families were obtained from these interviews. To assess the potential causal pathways driving any changes in linguistic socialization at the time of recording, for these analyses, we asked caregivers to answer in retrospective about their family situation at the time of recording. Since some of the mothers' answers concerned the wage labor status of their husbands, we verified them by independently asking their husbands the exact same set of questions. When possible, their answers were further validated with basic demographic records collected at the time of recording.

Informed consent

Informed, written consent was obtained from all adult participants included in the study and from all parents of the recorded children.

Statistical approach

Bayesian inference was used for all statistical analyses. In a Bayesian framework, each model conditions its data on prior probability distributions and uses Monte Carlo sampling methods to generate posterior distributions for its parameters. The priors are the initial probabilities for each possible value of each parameter. Regularizing priors were adopted, which are more conservative than the implied flat priors of non-Bayesian procedures, in order to prevent the model from overfitting the data given the limited sample size (44). Having fit alternative parameterizations for all models, we believe that the results presented below are qualitatively robust to changes in priors.

Even if the four villages were very similar, they differed in the extent to which they were represented. Random intercepts for "village" were included in all models to account for the nested structure of the data and associated clustering (44). Before conducting our analyses, we checked for multicollinearity among predictors using the generalized variance inflation factor (GVIF). All GVIF values fell below the lowest commonly recommended threshold of 4, indicating that our models should not suffer from multicollinearity (45). Our use of regularizing priors should also reduce variance inflation (46).

Parameter estimation was achieved with RStan (47), running three Hamiltonian Monte Carlo chains in parallel until convergence was suggested by a high effective number of samples and R^ estimates of 1.00 (44). This entailed in some cases 5000 samples per chain and in others 10000. In the former case we

used 1000 as warm-up and in the latter 2000. We also visually inspected trace plots of the chains to ensure that they converged to the same target distributions and compared the posterior predictions to the raw data to ensure that the models corresponded to descriptive summaries of the samples.

For model comparisons, we used Widely Applicable Information Criteria (WAIC) which provides an approximation of the out-of-sample deviance that converges to the leave-one-out cross-validation approximation in a large sample (48). Analyses were performed in R 3.5.2 using the brms package (49)(50). We present a complete description and justification of the priors, model specifications, model comparisons and model coefficients in SI Text.

Ethical approval

All procedures involved in this study were reviewed and approved by the Ethics Commission of the University of Cambridge as well as the Ethics Committee of the University of Chicago. They are also in accordance with the 1964 Helsinki declaration and its later amendments or comparable ethical standards.

Acknowledgments

The authors would like to thank Prof Karen Kramer and Prof Robert Foley for helpful comments on the manuscript. The Chay-Cano Family, in particular Cornelio Chay Cano and Maria Leydi Hau Caamal for their assistance in the data collection process. All those that agreed to participate in the study.

Funding

This study was funded by the Department of Archaeology at the University of Cambridge and by a NSF award (BCS-1226113) to Amanda Woodward.

Author Contributions

All authors contributed to the study conception and design. Material preparation and data collection was performed by CPI and LS. Data analysis was carried out by CPI. The first draft of the manuscript was written by CPI and LS and all authors commented on previous versions of the manuscript. All authors read and approved the final manuscript.

References

1. K. D. Harrison, *When Languages Die* (Oxford University Press, 2007).

2. T. Amano, *et al.*, Global distribution and drivers of language extinction risk. *Proc. R. Soc. B Biol. Sci.* **281**, 20141574 (2014).
3. J. Gafaranga, Medium request: Talking language shift into being. *Lang. Soc.* **39**, 241–270 (2010).
4. N. C. Dorian, Language shift in community and individual: The phenomenon of the laggard semi-speaker. *Int. J. Sociol. Lang.* **1980**, 85–94 (1980).
5. N. H. Hornberger, Language policy, language education, language rights: Indigenous, immigrant, and international perspectives. *Lang. Soc.* **27**, 439–458 (1998).
6. A. Kandler, Demography and language competition. *Hum. Biol.* **81**, 181–211 (2009).
7. M. Krauss, The world's languages in crisis. *Language* **68**, 4–10 (1992).
8. C. Rindstedt, K. Aronsson, Growing up monolingual in a bilingual community: The Quichua revitalization paradox. *Lang. Soc.* **31**, 721–742 (2002).
9. A. Veile, M. Martin, L. McAllister, M. Gurven, Modernization is associated with intensive breastfeeding patterns in the Bolivian Amazon. *Soc. Sci. Med.* **100**, 148–158 (2014).
10. D. L. Sam, J. W. Berry, Acculturation: When individuals and groups of different cultural backgrounds meet. *Perspect. Psychol. Sci.* **5**, 472–481 (2010).
11. J. H. Hill, K. C. Hill, *Speaking Mexicano: Dynamics of syncretic language in central Mexico* (University of Arizona Press, 1986).
12. K. Rolstad, Language death in Central Mexico: The decline of Nahuatl and the new bilingual maintenance programs. *Biling. Rev. Rev. Bilingüe*, 3–18 (2001).
13. R. Sear, Beyond the nuclear family: an evolutionary perspective on parenting. *Curr. Opin. Psychol.* **7**, 98–103 (2016).
14. A. Cristia, E. Dupoux, M. Gurven, J. Stieglitz, Child-Directed Speech Is Infrequent in a Forager-Farmer Population: A Time Allocation Study. *Child Dev.* **90**, 759–773 (2019).
15. L. A. Shneidman, S. Goldin-Meadow, Language input and acquisition in a Mayan village: how important is directed speech?: Mayan village. *Dev. Sci.* **15**, 659–673 (2012).
16. P. Vogt, J. D. Mastin, D. M. Schots, Communicative intentions of child-directed speech in three different learning environments: Observations from the Netherlands, and rural and urban Mozambique. *First Lang.* **35**, 341–358 (2015).
17. M. Casillas, P. Brown, S. C. Levinson, Early Language Experience in a Tseltal Mayan Village. *Child Dev.* (2019) https:/doi.org/10.1111/cdev.13349 (September 16, 2020).
18. D. W. Lawson, R. Mace, Parental investment and the optimization of human family size. *Philos. Trans. R. Soc. B Biol. Sci.* **366**, 333–343 (2011).
19. M. K. Shenk, M. C. Towner, H. C. Kress, N. Alam, A model comparison approach shows stronger support for economic models of fertility decline. *Proc. Natl. Acad. Sci.* **110**, 8045–8050 (2013).
20. H. Kaplan, A theory of fertility and parental investment in traditional and modern human societies. *Am. J. Phys. Anthropol. Off. Publ. Am. Assoc. Phys. Anthropol.* **101**, 91–135 (1996).

21. L. Shneidman, A. L. Woodward, Are child-directed interactions the cradle of social learning? *Psychol. Bull.* **142**, 1–17 (2016).
22. M. B. Crago, B. Annahatak, L. Ningiuruvik, Changing Patterns of Language Socialization in Inuit Homes. *Anthropol. Educ. Q.* **24**, 205–223 (1993).
23. B. Rogoff, M. Correa-Chávez, M. N. Cotuc, “A Cultural/Historical View of Schooling in Human Development” in *Developmental Psychology and Social Change*, D. B. Pillemer, S. H. White, Eds. (Cambridge University Press, 2005), pp. 225–263.
24. S. Gaskins, Children’s daily activities in a Mayan village: A culturally grounded description. *Cross-Cult. Res.* **34**, 375–389 (2000).
25. S. Gaskins, From Corn to Cash: Change and Continuity within Mayan Families. *Ethos* **31**, 248–273 (2003).
26. P. Brown, “The cultural organization of attention” in *The Handbook of Language Socialization*, (Wiley Online Library, 2011), pp. 29–55.
27. L. de León, The emergent participant: Interactive patterns in the socialization of Tzotzil (Mayan) infants. *J. Linguist. Anthropol.* **8**, 131–161 (1998).
28. M. Konner, Infancy among the Kalahari desert San. *Cult. Infancy Var. Hum. Exp.*, 287–328 (1977).
29. S. R. Tulkin, J. Kagan, Mother-child interaction in the first year of life. *Child Dev.*, 31–41 (1972).
30. J.-S. Choi, Language Ideology as an Intervening Process in Language Shift: The Case of Bilingual Education in Guatemala. *Asian J. Lat. Am. Stud.* **20**, 55–73 (2013).
31. R. Schacht, H. E. Davis, K. L. Kramer, Patterning of Paternal Investment in Response to Socioecological Change. *Front. Ecol. Evol.* **6** (2018).
32. C. Padilla-Iglesias, R. A. Foley, L. A. Shneidman, Language as a marker of ethnic identity among the Yucatec Maya. *Evol. Hum. Sci.* **2** (2020).
33. S. M. Mattison, R. Sear, Modernizing Evolutionary Anthropology: Introduction to the Special Issue. *Hum. Nat.* **27**, 335–350 (2016).
34. K. L. Kramer, A. Veile, Infant allocare in traditional societies. *Physiol. Behav.* **193**, 117–126 (2018).
35. C. Pye, Quiché Mayan speech to children. *J. Child Lang.* **13**, 85–100 (1986).
36. L. A. Shneidman, M. E. Arroyo, S. C. Levine, S. Goldin-Meadow, What counts as effective input for word learning? *J. Child Lang.* **40**, 672–686 (2013).
37. J. Michnowicz, “Maya-Spanish contact in Yucatán Mexico: Context and sociolinguistic implications” in *New Perspectives on Hispanic Contact Linguistics in the Americas*, S. Sessarego, M. González-Rivera, Eds. (Iberoamericana Vervuert, 2015), pp. 21–42.
38. K. Kramer, *Maya children* (Harvard University Press, 2005).
39. E. Cashdan, K. L. Kramer, H. E. Davis, L. Padilla, R. D. Greaves, Mobility and Navigation among the Yucatec Maya: Sex Differences Reflect Parental Investment, Not Mating Competition. *Hum. Nat.* **27**, 35–50 (2016).

40. M. Gurven, A. V. Jaeggi, C. von Rueden, P. L. Hooper, H. Kaplan, Does Market Integration Buffer Risk, Erode Traditional Sharing Practices and Increase Inequality? A Test among Bolivian Forager-Farmers. *Hum. Ecol.* **43**, 515–530 (2015).
41. J. C. Mijangos-Noh, Los múltiples rostros de un pueblo. 36 (2001).
42. L. de León, "Indigenous Language Policy and Education in Mexico" in *Language Policy and Political Issues in Education*, T. L. McCarty, S. May, Eds. (Springer International Publishing, 2017), pp. 415–433.
43. L. A. Shneidman, *Language input and acquisition in a Mayan village* (The University of Chicago, 2010).
44. R. McElreath, *Statistical rethinking: texts in statistical science* (Boca Raton, FL: CRC Press, 2015).
45. A. F. Zuur, E. N. Ieno, C. S. Elphick, A protocol for data exploration to avoid common statistical problems. *Methods Ecol. Evol.* **1**, 3–14 (2010).
46. C. F. Dormann, *et al.*, Collinearity: a review of methods to deal with it and a simulation study evaluating their performance. *Ecography* **36**, 27–46 (2013).
47. Stan Development Team, Stan modeling language users guide and reference manual. *Tech. Rep.* (2016).
48. A. Gelman, *et al.*, *Bayesian data analysis* (Chapman and Hall/CRC, 2013).
49. R Core Team, *R: A language and environment for statistical computing.* (R Foundation for Statistical Computing, 2018).
50. P.-C. Bürkner, brms: An R package for Bayesian multilevel models using Stan. *J. Stat. Softw.* **80**, 1–28 (2017).

Author Contributions

All authors contributed to the study conception and design. Material preparation and data collection was performed by CPI and LS. Data analysis was carried out by CPI. The first draft of the manuscript was written by CPI and LS and all authors commented on previous versions of the manuscript. All authors read and approved the final manuscript.

Supporting Information

SI Text.

S1 Fig. Spanish level of the primary caregivers of the 14 infants recorded in Cohort 1 (left) and the 15 infants recorded in Cohort 2 (right).

S2 Fig. Marginal posterior distribution of changes in the proportion of utterances in Spanish directed to infants (left), overheard by infants (middle) and received by infants overall (right) taking into consideration variation across the different villages. The solid lines are posterior means and the shaded regions are 80% HPDIs

S1 Table. Descriptive statistics of the primary caregivers of the 14 infants recorded in Cohort 1 and the 15 infants recorded in Cohort 2.

S2 Table. Descriptive statistics of infants recorded in Cohort 1 and Cohort 2.

S3 Table. Descriptive statistics of all female adults interviewed in 2019 (n=83) .

S4 Table. Descriptive statistics of all male adults interviewed in 2019 (n=46).

S5 Table. Nature of wage work of the adult males (n=28) that participated in wage labour

S6 Table. Comparison of Zero-Inflated Poisson models predicting whether the number of utterances heard by infants in one hour had changed across Cohorts with equivalent Poisson models.

S7 Table: Posterior predictive distributions for the changes in proportion of input in Spanish across cohorts for a child from an average village (in this context, average refers to setting the estimates of the standard deviations for the varying intercepts to zero). “ll” refers to “lower limit” and “ul” to upper limit.

S8 Table: Comparison of Poisson models predicting the number of directed utterances received by infants in one hour.

S9 Table: Self-reported beliefs about Spanish acquisition by the 94 adults that were fluent Spanish speakers. The fact that the total is greater than 66 is because some women gave more than one answer to the question.

S10 Table: Beliefs by primary caregivers concerning the ways in which infants learnt the different languages. Counts are the number of females that gave a specific answer to the question of whether they thought infants could acquire competences in Yucatec Mayan in the same way as those in Spanish.

S1 Dataset. Anonymised data.

Figure 3

Click here to access/download;Figure;Figure3.tiff

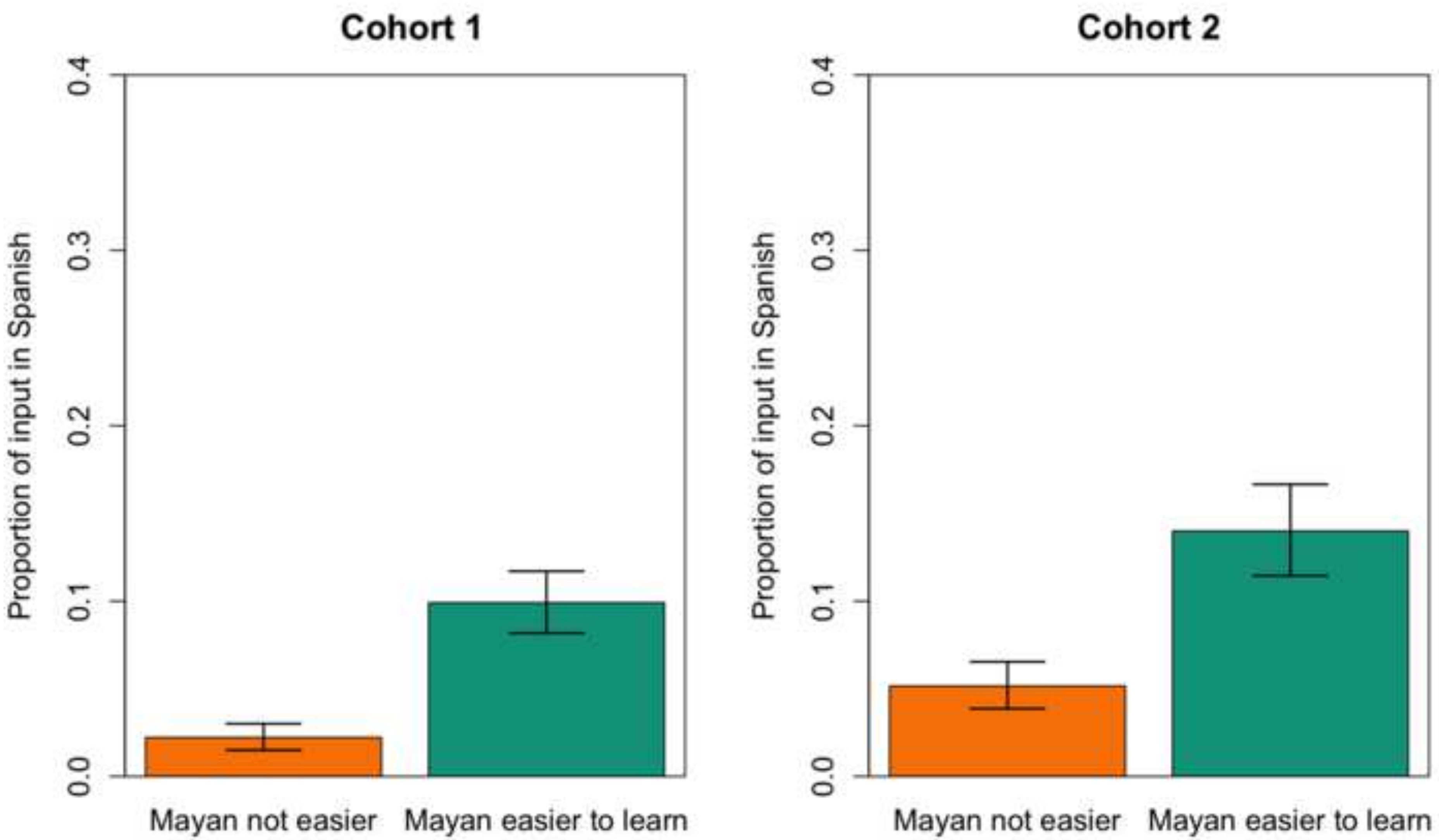

"easy to learn" is a gloss for something else?

Figure 1

Click here to access/download;Figure;Figure1.tiff

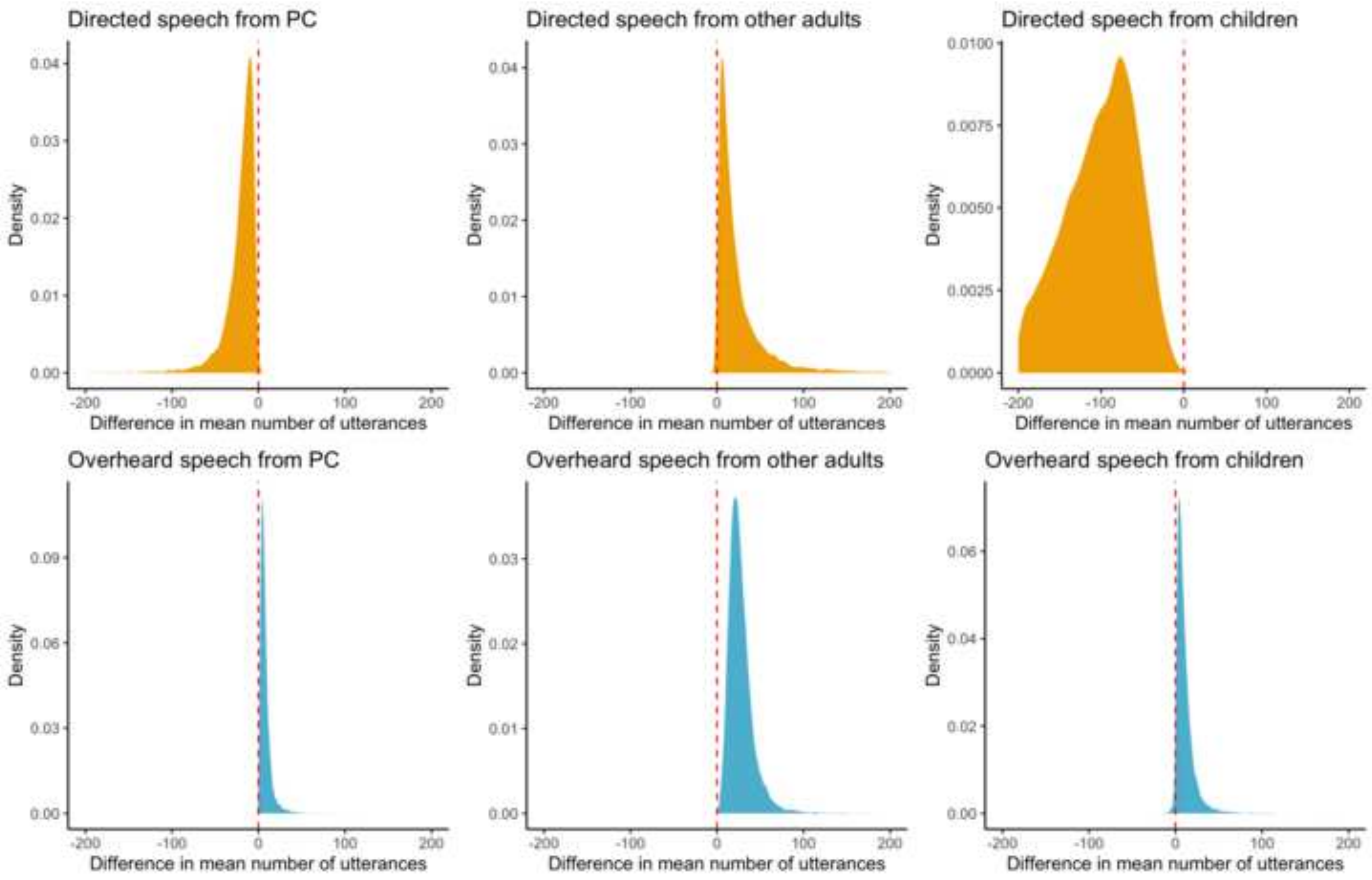

Figure 2

Click here to access/download;Figure;Figure2.tiff

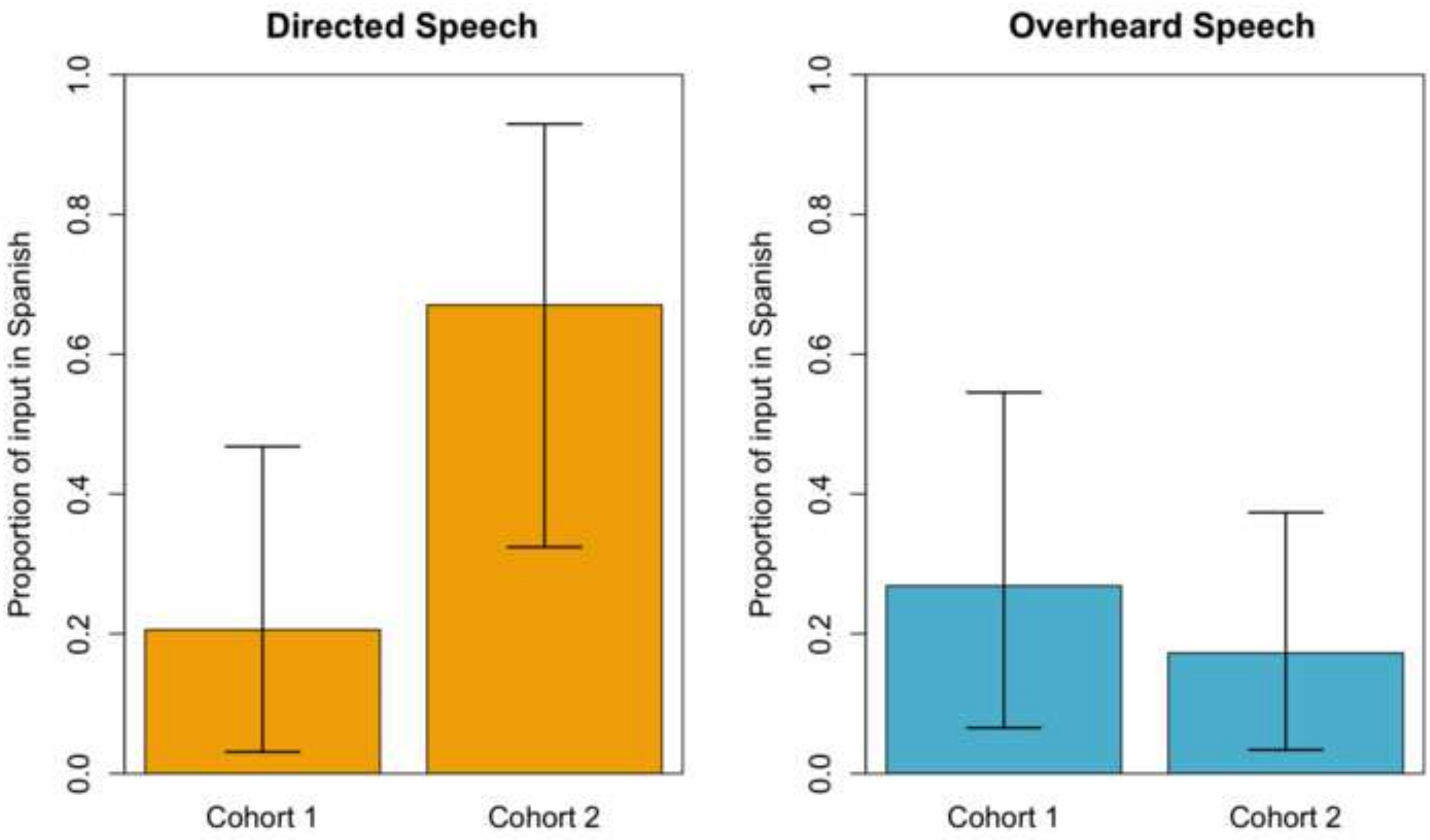

Click here to access/download
Supporting Information
Padilla-Iglesias_PLOS_SM.docx

Dataset

Click here to access/download
Supporting Information
data_anon.csv
