# Supplementary material for: Changing language input following market integration in a Yucatec Mayan community
Source: PLoS One. 2021 Jun 21;16(6):e0252926. doi: 10.1371/journal.pone.0252926 (PMC8216532; doi:10.1371/journal.pone.0252926)
Supplement: Attachment — Submitted filename: PadillaetalReview-1stRevision.pdf [file pone.0252926.s025.pdf]

Padilla et all, “Why local languages disappear.”

First revision

Thanks for giving me the chance to comment on the revised version of this paper, in which the authors assiduously address points raised by several reviewers, virtually all of which I also agree with. However, rather than respond point by point to the authors’ reactions to these points and the changes they have introduced, I will limit myself to the main reservations I expressed about the original paper. In the interests of time (my own), I will again be brief in my summary opinion.

The first thing I note about the revised version is that it has received band aids (sometimes apparently comfortable ones) whereas my own first reading suggested surgery. There are two empirical contributions in the paper: (1) a comparison of the relative weights of Spanish and Maya in CDS in two cohorts of Maya Yucatec learners in comparable village settings between two different periods of language-acquisition studies separated by 6 years, based on coded transcripts of corpora; and (2) a retrospective survey conducted with the mothers of the same children about a range of topics including language attitudes. The study then correlates “patterns” in these two bodies of (in many ways non comparable) data, and attempts to relate the correlations to – well, to quite a number of different things that more or less sit in the background of assumptions about situations like modern Yucatec language and society. The authors now explicitly do NOT claim to draw conclusions about “language shift,” although much of the background literature they site and theories to which they refer ARE about patterns of language shift across the world, and despite the fact that their own abstract BEGINS with a lament about disappearing languages worldwide and ends with “the fate of linguistic diversity.” The authors employ many concepts from the language shift literature, including a contrast between “dominant” or “majority” (or “non-local”) languages and various sorts of opposites (“local,” “lesser used,” “native,” . . .), and about changes in childrearing practices, schooling, literacy, and patterns of work linked to an overall transition, which they adopt with very little critical analysis or comment, that envisions an “integration” from “subsistence-based” (sometimes “small-scale”) to “market” (or “skill-based”) economies, apparently also linked to “majority cultures.”

My original enthusiasm for the paper was linked to empirical finding (1), and the authors’ corrections have at least addressed, if not resolved, most of my doubts about the exact nature of the shift they find over the two-cohort CDS corpora between different kinds of input and the coding they have used to characterize “Maya” as opposed to “Spanish” “input.” (The fact that native speakers have done much if not all of the coding does not assuage my doubts about the categories they have been instructed to code with, but I am happy to let those doubts largely pass for present purposes. Here is just one example, “child directed” and “overheard” can only be mutually exclusive categories if one of them—in this case “child directed”--is based [as it now explicitly is claimed to be] on a disjunctive definition based on a variety different features, from syntax to gaze, and the other—“overheard”—is now a wastebasket remainder category. But surely, in our ordinary life, many utterances explicitly directed at one interlocutor may nonetheless be unabashedly intended to be “overheard” by another, so why is the form decisive rather than the intended effect?) I still believe the empirical result is interesting and important, even without any further speculations about whether or not what parents or other caregivers “believe” about languages is relevant to it, or whether the change is somehow linked to the overall effects of the otherwise largely taken-for-granted process of “market integration” to which the authors refer. It is in this latter context that I agree with other reviewers’ worries about the relative

thinness of the ethnographic background in this study—even with respect to the central ethnographic issue of who takes care of children and how-- but it seems to me that the authors are not headed in the direction of trying to question or otherwise tamper with the chronological socio-historical or economic progression with which they have characterized the Yucatec situation.

I was and remain considerably less convinced by the authors' decision to retain the attitude survey study more or less intact. With respect to my earlier complaints, they write that they still find the survey "a useful tool to understand the patterns we observed with quantitative data" and take comfort from their view that their multiple methods "point to the same conclusions." Given that decision, I have nothing more useful to say on the matter and would prefer to leave to the editors and other reviewers their assessment of publishability.
