# Supplementary material for: Changing language input following market integration in a Yucatec Mayan community
Source: PLoS One. 2021 Jun 21;16(6):e0252926. doi: 10.1371/journal.pone.0252926 (PMC8216532; doi:10.1371/journal.pone.0252926)
Supplement: Attachment — Submitted filename: PloS One take 2.pdf [file pone.0252926.s026.pdf]

Report: *Changing language input following market integration in a Yucatec Mayan community*

I am writing a review of the revision of the manuscript now titled "Changing language input following market integration in a Yucatec Mayan community" (I was reviewer 4 in the authors' reply). While the authors have made some efforts to address concerns about the prior submission, there's still several key places where I find the paper falls short.

On the positive side, the authors have taken care to adjust the terminology and provide additional geographic details about the village that are relevant context for understanding the study here (my original Point C). On this front, my concerns have been addressed well. I also appreciate the authors' efforts to more fully articulate the content of the devaluation hypothesis: "an active attempt of replacing one language with another due to the perception that the majority language is more socially or economically advantageous". This addresses my concern in the sense that I understand much better what the hypothesis is. I do still worry this is a bit of a straw man and would encourage the authors to do more work to support the claim this is a predominant view in the field. Currently, they mostly cite a few fairly obscure papers, (8-10) in the references list, so further support for this would be useful.

Despite these improvements to the paper to me there are still key things missing. At a macro level, the papers hypothesis as I understand it is that a set of social, economic and other changes—"market integration"—produce changes in attitudes and behavior that lead to language shift. The paper shows in detail a shift in behavior between the two periods of data collection, 2007/2008 and 2013/2014.

But as some reviewers including me noted on the original submission, there's very little work even qualitatively to characterize the changes took place between the two dates and essentially none of it happens in the body text of the paper. Without this, it's really hard to know how to interpret the changes in behavior. The SI Text has some such information regarding whether father have milpas, whether they work in wage labor, and more anecdotal changes regarding books and TVs. The main text also includes the information that secondary schooling became available in the communities during this same time period. Given that the primary change in language input during the time period is towards less input from other children, couldn't this plausibly be attributed primarily to the secondary schooling in the community rather than any sort of "market integration"?

The main text in several places suggests a fairly sweeping claim "that the quantity, type, source, and language input to Yucatec Maya infants changed as the

communities became more connected with urban centers, and hence education, wage labour, and market goods became more accessible to their inhabitants", but across the SI text and the main text doesn't do nearly enough to me to establish such larger conclusions. And it's not really clear to me why secondary schooling in the communities constitutes a form of "market integration". As noted for the original submission, I would personally want to see more detailed work (in the main text) characterizing the shifts in the time period in question.

On other side of this picture, the paper's rhetoric throughout is focused on language shift and languages dying, yet in responses to the initial reviews and in the SI text, the authors have been explicit that there in fact has not been a shift in the vitality of the language. I think it is inappropriate to frame the paper in terms of language death given that there is no evidence this is in fact taking place. Yucatán has had widespread bilingualism in many places since colonial times and while it is of course possible these changes are harbinger of language shift, it is far from a given and I would strongly recommend the authors rethink this framing. I find the mindset of assuming that minority languages are doomed to extinction harmful and I really wish the paper tried to avoid this mindset given the lack of empirical support for any shift in progress.

To summarize, I think this paper presents an interesting set of core findings about language attitudes and changes in language input. However, it consistently presents itself as an assessment of *how* socioeconomic changes produce language shift and loss of language diversity while providing cursory evidence of socioeconomic change and no evidence of language shift.
