# Supplementary material for: Changing language input following market integration in a Yucatec Mayan community
Source: PLoS One. 2021 Jun 21;16(6):e0252926. doi: 10.1371/journal.pone.0252926 (PMC8216532; doi:10.1371/journal.pone.0252926)
Supplement: Attachment — Submitted filename: Response to reviewers.docx [file pone.0252926.s024.docx]

Dear Dr Peña Garay

We thank you very much for editing our manuscript and giving us the opportunity to revise it. We believe we have addressed all questions raised by the reviewers and hope you are satisfied with the replies and the new manuscript.

First of all, regarding the issues related to the formatting of the manuscript, we believe it now meets PLOS ONE’s style requirements (we have edited the ethics statement and put it within the Methods section).

Secondly, in the light of the reviewers more specific comments, we have amended the text and supplementary materials as shown below.

Reviewer 1

*This article reports changes in infant-directed speech that occurred between 2007 and 2013 in four Yucatec Mayan villages with populations between 400 and 600 inhabitants. The villages are close enough to the major urban centers of Cancun and Playa del Carmen to make it practical for men to travel to these towns for employment. The Yucatan Peninsula has seen major development in the international and national tourist economy for many decades, which attracts more villagers to these urban centers. This study offers a unique portrait of the impact that these changes have made to the language environment of Yucatec Mayan infants and thus to the forces responsible for language shift in the Yucatan.

The paper reports important research that contributes unique data to the study of language shift. Nevertheless, there are several issues the authors should address before their paper is published.*

We thank the reviewer for highlighting the importance of these topics and for their thoughtful and detailed review. We address each point in turn below.

*The title of the study suggests a causal connection between market integration and language change, and the first sentence of the Discussion (p. 8) states “Our results show, for the first time, that the quantity, type, source, and language given to Yucatec Mayan infants changed as the community became more connected with urban centers.” There are two difficulties with this claim. First, the paper does not supply details about the market integration taking place in the region between 2007 and 2013.* *Were there specific developments that took place or do the changes in input reflect prior changes or other factors?*

Thank you very much for raising this issue. As it is the case with studies that inevitably rely on observational data in natural environments, there are multiple social and demographic changes that have taken place over the 7-8 year period that our study spans. We use the umbrella term “market integration” to refer to all those inter-dependent processes that have taken place as a result of increased connectedness with urban centres, access to education in Spanish, availability of market goods and participation in wage labour. Regarding the aforementioned variables, please refer to the first paragraph of the results section (page 4) as well as S1 Table for quantitative reports on changes across cohorts regarding wage labour participation and educational access. In addition, we have edited and expanded the SI Text to provide additional information on the market integration processes taking place in the villages in our study.

*Second, the paper does not report any effects that changes in the input might have had on the children’s language production. Did the children continue acquiring Maya?*

Thank you for raising this concern. We would like to clarify that while we do believe that decreases in language input will lead to changes in children’s language production in the long term (given prior research that indicates that speech directed to children uniquely relates to later language competencies), we are not making a direct causal claim regarding this issue in this paper. Rather, we are evaluating how changes in socio-economic and demographic variables impact caregivers’ strategic *decision making* in teaching language to their children, as well as children’s opportunities to acquire languages. We believe that these changes could be one mechanism that potentially explain eventual language loss in Maya. We understand now how our title and wording could be confusing on this point and have changed both to reflect the specific claim we are trying to make (one between market integration and language input). The paper is now titled ““Changing language input following market Integration in a Yucatec Maya Community” and we changed the first sentence in the discussion to read *“Our results show, for the first time, that the quantity, type, source, and language input heard by Yucatec Maya infants changed as the communities became more connected with urban centers, and hence education, wage labour and market goods became more accessible to their inhabitants.*

In regards to the question: *Did the children continue acquiring Maya?*, as shown in the main text, the majority of children’s total input was in Yucatec Mayan. We did ask families that were interviewed in 2019 (when children from the first cohort were 13-14 years old and those from the second cohort were 6-7 years old) about language competences in Yucatec Mayan and Spanish at that time. In total, 80% (n=8) of kids in Cohort 1 were Spanish speakers, compared to 63.6% (n=7) of those in Cohort 2 (perhaps reflecting that fact that Cohort 1 children were older at this time and had been in school longer). All children also spoke Yucatec Mayan. The reason why we do not report these findings is because we did not quantitatively assess children’s proficiency on each of the languages and therefore we are uncertain of possible consequences for different aspects of language production due to the observed differences in child directed speech patterns. In our current work we are performing comprehension and production tests on both languages in children of different ages in the villages, and hope to use these data to follow up on these very interesting questions.

*The main finding of the study is that “the amount of directed speech children heard in either language, particularly from other children, decreased over the 6-year period” (p. 5). This result is at odds with explanations of language shift in that the Yucatec communities aren’t shifting languages so much as refraining from talking to their children.*

Thank you for pointing this out to us. We see now that our language on this point was confusing as we were trying to convey that there was overall less directed speech (when looking at the total input in both languages) and not that directed utterances decreased in each language. In fact, changes in directed input were driven (fully) by decreases in input in Yucatec Maya. Input in Spanish was very rare in the first cohort (mean of 7 utterances per hour) and increased in cohort 2 (80 utterances per hour). Thus, two changes were taking place simultaneously: a reduction in overall speech directed to children and a shift in the language that was used (from Maya to Spanish) in instances where speech was directed to children. In the revised manuscript we changed our language (removing the “in either language”) to clarify this point and we include a table (Table S7) with the mean number of utterances from each interlocutor received by children in the different types of speech across cohorts (see Figure 1 in the main text for the entire posterior distribution of these models), and then Table S8 for an indication of the percentage of those types of utterances that were in Spanish.

*The authors do not offer an explanation for the reduction in speech to children. Does the reduced speech to children reflect the forces of market integration or the effect of other changes taking place in the Yucatan?*

We agree that this is an interesting finding, that deserves further research. While we do not know why speech input showed overall decreases, we hypothesize that it is due to children spending less time with other children (who are their primary interlocutors). We detail this on page 9 (third paragraph of the Discussion section) where we say:

We found an overall reduction between cohorts in child-directed input from other children, which might result from infants spending less time with other children. These reductions are unlikely to be due to shifts in family composition following increased market integration (as has been suggested in other small-scale communities e.g. refs. 14,20,33) because we found no evidence that family size changed across cohorts (S1 and S2 Tables). It is possible, however, that schooling has gained importance over time and that older children are focusing more on schoolwork and less on sibling care. Indeed, in a comparison of childcare patterns in 1992 and 2011 in a very similar Mayan community, Kramer and Veile (34) report that even in the absence of changes in fertility or residence arrangements, the amount of time 7-10 year-old children dedicated to childcare decreased from 12% to a little over 6% (primarily traded off by care from fathers). In future research, we plan to evaluate whether similar changes in childcare practices have occurred in the target communities and, if so, why.

*Restricting attention to the speech directed to the children, the authors report an increase in the proportion of Spanish utterances directed to the children (p. 5). The overheard speech did not exhibit a similar shift, and thus the result leads to the question of what factor would explain this shift? The authors phrase the issue (p. 6) as one of decreased use of Maya rather than an overall decrease in their speech to infants. This perspective is at odds with the statement (p. 5) that the parents also decreased their use of Spanish.*

Thank you for this remark. Again, we apologize for the confusing wording and would like to clarify that parents did not decrease their use of Spanish across the two cohorts (as Spanish was very rarely used in the first cohort). For our analysis looking at the proportion of utterances in Spanish vs. Maya we used logistic regressions, we estimated the number of directed utterances in Spanish out of the total number of directed utterances received by children. In other words, it is the relative amount of Spanish vs Maya in directed input that we are quantifying: Given that a caregiver directs speech to children, how likely is it that such speech is in Spanish?. Hence, overall changes in the total number of directed utterances received by children should not necessarily alter this proportion. This is why we report the additional finding that, in fact, not only the relative amount but also the total number of utterances heard by infants in directed speech in Yucatec Maya also decreased across cohorts. See the modified version of the main text (bottom of page 5 and first paragraph of page 6) where we have tried to clarify this.

*The ethnographic description of the households and villages is too thin to assess the impact of other factors and thus evaluate the significance of the four factors identified in the study. One omitted factor is the introduction of satellite tv to Mexican villages. Many remote Mexican villages now have access to cable tv, and households with cable tv watch it much of the day, but especially in the evenings. Television dissolves the social fabric by subtracting from the time that families previously spent gossiping in the local language with friends and family. The paper should state the number of homes that had tv in the two cohorts. The introduction of cable tv offers a direct tie to the forces of market integration in that the men who work in the tourist sector would see television as a marker of social status and want to take television back to their own village. Men with cable tvs would have more status than men who just worked in the milpa and so television would take over the village. This hypothesis recognizes a pathway for importing urban behavior to the home village leading to a shift in culture that underpins the shift in language. A factor like “the general devaluing of the native language” does not identify the source of such a devaluing and how it becomes general.*

Thank you for raising this point. We have now added more information regarding the households and villages to the supporting information (page 1) including a section on the presence of televisions. Indeed, whilst in 2007 most households did not have televisions, in 2013 the opposite was true (unfortunately we do not have data on which specific individual households had or did not have televisions). Nonetheless, from our experience, rather than subtracting from the time families spent gossiping, it is often just played in the background whilst families are talking or whilst female heads of households are carrying out domestic duties. Nonetheless, we see the introduction of televisions as another element of market integration, as these are purchased in market towns with cash income.

We agree with the reviewer that in case that we observed a “devaluation of the native language”, further exploration onto precisely what features of market integration result in locals devaluing their own native tongues would be required. Yet, we wish to stress that whilst we have observed a decrease in the use of Yucatec Maya by caregivers when directly addressing young children—this seemed to be a strategic decision specific to speech directed to infants and not a broader devaluation of Maya (as all adults believed competences in Yucatec Maya to be equally valuable as those in Spanish) and there was no reduction in the use of Yucatec Maya overall (as would be the case if for example tv time had substituted for family gossip time). Yucatec Maya is still the preferred language in the village as evidenced by its continued prominence in overheard speech. When adults or older children were talking to each other, they almost exclusively did so in Maya; Spanish was only used in speech directed to infants.

*The paper mentions in passing a more sinister factor in the absence of Yucatec teachers and language materials in the village schools (p. 10; Supplementary text, p. 2). The possibility has to be admitted that the absence of such support is a deliberate policy on the part of the national government to suppress the use of local languages. Many indigenous teachers are sent to teach in urban areas, while teachers from urban areas are sent to teach in rural schools. Teachers from outside the village are more likely to model intolerant attitudes towards the local “dialects”. It would be helpful if the paper supplied some information about where the village teachers come from and their attitudes towards Yucatec.*

In the revised manuscript we have provided more information regarding the teachers in the villages in the supporting information (page 1). We clarify that “Due to the remoteness and transportation costs associated with travel to the villages, there is a high turnover of teachers in the village schools (with many teachers staying only one or two years to obtain the necessary seniority for a more convenient urban placement). Schoolteachers were and are sometimes speakers of Yucatec Maya but more often were/are not.” We do not have details regarding individual teacher attitudes towards Maya.

*The authors should clarify the following points:*

*Participants: The paper should state the number of participants, if any, who were included in both cohorts. Including the same families in both studies might lead to an observer effect and possibly depress the number of utterances they produced. How many of the original families participated in the interviews?*

All families participated in the interviews except for those of 5 of the recorded children (see modified *Interview data* section in Methods where we have clarified this). In addition, we have also now specified in the main manuscript (*Naturalistic video recordings* section) that only one woman had children recorded in both cohorts. We note that we minimized this potential problem; by using hierarchical models (see *Statistical analyses*), we rule out the possibility of particular families driving the overall trend.

*The utterances were classified by speaker: “primary caregiver (mother), other adult (over 11 years), or other children” (p. 11), but the paper only provides information about the primary caregivers (S1 Table) and the target infants (p. 11; S2 Table). It should provide similar tables for the other speakers.*

Unfortunately, it is not possible for us to include such a table because, due to the naturalistic nature of the recordings and the characteristics of village life, household members did not always correspond to the “speakers” in the videos. Because Maya nuclear households are typically situated on plots of land adjacent to neighbouring family structures, and because subjects in the video were permitted to go anywhere they pleased, it was very common for speakers to show up in videos who were not household members (e.g., visiting cousins, shopkeepers the children visited, etc.). As such, it was not possible for us to keep track of who each speaker was in the video if they were not the primary caregiver (they were simply identified as either being a child or adult in coding). In the revised manuscript on page 11, we clarify that it was not uncommon for members outside the household to appear in the videos.

*All of the participant information should be presented in one place.*

Whilst we present the information regarding the composition of our subject sample in the “Materials and Methods” section of the main text, we believe the precise descriptive statistics regarding the demographic characteristics of such sample are best placed in the supplementary material.

*In the discussion on p. 9 the paper states it is possible “that older children are focusing more on schoolwork and less on sibling care.” This should be obvious from the number of older children present in the two cohorts.*

As shown in Tables S2 and S3, the number of older children in families did not change across cohorts. However, it is possible that even when children are present in the household they are less likely to focus on childcare (and thus speak less to and around other children in our recordings). While a more fine-grained analysis of the allocation of time by siblings exceeds the scope of the present study, it is a topic we are exploring in future work.

*Language samples: The families were “recorded for 60 minutes in their homes in natural interaction” (p. 11). The authors should add more information about these recording sessions such as whether they were made inside or outside the house, and the proportion of time spent looking at books. Changes in the use of books between the two cohorts might account for the changes in the input.*

Thank you for this suggestion. In the revised manuscript we provide more information on page 11 regarding the recordings. We say:

“ Family members were instructed to act as they would have had the experimenter not been present and were told that the infant was permitted to go anywhere that they normally would be permitted to go. Recordings varied in their location (inside and outside) depending on the actions of the infant and family members. Infants and family members typically engaged in activities like exploration outside, visits to neighboring households, eating, playing, grooming, etc. It was not uncommon for persons outside the nuclear family to appear in the video recordings (nearby extended family, shopkeepers etc.).”

Unfortunately, we do not have a quantification of the time spent looking at books; however, based on our observations, looking at books was extremely rare in both cohorts, and we believe unlikely to account for the observed trends.

*Maya versus Spanish: “Utterances were classified as Spanish only if the entire utterance was in Spanish. Utterances where code-switching between languages occurred were not included in this category and neither were utterances in which Spanish words were used as nicknames” (p. 11). It is unclear whether utterances with code-switching were excluded entirely or coded as Yucatec. The discussion of Figure 3 (p. 8) defines the proportion of Spanish utterances as the “Number of directed utterances in Spanish out of the total number of directed utterances” so it appears that the authors coded the utterances as either entirely Spanish or other. This interpretation is at odds with the statement “while infants in cohort 1 heard an average of 397.3 Mayan utterances per hour in directed speech, infants in cohort 2 children heard only an average of 92.51 Mayan utterances” (p. 6). On p. 5 the paper states “To examine whether the language of input shifted from Yucatec Mayan to Spanish over the 6-year period, we fitted Bayesian logistic mixed models to the proportion of input in Spanish (vs. Mayan) that infants received in child directed and overheard speech.” The paper should clarify what was counted as a Mayan utterance, and address the question of whether the code-switched utterances were excluded from both the Mayan and Spanish counts.*

*Without knowing how the proportion of code-switched utterances changed between the two cohorts, it’s impossible to tell if the number of Spanish utterances changed significantly. The paper should report the number of code-switched utterances. The code-switched utterances should have been analyzed for the number of utterances with verbs in Spanish or Yucatec. In fact, the proportion of utterances containing Yucatec or Spanish verbs would be a more sensitive measure of language shift than the proportion of Spanish utterances because it would obviate the need to decide whether or not individual words were Spanish. An increase in the number of utterances consisting entirely of Spanish nouns and noun phrases (un carro, chicle, galleta) would not suggest a significant shift in language.*

Thank you for pointing out our need to clarify this. In the revised manuscript on page 11 we now provide more details regarding utterance classification:

Utterances were classified as Spanish, Maya, or, if speakers switched between languages mid-utterance: Code-switching. Many object words (e.g., *coche*), nicknames (e.g., *Gordo*), kinship terms (e.g., *tía*), numbers greater than three (e.g., *cinco*), and calendar terms (e.g., *enero*) are borrowed from Spanish even by monolingual Maya speakers; if speakers used these loan words (either embedded in a Maya utterance or alone), the utterance was classified as Maya (and not as a code-switch or Spanish utterance). Only when there was a commonly used Maya alternative, yet speakers chose to use the Spanish equivalent, were words and utterances classified as Spanish (for example if a speaker said “nariz” for “nose” instead of the Maya word “ni”).

In the revised results section (page 5) we also report the number of code-switched utterances and we clarify that we excluded utterances that were coded as being code switches (due to mid utterance code switching being extremely rare):

Code-switching within utterances was extremely rare (mean = 1.6 utterances per hour, SD= 2.08 and mean = 3.5 utterances per hour, SD = 7.09 in cohorts 1 and 2 respectively; out of an average of 464 utterances heard per hour) and did not vary across cohorts (t = -1.01, df = 14.59, P = 0.33). We therefore excluded them from these analyses.

Indeed, the Bayesian GLMMs reported in the paper were fitted with the number of Spanish utterances out of total number of utterances in Spanish + the total number of utterances in Yucatec Maya. However, due to the rarity of code-switched utterances, running the same models with instead the total number of Spanish utterances out of total number of utterances as response variable did not change any of the coefficients.

*Villages: The paper states that the recordings were made in four villages (p. 10), and that the villages were very similar (p. 12). The Supplementary text adds information about the populations (p. 2). All information about the villages should be presented in one place in the paper. The paper should provide more details about the relative levels of roads and schools in each village, the proportion of the men who participated in wage labor from each village, and the number of participants from each village in each cohort. These details would help readers understand the degree to which the changes took place regionally rather than principally in one or two villages.*

Thank you for this suggestion. In the revised manuscript we provide these additional details in the supplemental materials on page 1. All villages have access to schools and are connected by the same road to each other. The proportion of male heads of households that participated in wage labour did not vary across villages. Please see new section in SI text where we have now included the percentage of wage labourers in each of the villages alongside a chi-square test of independence showing the lack of an association between village of origin and tendency to participate in wage labour.

Furthermore, since we included village as random effect in our models, this allows us to be confident that the observed trends are consistent across the four villages rather than driven by changes in a single one. See also S2 Figure with marginal posterior distribution across villages, which also confirms this claim.

Regarding the presentation of the information regarding the villages, we have reserved the main text for a general overview of the setting where the villages were located, and placed the specific information on each of the villages in the SI Text (please see the modified version).

*This is a solid investigation and I encourage the authors to add further details to their paper.*

We thank the reviewer for their enthusiasm and hope we have provided the necessary details in our revision.

Reviewer 2

*Reviewer #2: The paper examines factors involved in language shift by analyzing interview data, family histories, and transcripts of natural data (to assess input) over a 6-year period in 4 Yucatec Mayan villages where language shift to Spanish is in process. The analysis supports the central argument, that Maya language input has decreased due to social changes that are not explicitly related to language but rather to other social changes (e.g. increased numbers of Mayans working for wages in Spanish-speaking cities, decrease in the availability of older siblings to care for young children because they now attend formal schooling at higher grades than previously) which directly affect language input. The paper is clearly researched and argued; the data are clearly presented and all analyses explained thoroughly and well-documented. The supplementary materials provide robust data supporting changes in education, in the caretakers, and in the language used by them with the children, and in the fathers' earning sources. (I did not see data supporting an increase in market goods or directly linking an increase in wage labor to Spanish, maybe I missed it.)*

Sorry for the lack of clarity on this matter. Please refer to the SI text, which has now been modified to explicitly described the changes across cohorts regarding market integration as well as Figure S1 (a comparison of the Spanish level of caregivers across cohorts), Table S1 (a comparison of age at first birth and years in education of caregivers of each of the cohorts). These changes are also reported in the first paragraph of the results section (page 4).

*The finding that Mayan communities find Spanish more practical than Yucatec Maya is not surprising or novel, given similar findings in most shifting ecologies; the novelty is perhaps a bit overstated in the paper. The significant contribution of this paper is the very rigorous way it has determined the attitudes and beliefs of the speaker communities and the actual input the children, in both directed and overheard speech. It is also provides a model for future work in other communities to understand the causes of shift.*

Thank you for this comment. We agree with the reviewer that finding that participants found Spanish to be practical is not novel or surprising given previous research. Our intention was not to stress the novelty of this finding but rather the novelty of the other contributions that the paper makes (that Maya was not devalued as indexed both by participant report and behavior in speech not directed to children, that beliefs about ease of acquisition related input, etc.). We hope that the revised manuscript is clearer in highlighting these aspects of the study.

*The relevant social and demographic changes discussed here are encapsulated by the authors with the term market integration, and I would recommend that the authors define what they mean by market integration in this paper early on, as it is used in a non-standard way, OR change the terminology to specify increased contact with urban centers (which is specified on p. 8) OR increased education/wage labor/market goods (p. 4). The discussion of the relevant urban contact is provided under Materials & Methods on p. 16 of the manuscript. A sentence definining what is meant in the beginning of the paper would be welcome.*

Thank you very much for this remark. We have now altered the definition in page 8 to match that on page 4 and, in so doing, provide a single framework of what we mean by market integration. We have also included a definition of market integration on the second paragraph of the introduction.

*I would further note that it is odd to think about periods of market integration:
"during periods of market integration, both parents and older siblings of young children in small-scale communities are more likely than previous generations to have had access to formal schooling provided by the majority community" (p. 2). Market integration is not usually conceptualised as a transitory state. Rather, markets usually start out not integrated and then improvements in road conditions or whatever barriers there were to integration would make them better integrated. So here my sense is that the intent of the authors is a before/after comparison, and the wording could be tweaked.*

Thanks for this, we have now changed this sentence to “during the process of market integration”, to indeed indicate that it is a directional and non-cyclic process.

Reviewer 3

*The paper presents one very significant finding, based on a comparison of two different cohorts of Maya Yucatec speakers in two different periods of language-acquisition studies separated by 6 years. I found this main empirical finding striking and important (although not without some questions). I think the work should be published if only to disseminate this well founded and potentially useful result. It is, in brief, that over the very short 6 year span, there was significant change in the sort of direct language input infant Yucatec Maya language learners received. It went from being largely in Maya to almost entirely in Spanish (with some empirical caveats I will shortly mention).*

Thank you very much for the detailed feedback. Please see below the response to your specific comments in the attached document:

*I have a few questions about even this result, however, not resolved by the supplementary materials, which provide almost no information about these video corpora and their “transcriptions.” A number of at least potentially problematic “coding” issues can thus not be resolved or even addressed. One is the nature of the distinction between child-directed and “overheard” language, or about determining who is saying what to whom in the video recordings, especially if working just from the transcripts rather than the original videos. More problematic, although more or less explicitly addressed, is the definition of what is “Mayan” and what is “Spanish.” What is counted is, apparently, “utterances,” and the authors say they count as Spanish only what is “all in Spanish,” despite the ubiquitous presence in modern Yucatec of lots of Spanish loans and stock phrases, at various levels of historical assimilation to Maya phonology. It is, thus, a bit unclear what counts as one language or another, as there is considerable bivalency. At least some examples of how these counts were conducted would be reassuring, although the rather stark implied filter [Spanish-only, vs. the rest] suggests that the results must be largely as claimed.)*

The people that transcribed the videos were from the villages in question and hence knew who the children’s mothers were, but nonetheless the coding was done not only using the transcriptions, but also the videos by the first and last authors (who also knew who the children’s mothers were). We have now clarified this on page 12. In addition, see the new paragraph in the Methods section explaining the coding of utterances into Spanish or Maya as well as the response to reviewer 1 regarding this same issue.

*I find the analysis, methodology, and conclusions of the main argument of the paper, and its associated interview data, considerably less convincing. The paper frames itself within a tradition that places high importance on CDS for language acquisition outcomes, with some caveats about where this CDS comes from. (Is it addressed to infants by adults, or does it originate with other children, who may often be primary caregivers?).It then summarizes several extant hypotheses about what might account for a language shift in such talk, discounting major demographic and economic shifts in the community in question. Is childcare changing so as to change the language input infants receive? Does schooling in a “majority” language increase over the whole community? Are there changes in parents’ ideas about the importance of native languages vs. “non-local” ones, or about the relative difficulty of acquiring them?It then goes on to offer results of what I guess can be characterized as an attitude survey (although more is involved than an assessment of language attitudes), using a different methods to address these various proposals about what might reasonably account for rapid language shift in the language of CDS in these communities. As any anthropologist worth her salt will attest, attitude surveys actually often tell you very little about what people actually DO. This is perhaps especially true of surveys asking people about what they did and thought 5 or even 10 years after they did whatever it was. Moreover, a question like “is it ‘more important to learn Maya or Spanish’” is far from neutral, depending one who’s asking, who’s listening, and what you had for breakfast that morning.*

We thank the reviewer for raising these points regarding the issues that can arise via the use of self-report. We do not believe, however, that these issues preclude the survey from being a useful tool to understand the patterns we observed with quantitative data. To the contrary, we believe that one of the strengths of our study is that multiple methods point to the same conclusions. For example, subjects reported that Maya was important for navigating social relationships in the village and, indeed, we can see evidence of this in the quantitative data which suggest that Maya is quite dominate in overheard speech (across time). In addition, if responses to the survey questions were random, we would not expect the survey to hold explanatory power over subjects’ behavior in video recordings that were taken *before* they participated in the survey. Yet, subjects who reported that they thought Spanish was harder to learn than Maya had previously directed more Spanish to children, suggesting, we think, that these reported attitudes are relatively stable and meaningful for predicting “what people actually do”.

In terms of subjects’ accuracy in thinking back in time, we would like to clarify that we did not ask retrospective questions about beliefs or attitudes (we only asked subjects to consider their attitude at the time of survey). The only retrospective questions that were asked had to do with demographic factors (e.g., how many children did you have in 20XX). While these types of questions may have had some inaccuracies, we believe that generally subjects could accurately report demographic facts even after a delay. See the slight clarification we have made regarding this matter on page 12, as well as the description of how we validated the data by means of asking the two heads of household (female and male) the same questions, as well as triangulating, when possible, such answers to basic demographic data collected at the time of recording.

*Results are even more dubious if, as the table says, “both are important” answers are eliminated as “offering no explanatory power.” The latter clause is certainly true, but such results are not irrelevant to evaluating the eliciting instrument!) Since all of the presented results (but none on the data) are in English, it becomes even more difficult for readers toe valuate the success and reliability of the collected statistics here. (Here is one example: bottom, p. 7, and interviewee is quoted as mentioning “a nephew who only learned how to speak” at age 7—but we have no idea what this means? Mute until 7? Spanish speaking only until 7 [which is what this reader assumed, by contextual inference]. There is not way to evaluate these interviews without something more substantial to go on, and no transcripts are offered of the interviews, to pick out the Mayan from the Spanish, etc.—if any of this matters [which, to the authors, it does not seem to].)*

Sorry about the lack of clarity in this. First of all, we did not eliminate answers of people who believed both languages were important. What we meant in the table is that we did not include in *that specific table* explanations to the belief that both languages were important that simply re-stated that same fact. We realize that this is ambiguous so have removed it from the table footnote.

Regarding the transcriptions from the interviews, given that there were 126 interviewees, and that interviews were around 35 minutes per participant, it would have been challenging to include transcriptions of all of them. We *have* included the coding scheme in the SI text and attached the data as supplementary material. In addition, now we have added to the supplementary information a Yucatec Maya version of the questionnaire used.

*Another conclusion, left very dubious, involves the 4th hypothesis about whether one language ascends because it is thought to be ”harder to learn.” “We confirmed that children whose primary caregivers thought Spanish was harder to learn were more likely to speak to their children in Spanish.” (p. 8). There is first ambiguity about whether “primary caregives” and “mothers” (mentioned in the next line) are the same people. But I am at a loss to know exactly what the questions asked were, or how the answers were evaluated, in the contexts in which they were answered. The claim is repeated in the discussion (last para. P. 9), but no statistics or charts (that I can see) are offered to show this.*

Please refer to the methods section, where we specify that children’s primary caregivers in the present setting were their mothers. Parents’ beliefs regarding whether Maya was easier to learn than Spanish were obtained from their answers to the questions in the last paragraph of page 7. “Some people believe that language needs to be taught, while others believe that language will just come out on its own, what do you believe? And, as a follow-up, “Is that the same for Maya and Spanish learning?”. See also the full questionnaire in Yucatec Maya in the SI Text, as well as the modified version S11 Table for a classification of the answers people gave to such question, and how they were coded in this respect. The statistics relating parental beliefs regarding the languages’ ease of acquisition and their tendency to direct input in Spanish to their children are reported in the second paragraph of page 8, as well as on Figure 3. The precise specifications of the models used to obtain them can be found on page 12 of the supporting information file.

*The discussion the authors try to address here is important, and I myself have little doub that one of the main changes in the period the authors consider has to do with access to education in rural Mexico, and more specifically to access to education for girls, who otherwise would have been primary caregivers (in the normal sense—i.e., not just mothers) for many infants. But access to education is not the same as getting educated, and one needs to look in more detail at the facts on the ground, not just people tell you about them, before any reasonable conclusions can be drawn.*

We recognize nuances surrounding some of the variables we have utilized. However, we believe to have captured the large majority of changes in access to education by assessing: 1) availability of schools in the local villages (SI Text; page 4 of the main text), 2) changes in number of years spent in education by caregivers (S1 table, Page 4 of the main text).

*I would accordingly suggest that the authors reframe the paper so as to foreground the strength and importance of their main empirical result, and temper their enthusiasm for their much more speculative claims about what might account for the change in “direct input,” and how it might relate to various factors they suggest might, in general, account for rapid language shift. This latter question is itself of great importance, both to understanding and to issues about language shift and the survival of minority languages more generally, but the arguments the authors make in this regard are considerably more tenuous and less generalizable than the main empirical finding.*

We are not certain of the fact that we are observing a process of language shift. We thus prefer to remain agnostic regarding whether the reported results are symptomatic of a generalized process of language shift, given that most of the language input that children get is still in Yucatec Maya. We wish to focus the paper on changes in caregivers’ decision-making when speaking to children and draw attention to some of the factors potentially driving such changes. In order to assess whether the pattern observed in CDS translates into a shift in the linguistic repertoire of people, the implications of this pattern for children’s subsequent language competences would have to be evaluated (this is an extremely important question which we are currently working on).

Below are the responses to the handwritten comments in the main manuscript and supplementary material:

*Re. To whom are caregiving practices shifted away from older children (p. 2).*

We have now specified that we meant to adults.

*Re. What is school-like directed interaction (p.3)?*

We directly refer to the fact that directed interactions are typical in school environments and therefore that caregivers may reserve the language of school instruction for those interactions that are typical in school contexts. See modified first paragraph in page 3 for clarification.

*Re. Comment regarding whether CDS comes from kids (p. 4).*

What we meant is that the majority of input *in general* that infants receive in these communities comes from other children (not just CDS).

*Re. Who performed the transcriptions and for what purposes (p. 4).*

We have modified our description of the methodology used to transcribe the recordings in page 11. Transcriptions were performed by local, bilingual research-assistants, specifically for the purpose of this study and utterances coded by the first and last authors according to the scheme outlined in page 11.

*Re. Comment in page 7 asking whether all caregiver types showed the same trend across cohorts.*

We have now added a reference to Table S7, that shows the proportion of utterances in Spanish received by children, broken down by interlocutor type, which reflects that indeed the trend was consistent across them.

*Re. comment in page 7 asking whether the quote from the interviewee refers only to Mayan*

We believe she meant learning how to speak competently in general (as she answered that in response to our question asking on whether she believed children needed to be actively taught in order to learn how to speak). Linking to the following comment made on page 7, there were indeed two different questions in our questionnaire (see last paragraph of page 7): the first one asked interviewees whether they believed *in general* that children needed to be actively taught in order to learn how to speak, and the second one whether they believed that process to be the same or different in Spanish and Yucatec Maya.

*Re. Distinction between mothers and primary caregivers (p.8).*

In this case all primary caregivers of the children in our sample were their mothers (see last paragraph of page 11).

*Re. Comment regarding what is being measured to conclude that speakers are not devaluing the local language (p.8).*

Such conclusion is based on the fact that all 126 interviewees reported that they believed competences in both languages to be equally important. In addition, the lack of stereotypes and importance ascribed to bilingualism in the region (as opposed to monolingualism in either language; see also Padilla-Iglesias et al. 2020) support this claim.

*Re. Comment in page 9 concerning the statistics showing that caregivers who thought that Maya was easier to learn than Spanish were more likely to use Spanish, and less likely to use Maya, when speaking directly to infants.*

We have now added a reference to Figure 3, which shows the results of the analyses relating caregivers’ beliefs regarding the relative ease of acquisition of Spanish and Yucatec Mayan to the proportion of utterances in Spanish they directed at their infants. The second paragraph in page 8 reports the statistics from the relevant Bayesian GLMMs used to assess such hypothesis.

*Re. Comment in page 10 stating that the expression “purposely devaluing” does not make much sense.*

What we meant with that sentence is that we don’t find evidence in support of an active attempt to replace Yucatec Maya with Spanish rooted in a belief that the latter is more socially or ecologically advantageous. See the modified version of that paragraph.

*Re. Comments on the description of the ethnographic context.*

We have now also altered and expanded the supplementary information text for greater clarity and information.

*Re. Comment in page 11 regarding the classification of utterances into “Spanish” or “Maya”.*

See the updated version of that section as well as the above response to Reviewer 1 concerning the same issue.

*Re. Remark that we had only included the questionnaire in English.*

We have now updated the supplementary material with the Yucatec Maya version of the questionnaire as well.

Reviewer 4

*This paper examines linguistic input in a bilingual setting in Yucatan over time ´ and language attitudes towards the two languages in question. This data is used to test the hypothesis that language shift away from local languages is driven primarily by parents “devaluing” the local language, with the authors arguing that a mix of other factors better explains the shifts witnessed in this community. This is an interesting and worthy topic and the core finding of a shift over time in the input children receive is quite interesting and potentially important. However, I’m pretty unclear what the “devaluation” hypothesis actually is, to what extent it’s a straw man, and whether there’s any substantial positive conclusion to be made about the various alternative factors discussed (e.g. changes in childcare, schooling, parental beliefs about language learning). For these reasons, I cannot recommend this paper for publication. I summarize my basis for this assessment below in A and B.*

We thank the reviewer for their detailed assessment and believe that in responding to the critiques presented by the reviewer we have substantially strengthened the manuscript.

*The paper is structured primarily as an argument against “devaluation” of Maya (or of indigenous and other local languages generally) as the causal factor responsible for shifts in language input. However, I don’t understand what the authors conception of “devaluation” is and therefore find it hard to assess this hypothesis and how it might be linked to the data presented. First off, the extent and ways in which people value or don’t value different languages doesn’t happen in a vacuum. It is itself consequence of any number of complex social, economic, political, and other factors that all interact with other factors, including the ones discussed as competing hypotheses. For example, parents choice of caregivers could be partially driven by how much they value different languages. Second, the valuing of different languages mostly matters relative to other languages. People’s esteem for Maya could be unchanged but if their belief in the value of Spanish increases, this would potentially have similar impact, even though it doesn’t seem intuitive to describe this as “devaluing”. Given how I interpret “devaluing”, I’m not clear how the data on the input have any clear bearing on it. The aspect of the input data that the authors appear to take as related to this hypothesis seems to be the percentage of CDS by parents that is in the two languages. There might be other conceptions of “devaluing” that make direct predictions about this or there might be ways to motivate a connection between my conception and this data. As someone expert in understudied 1 languages, I don’t find there to be a specific well-known “devaluation” explanation assumed in the literature in the way that the authors seem to imply. As such, I think it’s incumbent upon the authors to explain 3 things: 1) what their conception of “devaluation” is, 2) what interactions is does or does not have with other causal factors discussed here, and 3) why it makes particular predictions about the kinds of data considered here.*

We wish to stress that our main argument is not one “against” devaluation. Instead, we try to elucidate that too often the word “devaluation” is used automatically to define process changes in language use following market integration (see references 2-10 in the main text), without a mechanistic exploration of how such process actually unfolds. We argue that it is not justified to conclude that caregivers are trying to replace minority with majority languages because they find the latter more useful (our definition of devaluation) simply by observing that they start using majority tongues when directly addressing their children.

Hence, that is why we believe a multivariate approach involving the different factors that affect caregivers’ decisions on when and how much to use each of their available languages, together with an assessment of their attitudes towards each of the languages and their beliefs about the acquisition process, is a good first step into trying to tease apart the many factors that may push minority languages into extinction. Whilst we are completely aware that the list of factors we have considered here is by no means exhaustive, and hence that other variables potentially have a role and/or interact with those analyzed here, what we wish to highlight is that factors that *do not imply* that caregivers believe that the relative importance/usefulness of minority and majority language differs (such as those relating to the relative ease of language acquisition), may affect the observed trends and even put minority languages in danger of extinction.

We have modified page 10 so that now it states that we “do not find evidence for an active attempt of replacing one language with another due to the perception that the majority language is more socially or economically advantageous”. Hence, by devaluation we mean an active perception that native languages are less useful than majority ones, or that the former have become obsolete following socioeconomic and demographic changes. We hope to have made this clearer also on pages 3 and 8.

*Beyond arguing against the “devaluation” hypothesis, the authors discuss a number of different affirmative proposals for the causes of shifts in language input. Many of theses are quite plausible, but I don’t find that the study really makes a very strong empirical case regarding these different alternatives. I think what would be most convincing is if they could have shown individual-level correlations between language attitudes/use and other social variables and pattern of input. Failing this, showing population-level shifts in these attitudes over the time period would be helpful. However, the data in this paper don’t seem well-positioned to do either of these things.*

Thank you very much for this remark. Indeed, the present article does not identify a single factor responsible for the shift in CDS from Yucatec Mayan to Spanish throughout the study period considered, but instead quantifies, for the first time, *how* language use and, in particular, the language input that children receive changes throughout the process of market integration. We believe the importance of the paper is that the generalised replacement of one language with another due to native speakers’ perception that their language becomes obsolete, which is often assumed, is instead a complex process driven by several factors. We believe that some of our “null results” (e.g. the fact that caregivers *do not* believe the native tongue to have become obsolete or the fact that changes in input are *not* due to a demographic transition accompanying market integration) are perhaps as interesting as the positive results (e.g. the fact that at least partially, language behaviours are driven by beliefs regarding the relative ease of acquisition of each of the languages). We think that the picture that emerges from the combination of such findings is an important contribution to the study of the linguistic (and in general cultural) consequences of the socio-economic transitions many indigenous communities are currently facing worldwide.

Regarding the last point, we agree with the reviewer that the next step in the investigation should be to investigate those factors driving inter-individual variation in language behaviour (within cohorts), and we have performed some pilot analyses in the topic. However, given that our current sample was around 20 kids per cohort, we are in the process of gathering and analysing more data to increase the power of our findings.

*Instead, we have data from a 2019 attesting to language attitudes and very cursory ethnographic information about economic, educational, and other changes between 2007/2008 and 2013/2014. The time period being studied here is one with immense changes economically, socially, and technologically both for the world in general and Yucatan in particular. This paper presents very little ethno- graphic detail of the sort that would be crucial to understanding which of these various factors play more or less important roles. Another demographic factor not asked about and worth considering is whether there are any truly monolingual Maya speakers in regualr social contact with the child and the household. Not knowing the specific towns in question of course, I feel that in 2007 it was likely that many households would have had a grandparent/neighbor/etc. who really didn’t speak Spanish well, whereas in 2019 this may be much less likely.*

See the modified SI text with additional ethnographic information, also including additional information on the language profile of individuals. Nonetheless, even in 2019, there is still a considerable numbeer of Maya monolinguals in the village. In addition, we wish to stress, regarding the need to speak Maya due to the presence of Maya monolinguals, that in *both* time periods the majority of input that children heard was in Yucatec Maya.

By differentiating in our analyses between CDS and overheard speech we are able to disentangle changes in input arising *in general* from a change in the language profile of adults in the village (if more adults speak Spanish, children should be more likely to be exposed to it) from those changes that result from a conscious effort to direct Spanish input to children (despite a preferential use of Mayan otherwise). Even in 2019, most adults reported that Mayan was indispensable in order to communicate in their local villages.

*I recognize that the choice between terms “Maya” and “Mayan” is quite fraught and that there are often no hard and fast rules in which terms to use when in many cases. However, I encourage the authors to rethink their choice of terms here. In several cases, “Mayan” is used in places where I would expect “Maya”. Basically, I think of ‘Mayan’ as meaning something mor like “of or pertaining to the Maya” rather than being Maya. Terms like “Mayan caregivers” don’t really make sense to me given this. I think the one place where I would more stridently push for a change is in the name of the language itself. The language is called “Maya”, not “Mayan”, whether or not the geographic modifier “Yucatec” is added. Yucatec Mayan simply sounds incorrect to me.*

Thank you for this. We have now changed in both the main text and supporting information all references to the language to “Maya”, as well as reserved “Mayan” to meanings relating “or of pertaining to the Maya” (hence, we have also changed references to the people to “Maya”).

*It’d be helpful to have more information on the villages in question. We are told the population and a bit about the economic activities of fathers, but it would be helpful to have a richer picture of the demographics of the towns, how many monolingual Spanish and Maya speaker there are if any, the extent to which “wage labor” here means working outside for extended period of time vs. other things, whether families have members who live more or less full time in Spanish speaking environments such as Cancun.*

See the two new paragraphs added to the SI Text with descriptions of how long the men that go away from their villages to engage in wage labour are away (In general, over 70% of men who engaged in wage labor did so in the form of weekly trips, and hence returned to their local village every weekend). We have also now included data on the linguistic profiles of all interviewees.

*Also, I assume that the individual villages can’t be named for privacy reasons, but I’m confused by the descriptions here. The villages are described as “in the state of Yucatan about 80 miles to the southwest of Cancun (the closest Spanish- speaking urban centre)”. While it’s fair to say that Cancun is an urban center in a way Playa del Carmen, Valladolid, and Tulum are not, it does seem quite relevant that these are all places that offer ample wage labor opportunities in the tourist industry, many of which (especially the higher paying ones) would be predominantly Spanish or English-based. It’d be helpful to clarify this and also to say something about how far the villages are from such places. A village just outside Valladolid is apt to be very different than one just outside Playa del Carmen or one relatively far from all of these.*

Thanks for pointing this out. We meant that Cancun is the largest Spanish speaking urban centre in the area (and have modified the text accordingly). Based on your suggestion we have also provided more information regarding village location in the supplemental materials on page 1:

The villages where data for were collected all have a population of 400-600 inhabitants and are located approximately 50km from the urban center of Valladolid. They are all located along the same road within a 15km radius. Hence, the Spanish-speaking urban centers in the area (namely Valladolid, Tulum, Playa del Carmen or Cancún) are virtually equidistant from all of them.

This is also specified in the second paragraph of the *ethnographic context* section in the main manuscript (page 10).

We thank all the reviewers for their suggestions and once again, we would like to also thank the editor for organising the review process. We do hope you are satisfied with the changes we made according to your recommendations.

Please let us know if there is anything else you would like us to revise. We look forward to your decision.

Best,

Cecilia Padilla-Iglesias, Amanda Woodward, Susan Goldin-Meadow and Laura Shneidman
