# Supplementary material for: Changing language input following market integration in a Yucatec Mayan community
Source: PLoS One. 2021 Jun 21;16(6):e0252926. doi: 10.1371/journal.pone.0252926 (PMC8216532; doi:10.1371/journal.pone.0252926)
Supplement: Attachment — Submitted filename: Response_to_reviewers.docx [file pone.0252926.s027.docx]

*Dear Dr Peña Garay*

First of all, thank you very much for the detailed comments and for giving us the opportunity to revise our manuscript. We believe we have addressed all questions raised by the reviewers and hope you are satisfied with the replies and the new manuscript. In general, we have re-framed the manuscript to eliminate claims of language loss and language replacement (as suggested by reviewer 3), as well as included more detailed ethnographic information, particularly focusing on the changes across cohorts relating to market integration. We have also performed some additional analyses to further enhance the robustness of our findings. Below you can see a detailed account of the changes made, as well as our response to the specific comments made by each of the reviewers.

*Reviewer #1: 
I thank the authors for their attentive responses to my comments. They have responded well to my queries and I would now accept their paper for publication.*

Thank you for your very helpful feedback. We detail below how we have responded to the remaining questions and comments.

*The study reports two key findings, but the interpretation of the results remains problematic due to the inherent complexity of sociolinguistic data. I am especially concerned about the explanation for the decrease in the number of utterances between cohorts (S7), which the authors link to changes in the villages’ market integration. The alternative is that this difference is due to some other difference between the cohorts. The authors’ responded to my initial concern about this decline by noting that:

"In fact, changes in directed input were driven (fully) by decreases in input in Yucatec Maya. Input in Spanish was very rare in the first cohort (mean of 7 utterances per hour) and increased in cohort 2 (80 utterances per hour). Thus, two changes were taking place simultaneously: a reduction in overall speech directed to children and a shift in the language that was used (from Maya to Spanish) in instances where speech was directed to children."*

*The description in the text should be modified to clarify the fact that the decline in input reflected decreases in Maya utterances.*

We have modified the main body text to clarify this point. In the revised manuscript we say on page 6:

To summarize, infants in cohort 2 generally received less directed input than infants in cohort 1 but, when that input was addressed to them, it typically came in Spanish. Indeed, changes in directed input were driven, fully, by decreases in directed input in Yucatec Maya. When we consider the total number of utterances per hour in directed speech heard by infants in each of the languages, we find that infants in cohort 1 heard an average of 397.3 Maya utterances per hour in directed speech, but infants in cohort 2 children heard only an average of 92.51 Mayan utterances.

*The authors might consider replacing table S7 with a table that just reports the number of Maya utterances in directed input across the two cohorts. I combined the numbers in S7 and S8 into a single table in order to simplify the comparison:

Number of utterances S7 Proportion of Spanish S8
mothers Other adults children mothers Other adults children
Cohort 1 99.55 36.86 346.69 10% 1% 27%
Cohort 2 76.81 37.79 165.72 29% 66% 61%

I don’t see how to reconcile the numbers in the authors’ response with the numbers in S7 and 8. For Cohort 1, 10% of 99.55 ~ = 9.9 utterances (close to 7 utts/hr.) For Cohort 2, 29% of 76.81 ~ = 25 utterances (not close to 80 utt/hr.) This confusion suggests it would help if the authors supply the number of utterances in Maya and Spanish as well as the proportions. It’s good practice to provide both numbers and proportions. My calculations suggest that the mothers in Cohort 1 produced a mean of ~90 Maya utterances, and mothers in Cohort 2 produced a mean of ~51 Maya utterances, while their Spanish utterances increased from 9.9 to 25.*

First of all, thank you for the careful check of our results and sorry for the confusion regarding the specific numbers reported in Tables S7-S8 vs. the response to reviewers. What is reported in the manuscript are the posterior predicted mean number of utterances per hour received by children. That is, it is not the “raw” mean from the data but the formal result from the statistical models used. This is also the reason why it is not really appropriate to attempt to calculate “backwards” the raw number of Spanish utterances received by children using *both* the posterior predictive mean number of utterances from such poisson models together with the same predictions from a different kind of model (in this case, the logistic models predicting the proportion of Spanish utterances out of the total number of utterances).

The quote above, from the previous response to reviewers, refers to the raw means of the number of Spanish utterances heard by children – and was provided to illustrate the lack of Spanish input received by infants in cohort 1. To further clarify this issue, we have included a new table (S12 Table) containing the raw average number of utterances for each input type both overall and in Spanish. From this table, the number of Maya utterances *does* correspond to the total number of utterances – the number of utterances in Spanish. Also please keep in mind that the full raw dataset is also attached as a supplementary file.  *These numbers still don’t account for the decrease in Maya utterances between cohorts assuming that the children’s basic interactions wouldn’t change between cohorts. This is one of the findings that as the authors state “Our results show, for the first time, that the quantity, type, source, and language input heard by Yucatec Maya infants changed as the communities became more connected with urban centers, and hence education, wage labour and market goods became more accessible to their inhabitants.” The authors link the decrease in speech from other children to a decrease in time the older children spent with the target children due to schooling (p. 9 and response to reviewer). This explanation doesn’t explain the decrease in the mother’s directed speech between cohorts.*

Since our study does not analyse changes in childcare patterns across cohorts, we do not attempt to make any causal claims relating any specific factors to the changes in interaction patterns. Nonetheless, since we observe that the most drastic source of the reduction in directed speech are other children, we hypothesize that the main factors leading to the decrease in directed speech to infants must relate to those features of market integration that affect either the availability or the behaviour of other children around them. We completely agree that such explanations would not account for the also observed (but very slight) decrease in directed speech from mothers (Estimate=-0.26, 90% HPDI: [ -0.34, -0.18]). Please see the new paragraph we have added to the discussion section (page 10) where we acknowledge and discuss possible reasons driving it as well as the response to the following comment (below) were we detail the additional analyses we performed to clarify this matter.

*If the target children’s behavior changed between cohorts then the target children’s behavior, not the behavior of other speakers, would account for changes in the number of utterances. One change in the target children’s behavior might reflect the difference in the children’s ages between the cohorts (Median 22 mos in Cohort 1 and 18 mos in Cohort 2, S2). These differences could correspond to differences in the mean lengths of the target children’s utterances from a one-word stage to a two-word stage. Thus, differences in the mothers’ CDS between the cohorts might reflect differences in the target children’s language production rather than changes in market integration. Or both factors might be at work.*

*In their response to my previous question about the children’s language production, the authors state that they “did not quantitatively assess children’s proficiency on each of the languages…” This observation makes sense from the perspective that CDS is the primary driver of the children’s language development, but ignores the point I raise here that the children’s language production might account for the decrease in the mothers’ directed speech in Maya. It would be a simple matter to calculate the target children’s mean length of Maya and Spanish utterances in words across the two cohorts to test this hypothesis. An alternative would be to acknowledge the target children’s language as a possible explanation for the decrease in speech between cohorts. It would help to add the authors’ new observations about the children’s current use of Maya and Spanish in order to add a valuable perspective on the limited effects of market integration. It might yet prove to be the case that despite changes in the economy, the villages will persist in their use of Maya.*

We believe that you are raising two possibilities here. First, that there may be a sampling issue between cohort 1 and cohort 2 that could explain our differences in input (due to children of different ages receiving different kinds of input), and second that there might be real differences (not due to sampling) in language production between C1 and C2 that may influence input. In terms of sampling, we note that the differences in age between children in both cohorts was non-significant – it is true that the age ranges were not fully overlapping. Indeed, the age range for cohort 1 was 16.1-24 months whilst that for cohort 2 was 16.1-22.5 months. To rule out the possibility that CDS differences across cohorts could simply be due to differences in the target child’s age, we re-fitted all the models reported in Figure 1 of the main text, excluding from Cohort 1 all 23- and 24-month-old infants (N=2). The results from these analyses further support our hypothesis regarding the fact that the most likely source of cross-cohort differences in CDS are due to differences in CDS from other kids. As in these set of models, whilst the apparent reduction in CDS from mothers disappears, perhaps owing to the fact that mothers are more likely to direct speech to older than younger infants, we still see a significant reduction in the total number of directed utterances infants from the second cohort received stemming from a reduction in utterances directed from other children. We have included this information in the revised manuscript (see new Figure S3 and new supplementary methods in page 11 of the supporting information file, as well as discussion of this matter in pages 5 and 6 of the results section). We believe that together these analyses rule out the possibility that our results are due to differences in sampling.

With regards to the second possibility, that “*children’s language production might account for the decrease in the mothers’ directed speech,”* we fitted a Bayesian multilevel zero-inflated Poisson model identical to those reported in Figure 1a of the main manuscript with “Number of directed utterances per hour received by target child from primary caregiver” as response variable and “Cohort” as well as “Number of utterances produced by the focal child” as predictor variables. We confirm that, again, whilst there was a negative effect of “Cohort” on the number of utterances that infants received from their primary caregivers (Estimate=-0.43; 90%HPDI: [-0.53,-0.33]), the number of utterances produced by the focal child did not affect the number of directed utterances they received from their caregivers (Estimate=-0.01; 90%HPDI: [-0.01,0]).

We have included the specification of this new model in page 11 of the supplementary materials, as well as the results in Table S13 and again in the new results section in pages 5 and 6 of the main manuscript. We believe that, together, these analyses rule out the possibility that our results are due to differences in sampling.

While we do not have a more fine-grained assessment of language ability and the relation to input in the revised manuscript, we now acknowledge this possibility in the discussion on page 10 saying:

Future research might also consider the role of the child’s production in influencing the amount of directed input that he or she receives. While in the current study we did not find a relation between the amount of talk that children produced and input received by others, we did not conduct a more fine-grained assessment of children’s competencies in each language. It could be the case that if decreases in the input directed to children are language-specific (i.e. in Maya), children may become less competent in Maya, and that this in turn might affect the amount of Maya they elicit in directed input.

*Reviewer #3:*

*One of my main suggestions, namely to tone down or background the claims made on the basis of the survey data, the authors chose to ignore, and it is thus difficult for me to reassess the paper given my reservations about its original design, which remains largely unchanged. My original enthusiasm for the paper was linked to empirical finding (1), and the authors’ corrections have at least addressed, if not resolved, most of my doubts about the exact nature of the shift they find over the two-cohort CDS corpora between different kinds of input and the coding they have used to characterize “Maya” as opposed to “Spanish” “input.” (The fact that native speakers have done much if not all of the coding does not assuage my doubts about the categories they have been instructed to code with, but I am happy to let those doubts largely pass for present purposes. Here is just one example, “child directed” and “overheard” can only be mutually exclusive categories if one of them—in this case “child directed”--is based [as it now explicitly is claimed to be] on a disjunctive definition based on a variety different features, from syntax to gaze, and the other—“overheard”—is now a wastebasket remainder category. But surely, in our ordinary life, many utterances explicitly directed at one interlocutor may nonetheless be unabashedly intended to be “overheard” by another, so why is the form decisive rather than the intended effect?) I still believe the empirical result is interesting and important, even without any further speculations about whether or not what parents or other caregivers “believe” about languages is relevant to it, or whether the change is somehow linked to the overall effects of the otherwise largely taken-for-granted process of “market integration” to which the authors refer. It is in this latter context that I agree with other reviewers’ worries about the relative thinness of the ethnographic background in this study—even with respect to the central ethnographic issue of who takes care of children and how-- but it seems to me that the authors are not headed in the direction of trying to question or otherwise tamper with the chronological socio-historical or economic progression with which they have characterized the Yucatec situation.*

*I was and remain considerably less convinced by the authors’ decision to retain the attitude survey study more or less intact. With respect to my earlier complaints, they write that they still find the survey “a useful tool to understand the patterns we observed with quantitative data” and take comfort from their view that their multiple methods “point to the same conclusions.” Given that decision, I have nothing more useful to say on the matter and would prefer to leave to the editors and other reviewers their assessment of publishability.*

Thank you for these comments. First, in regards the survey we would like to reiterate that had the responses to the survey been random, as the reviewer suggested in the first round of revisions, we would not expect them to predict the behaviour of the participants before they participated in the survey. We found that caregivers who reported that they thought Spanish was harder to learn than Maya were the caregivers who selected Spanish, and not Maya, in directed speech to children. We believe the most parsimonious explanation for this is that caregivers who believed Spanish was hard to learn had a strategy to teach children Spanish via directed input. This is, however, a hypothesis, and in the revised manuscript we have adjusted our language to reflect this throughout the manuscript (for example shifting from “our study suggests” to “we hypothesize that”).

In regards to coding decisions, the coders relied on their interpretation of the speaker’s intent using the constellation of features we have listed in the manuscript to determine whether an utterance was directed or overheard (including content of the utterance). While we acknowledge that no coding categorization is perfect, we used the same coding categories for directed and overheard across the two cohorts and thus differences in cohorts should not be explained by the coding categories.

Finally, we have moved additional ethnographic background to the main body of the paper (see response to reviewer 4).

*Reviewer #4:*

*On the positive side, the authors have taken care to adjust the terminology and provide additional geographic details about the village that are relevant context for understanding the study here (my original Point C). On this front, my concerns have been addressed well. I also appreciate the authors’ efforts to more fully articulate the content of the devaluation hypothesis: “an active attempt of replacing one language with another due to the perception that the majority language is more socially or economically advantageous”. This addresses my concern in the sense that I understand much better what the hypothesis is. I do still worry this is a bit of a straw man and would encourage the authors to do more work to support the claim this is a predominant view in the field. Currently, they mostly cite a few fairly obscure papers, (8-10) in the references list, so further support for this would be useful.*

Thank you very much for this. We have now added references 11-15 that represent key papers on the discussion of bilingualism or language shift following processes of globalisation or market integration (see Labov et al. 2016 or Lambert, 1981) as well as empirical studies examining these processes (Kandler et al. 2010; Fernando et al. 2010 and Bunce, 2020). References 16-17 also discuss these processes (making the argument of market integration leading to language replacement) in the specific Mexican setting.

In addition, we significantly re-framed the introduction of the paper to present the devaluation hypothesis as just one of many hypothesises that may explain changes in language input. We hope that this reframing can address the concern of a straw man argument.

*Despite these improvements to the paper to me there are still key things missing. At a macro level, the papers hypothesis as I understand it is that a set of social, economic and other changes—“market integration”—produce changes in attitudes and behavior that lead to language shift. The paper shows in detail a shift in behavior between the two periods of data collection, 2007/2008 and 2013/2014.*

*But as some reviewers including me noted on the original submission, there’s very little work even qualitatively to characterize the changes took place between the two dates and essentially none of it happens in the body text of the paper. Without this, it’s really hard to know how to interpret the changes in behavior. The SI Text has some such information regarding whether father have milpas, whether they work in wage labor, and more anecdotal changes regarding books and TVs. The main text also includes the information that secondary schooling became available in the communities during this same time period. Given that the primary change in language input during the time period is towards less input from other children, couldn’t this plausibly be attributed primarily to the secondary schooling in the community rather than any sort of “market integration”?*

To increase the clarity of the manuscript, we have now added some of the quantitative descriptive statistics regarding socioeconomic changes between cohorts to the results section of the main text. Regarding the effect of secondary schooling on the linguistic input received by infants, please note that we only considered “children” those under 11 years of age. Therefore, secondary school pupils would be counted as adults, and consequently, if the source of the reduction in input were the “no-longer-available” secondary school pupils, we should have seen this reflected in the input from “other adults” (which was not the case). Nonetheless, even if that had been the case, a greater availability of higher education in the majority language is part of the “Integration into global economies suite”, as it is the product of a greater availability of employment options and formal training in the majority language.

*The main text in several places suggests a fairly sweeping claim “that the quantity, type, source, and language input to Yucatec Maya infants changed as the communities became more connected with urban centers, and hence education, wage labour, and market goods became more accessible to their inhabitants”, but across the SI text and the main text doesn’t do nearly enough to me to establish such larger conclusions. And it’s not really clear to me why secondary schooling in the communities constitutes a form of “market integration”. As noted for the original submission, I would personally want to see more detailed work (in the main text) characterizing the shifts in the time period in question.*

Please see our response to the comment above.

*On other side of this picture, the paper’s rhetoric throughout is focused on language shift* and languages dying, yet in responses to the initial reviews and in the SI text, the authors have been explicit that there in fact has not been a shift in the vitality of the language. I think it is inappropriate to frame the paper in terms of language death given that there is no evidence this is in fact taking place. Yucatán has had widespread bilingualism in many places since colonial times and while it is of course *possible these changes are harbinger of language shift, it is far from a given and I would strongly recommend the authors rethink this framing. I find the mindset of assuming that minority languages are doomed to extinction harmful and I really wish the paper tried to avoid this mindset given the lack of empirical support for any shift in progress.*

To address this issue we have significantly restructured the introduction to remove the wording specific to language death, and have reframed the questions presented in the paper to those that 1. assess whether there have been language shifts 2. what these language shifts are, and 3. why they are hypothesized to occur. We restrict our discussion of language loss to a discussion of the potential consequences of these changes in input and clarify that the idea that market integration processes inevitably result in the devaluation and subsequent replacement of minority languages with majority ones is a non-tested, potentially harmful assumption. (page 4 and page 10).

*To summarize, I think this paper presents an interesting set of core findings about language attitudes and changes in language input. However, it consistently presents itself as an assessment of how socioeconomic changes produce language shift and loss of language diversity while providing cursory evidence of socioeconomic change and no evidence of language shift.*

Please let us know if there is anything else you would like us to revise. We look forward to your decision.

Best,

Cecilia Padilla-Iglesias, Amanda Woodward, Susan Goldin-Meadow and Laura Shneidman
